# New molecular components of high and low affinity iron import systems in *Drosophila*

**DOI:** 10.1038/s41467-025-60758-6

**Published:** 2025-07-01

**Authors:** Sattar Soltani, Minyi Yan, Qingxuan Yu, Areeg Abd Elhafiz, Erika Pfriem, Samuel M. Webb, Thomas Kroll, Jahir Marceliano Bahena Lopez, Fanis Missirlis, Kirst King-Jones

**Affiliations:** 1 Department of Biological Sciences, University of Alberta, Edmonton, AB Canada; 2 Stanford Synchrotron Radiation Lightsource, SLAC National Accelerator Laboratory, Menlo Park, CA USA; 3 Departamento de Fisiología, Biofísica y Neurociencias, Centro de Investigación y de Estudios Avanzados (Cinvestav), Mexico City, Mexico

**Keywords:** Iron, Transcriptomics, RNAi, Membrane proteins

## Abstract

The high abundance and molecular versatility of iron have led to its universal presence in biological systems, yet its absorption is exceptionally challenging. Animals and yeasts use divalent metal transporters to import iron, but yeasts also employ the multicopper oxidase Fet3p for high-affinity iron uptake when iron-starved. Using long-term iron depletion in *Drosophila*, we identified four components involved in iron absorption: Multicopper oxidase-4 (Mco4), a Fet3p ortholog, is essential for surviving iron starvation, whereas the cytochrome b561 enzymes Fire (Ferric Iron Reductase) and Fire-like, as well as cytochrome b5 protein Firewood, are required for iron absorption under normal conditions. This study reports the presence of a high-affinity iron uptake system in an animal, a cytochrome b5 electron donor for ferric iron reduction, and intestinal ferric reductases, and provides a valuable resource for further exploration of genes involved in iron homeostasis, transport, and absorption.

## Abstract (web summary)

Although iron is essential, its absorption is inefficient; this study uncovers distinct iron uptake strategies in *Drosophila*, including a previously unrecognized high-affinity system activated during iron starvation.

## Introduction

Life on Earth depends on iron^1^, a redox-active transition metal that donates and accepts electrons in processes such as oxygen transport, detoxification, energy production, and nucleotide synthesis. The vast majority of biological iron is used to produce protein cofactors, i.e., heme and iron-sulfur (Fe-S) clusters^2–4^, whereas some proteins bind the metal directly as ionic mononuclear or dinuclear iron^5^. Despite its biological importance, iron has poor bioavailability due to its intrinsic insolubility, particularly in aerobic environments. This low solubility poses a significant barrier to its absorption by organisms. For instance, a typical human adult absorbs only about 2 mg of iron per day, which represents ~0.05% of the body’s total iron content.

Not all cells require equivalent amounts of iron. For example, erythroblasts require excess iron to synthesize hemoglobin and differentiate into iron-rich erythrocytes, steroid hormone-producing glands (such as the *Drosophila* prothoracic gland, hereafter “PG”), and tissues involved in detoxification responses (such as liver cells) are also typically iron-rich, because their metabolism relies on high levels of cytochrome P450 enzymes, which require heme cofactors to function. As such, iron uptake, distribution and sequestration require cell-type-specific regulation to match the iron needs of individual tissues.

Iron pathways are partially conserved between humans and *Drosophila*^6–8^. In vertebrates, duodenal enterocytes are the first site of iron regulation as they govern the uptake and release of absorbed iron^2,8,9^. Duodenal cytochrome B (DCYTB), a transmembrane ferric reductase of the cytochrome b561 (CYB561) family, uses electrons donated by cytoplasmic ascorbate to reduce ferric iron (Fe^3+^) in the gut lumen to ferrous iron (Fe^2+^). DCYTB has two *Drosophila* homologs, CG1275 and Nemy. Of the two, CG1275 appears to be the ortholog of DCYTB, as it has 43% identity compared to 33% identity exhibited by Nemy^10^. DCYTB knockout mice (*Cybrd1*^*−/−*^) did not show iron deficiency compared to controls, suggesting that either other ferric reductases operate alongside DCYTB^11^, or that dietary reductants can compensate for its loss. Besides *CG1275* and *nemy*, six other CYB561 genes are found in *Drosophila* (*CG3592*, *CG8399*, *CG10165*, *CG10337*, *CG13077* = *fire-like*, and *CG13078 = fire*)^10^. Of the eight CYB561 proteins, only Nemy has been studied in greater detail, which revealed that *nemy* mutants have reduced memory retention, consistent with the presence of Nemy in synaptic vesicles^12,13^. A recent report described an assay to detect ferric reductase activity in the larval intestinal lumen, but the gene encoding the ferric reductase remains unknown^14^.

Once Fe^3+^ is reduced to Fe^2+^ via DCYTB, iron is transported by vertebrate Divalent Metal Transporter 1 (DMT1 aka SLC11A2) into the enterocyte, a process that is likely mirrored in *Drosophila* by the DMT1 ortholog Malvolio^15^. DMT1 is permeable to a wide range of divalent metals and, therefore, cannot efficiently import iron if it is scarce relative to other metals^16^. DMT1 not only plays a role in intestinal iron uptake but is also needed for iron release from late endosomes/lysosomes^17^. Consequently, DMT1 knockout mice (*SLC11A2*^*−/−*^) die within a week from severe anemia^18^. An intestine-specific DMT1 knockout (*DMT1*^*INT/INT*^), however, is viable, but animals are anemic, display significantly reduced iron concentrations in various organs, and develop cardiomegaly and other heart problems after six months^19^. These data suggest that DMT1 is critical for iron uptake but does not account for all absorption of non-heme iron in the gut.

Ferrous iron leaves the vertebrate enterocyte via Ferroportin, for which no *Drosophila* ortholog exists. Upon immediate oxidation by Hephaestin or Ceruloplasmin, which represent two of the three vertebrate Multicopper oxidases, ferric iron is loaded onto the serum protein transferrin (Tf) and delivered to target tissues^20^. Iron-loaded Tf binds to the membrane-bound Transferrin Receptor 1 (TfR1) in target tissues, after which the complex is endocytosed, where another ferric reductase allows the release of ferrous iron into the early endosome and its subsequent transfer into the cytosol via a different DMT1 isoform^21–24^. *Drosophila* has three Tf orthologs (Tsf1-3), of which Tsf1 has been shown to deliver iron across the hemolymph to reach target tissues^25^. Curiously, flies lack an ortholog of vertebrate TfR1, suggesting the existence of an uncharacterized insect transferrin receptor.

DMT1 and unrelated proteins with similar functionality in other organisms, such as yeast Fet4p, are sufficient to meet cellular iron requirements under iron-replete conditions despite their low affinity for iron. Under iron-deficient conditions, however, bacteria, fungi, and plants employ two basic strategies to compensate for the inefficiency of low-affinity iron uptake systems. The first strategy uses siderophores, which are secreted iron-chelating molecules that are later recaptured. This process has been observed in bacteria, fungi and graminaceous plants^26,27^. The second strategy utilizes a plasma membrane-bound ferric reductase that reduces extracellular Fe^3+^ to Fe^2+^, and is followed by import into the cell via a high-affinity transport system. In non-graminaceous plants, IRT1, a Zip family member, acts as a high-affinity transporter for Fe^2+^ in roots^28,29^. In the yeast *Saccharomyces cerevisiae*, three proteins ensure high-affinity iron import. The first step is carried out by the ferric reductase Fre1, which converts extracellular ferric iron to ferrous iron. Fe^2+^ is then re-oxidized by Fet3p to Fe^3+^, which is coupled to Ftr1, a permease, which then transports Fet3p-derived Fe^3+^ into the cell. Fet3p is a critical component of this triple-step reduction/oxidation reaction, leveraging distinct iron-binding properties to ensure selective iron uptake^30^.

Despite our continuously growing understanding of iron biology and its regulation, our grasp of genome-wide networks that respond to changes in iron levels remains rudimentary. Several microarray studies from various model organisms and cell lines have attempted to characterize the cellular responses to either iron deficiency or iron overload^31–40^. Nonetheless, many of these studies have limitations because they focus solely on the long-term effects of iron overload or deprivation and often examine only a single tissue type. This overlooks the possibility that different tissues might exhibit unique responses to changes in iron levels. Consequently, while end-point measurements provide a straightforward experimental approach, they may fail to capture acute transcriptional responses that occur within hours. We reasoned that one should examine the alimentary canal separately since the gut is the principal site of iron absorption and, therefore, likely has a unique transcriptional profile.

In an earlier study, we showed that mild iron depletion may take multiple generations to elicit a phenotype^41^. In this study, we exploited this treatment to gently deplete wild-type flies of iron over multiple generations. Specifically, we reared flies for five generations on fly media that contained the iron chelator Bathophenanthroline disulfonic acid (BPS) to reduce whole-body iron content to a level that would sensitize animals to sudden increases in dietary iron concentrations, which we accomplished by transferring larvae to fly food supplemented with ferric ammonium citrate (FAC). We then generated RNA-Seq-based gene expression profiles for three different tissue types at four different time points. Lastly, we conducted validation experiments for differentially expressed genes (DEGs) based on genetic and molecular studies of RNAi, mutant and transgenic flies. As source material for the RNA-Seq studies, we used i) the larval brain ring gland complex (BRGC), because it harbors the iron-rich PG, ii) the gut, as it is the site of iron absorption, as well as iii) whole larvae (aka whole body = WB) to monitor genes in all tissues. The data that were derived from these studies revealed genes acting in iron biology, and provide a comprehensive genome-wide resource aimed at expanding our understanding of iron gene networks and their role in iron biology.

## Results

### Trans-generational iron depletion in *Drosophila* larvae

**Fig. 1:**
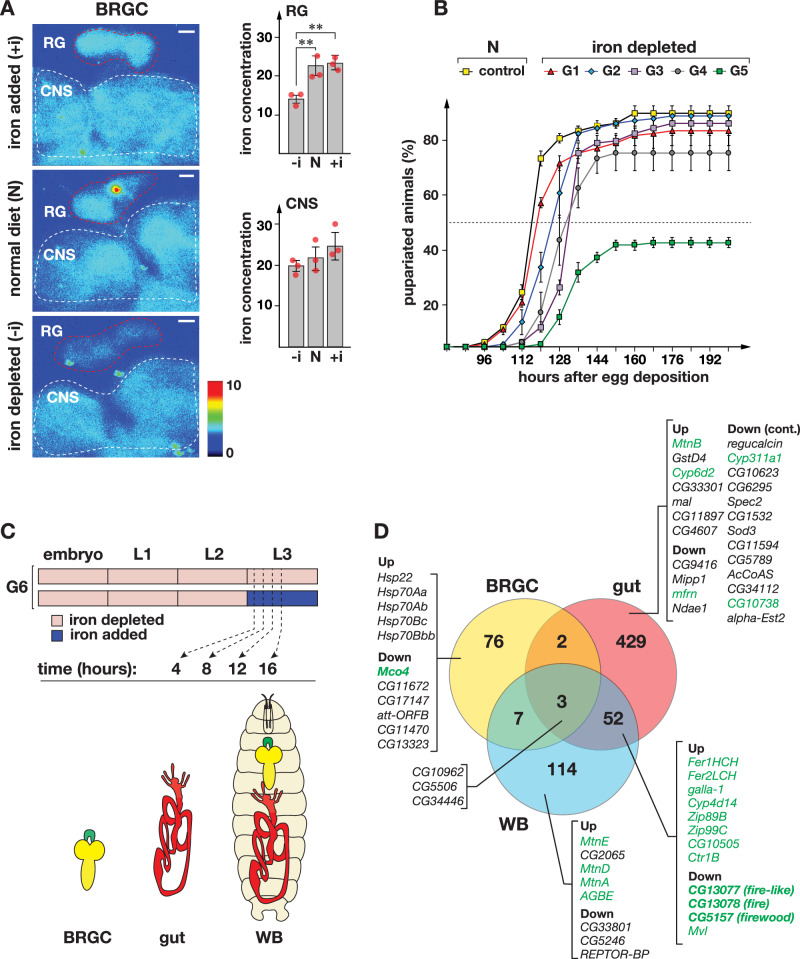
RNA-Seq strategy to examine genomic response to dietary iron. **A** X-Ray Fluorescence Microscopy (XRF) images of BRGC (brain-ring gland complex) samples and quantification of iron levels in the ring gland (RG) and central nervous system (CNS). Larvae were reared for one generation on iron-enriched (+i), normal (N), and in iron-depleted (−i) media, and BRGCs were dissected from L3 larvae. Average iron concentrations in the RG, (red dotted line) and CNS (white dotted line) were calculated from three replicates based on Kα emission/area ratios. Dotted lines indicate regions used to measure iron levels. Scale bars: 30 µm. The color scale bar reflects log2-based iron levels. Asterisks denote significance thresholds (*p < 0.05 and ***p < 0.001). **B** Survival rates and developmental progression of fly populations as a result of multi-generational iron deprivation. *w*^*1118*^ flies were reared for five generations (G1–G5) on either a normal diet (N) or media supplemented with BPS. Y-axes denote the percentage of pupariated animals, and X-axes show hours after egg deposition. Dotted line indicates 50% pupariation. Error bars indicate standard deviation from three biological replicates; means are centered. **C** Schematic of the RNA-Seq experimental design. Eggs from G5 flies reared on iron-depleted media (see **B**) were used to produce the G6 generation. G6 larvae were staged within 30 min after the L2/L3 molt and split into two groups: one cohort was transferred to iron-supplemented food (FAC) and the other to fresh iron-depleted media. At 4, 8, 12, and 16 h after the L2/L3 molt, BRGC (Ring gland = green, CNS = yellow), guts (red) and whole larvae (WB = whole body) were collected. L1/L2/L3 denote first, second, and third instar larvae. **D** Venn diagram summarizing RNA-Seq results for a total of 683 differentially expressed genes for i) the BRGC (88 genes), ii) the gut (486 genes) and iii) WB (176 genes). Example genes shown for selected sections. Green: known iron-related genes. Source data are available in the accompanying source data file.

Prior to pupariation, *Drosophila* 3rd instar larvae feed continuously for ~36 h, and require large nutrient inputs to sustain rapid growth^42,43^. When larvae were switched from a normal to an iron-rich diet, we observed only weak to moderate transcriptional changes in known iron-regulated genes (Supplementary Fig. S1A), consistent with the finding that one generation of high iron-feeding did not significantly raise ring gland or CNS iron levels, as assessed by synchrotron-based X-ray fluorescence microscopy (Fig. 1A). Larvae reared on BPS-containing media showed reduced ring gland iron levels but largely unchanged brain iron concentrations (Fig. 1A). These results confirm earlier findings that tissues differ in their iron responses^44^, supporting the “sparing model”, where the CNS is protected from starvation relative to other tissues^45^. Although iron depletion affected the ring gland within one generation, larval developmental timing was largely unaffected (Fig. 1B), suggesting that iron remained sufficient for gland function.

To minimize potential BPS toxicity or non-specific effects, we used moderate BPS concentrations and maintained fly populations on supplemented media over several generations, gradually lowering systemic iron to critical levels. From generation #2 (G2) onwards, animals pupariated with increasing developmental delays, adding about 4–8 h per generation until G4 (Fig. 1B). Overall survival rates were similar between controls and G1-G3 populations on BPS media. G4 animals showed a moderate drop, and in G5 survival fell sharply (only 42% reached adulthood) with surviving larvae undergoing puparium formation ~32 h later than controls (Fig. 1B). G6 animals fully recovered when transferred to media supplemented with both BPS and FAC (Supplementary Fig. S1B), indicating that iron stores were quickly replenished and that prolonged BPS exposure caused no cumulative toxicity beyond iron chelation.

### RNA sequencing of larval tissues isolated from G6 larvae

After we had established that five generations of iron deprivation caused sufficiently low systemic iron levels to reduce survival rates to <50%, we reasoned that re-feeding with a high-iron diet in G6 would have a more pronounced transcriptional iron response than doing so in earlier generations. We, therefore, continued to rear G6 larvae on BPS media until the L2/L3 molt (~72 h after egg deposition) but then divided animals into two groups, where one was transferred to fresh BPS-supplemented food as a control group, whereas the experimental group was switched to an iron-rich medium (Fig. 1C). To minimize biological variability, we precisely staged all larvae at the L2/L3 molt, i.e., newly formed L3 were transferred within 30 min to either fresh BPS- or FAC-supplemented food. To attain robust gene expression profiles, we analyzed a time course rather than a single time point. To this end, we collected gut, WB and BRGC samples at 4, 8, 12, and 16 h after the L2/L3 molt. Upon RNA sequencing (Illumina Hi-Seq, three lanes), we obtained an average ~22 million paired-end reads per sample. The total number of RNA-Seq samples was 48 (4 time points ×2 media types ×3 tissue types ×2 replicates).

We analyzed the RNA-Seq data using *Arraystar* in conjunction with *MS Access*, which we counterchecked via the R-packages *edgeR* and *DESEq2* ^46–48^ (Supplementary Data 2). The tables and figures shown in this study are based on the *Arraystar* analysis, unless stated otherwise. Overall, our approach worked very well, evidenced by the high abundance of known iron players (Fig. 1D, green gene names). In total, we identified transcripts of 486 iron-responsive genes in the gut, 176 genes in WB samples, and 88 genes in BRGC samples (Fig. 1D). In brief, the data revealed the expected upregulation of known metal detoxification genes in the gut and whole-body samples and the suppression of genes with hitherto undocumented roles in iron uptake, as well as iron responses in the brain and ring gland. In the next three sections, we provide a detailed breakdown of our findings based on sample type.

### Gut iron response

We expected that the intestine would have the fastest response time to iron since it is the site of nutrient absorption. In total, we identified 486 genes that responded to a switch in dietary iron concentrations. When we further filtered this cohort for genes showing a rapid and sustained response in the gut [a >2-fold change in the first 4 h and showing the same trend (i.e., consistently up or down) in the remaining three time points], we identified 14 upregulated and 38 downregulated protein-encoding genes (Table 1). Of the 14 rapidly upregulated genes, 11 had established links to iron/metals (see Supplementary Data 3 for our iron/metal reference list), and so had 7 of the 38 downregulated genes. Term enrichment statistics for iron/metal genes are depicted in Table 2, which also lists other terms, including enrichment for “cytochrome P450” genes, “ABC transporters” and “transmembrane transport” in the gut data.

**Table 1:** Rapid and sustained gene responses in the gut (52 genes)

Upregulated (14 genes)						
**Name**	**Description**	**Reported link to metal/iron?**	**4 h**	**8 h**	**12 h**	**16 h**
**MtnB**	Metallothionein	Yes	13.6	3.6	10.7	3.0
**CG10505**	ABC transporter	Yes (homolog of yeast cadmium factor)	13.0	1.6	6.1	2.5
CG7763	Lectin fold / carbohydrate binding	No	11.2	2.1	5.2	3.5
**galla-1**	Fe-S cluster biosynthesis	Yes	7.6	4.4	4.8	2.5
Drip	Aquaporin family	Some data links Aquaporin-4 (Drip ortholog) to metal intoxication	5.0	2.3	4.9	1.8
**Zip99C**	Zinc/iron permease	Yes	4.4	2.5	3.6	1.9
**Cyp6d2**	Cytochrome P450 6d2	Yes (harbours heme)	3.7	2.9	2.5	1.3
**Fer1HCH**	Ferritin 1 heavy chain	Yes (stores iron)	3.1	4.0	7.5	4.5
GstD2	Glutathione S transferase	Yes (induced by Zn and Cadmium)	3.0	4.3	2.2	1.0
CG7720	Sodium:iodide symporter	No	2.8	2.2	3.1	1.1
rdog	ABC transporter	Yes (zinc detoxification)	2.7	1.1	1.8	1.2
**Fer2LCH**	Ferritin 2 light chain	Yes (stores iron)	2.4	3.5	8.9	4.0
**Zip89B**	Zinc/iron permease	Yes	2.4	1.4	3.7	3.2
CG31288	Possible ecdysteroid kinase	Yes (induced by Mn deficiency)	2.0	1.6	1.4	1.2

Downregulated (38 genes)						
**Fire (CG13078)**	Ferric reductase	Yes	−45.1	−7.1	−40.8	−78.1
**Fire-like (CG13077)**	Ferric reductase	Yes	−27.3	−11.3	−19.3	−52.1
**Firewood (CG5157)**	Cytochrome b5	Yes (predicted)	−22.1	−5.4	−7.7	−15.2
CG14205	acyltransferase	No	−8.0	−2.0	−1.8	−1.4
CG5892	acyltransferase	No	−7.5	−2.6	−1.8	−3.3
MFS1	Solute carrier family 17	No	−5.6	−2.2	−1.3	−1.7
Adh	Alcohol dehydrogenase	No	−5.2	−2.4	−1.2	−1.3
**Mvl**	divalent metal ion transporter	Yes, DMT1 homolog	−4.5	−2.3	−2.1	−3.3
CG9416	Metallopeptidase	Yes (zinc ion binding)	−4.3	−2.1	−1.1	−2.1
CG11576	Solute carrier family 52	No	−4.0	−1.9	−1.8	−2.7
alpha-Est2	Carboxylesterase	No	−4.0	−2.8	−1.3	−1.3
**mfrn**	Mitochondrial iron import	Yes	−4.0	−1.6	−1.1	−2.4
CG13893	Phospholipid transport	No	−3.2	−2.0	−1.1	−1.1
Ndae1	Solute carrier family 4	No	−3.2	−1.7	−1.0	−1.2
regucalcin	CG1803	No	−3.1	−2.5	−1.3	−1.1
CG2543	Folylpolyglutamate synthetase	No	−3.1	−1.3	−1.1	−1.7
**Cyp311a1**	Cytochrome P450	Yes (heme-binding)	−2.9	−2.8	−1.3	−1.1
CG18179	peptidase	No	−2.8	−5.4	−3.1	−4.6
CG10623	Methionine synthesis	Yes (zinc ion binding)	−2.8	−1.6	−1.2	−1.3
CG7589	Secretory chloride channel	No	−2.7	−2.1	−1.3	−1.6
CG6295	Triacylglycerol lipase	No	−2.7	−2.7	−1.0	−1.5
Spec2	small GTPase binding	No	−2.5	−2.3	−1.1	−1.7
Mal-A8	Solute carrier family 3	No	−2.5	−3.2	−1.2	−1.2
CG1532	Glyoxalase	No	−2.5	−2.2	−1.5	−1.8
Sod3	Superoxide dismutase	Yes (copper/zinc-binding domain)	−2.4	−2.1	−1.4	−1.8
CG11594	FGGY carbohydrate kinase	No	−2.4	−1.7	−1.0	−1.1
CG5789	ABC transporter-like	No	−2.3	−1.4	−1.2	−1.8
AcCoAS	acetyl-CoA ligase	No	−2.2	−1.7	−1.1	−2.0
CG34112	No data	No	−2.2	−1.8	−1.1	−1.0
**CG10738**	CG10738	Yes (heme NO binding associated)	−2.2	−2.3	−1.0	−1.2
CG8839	Fatty acid catabolism	No	−2.2	−1.7	−1.0	−1.1
Jon99Fii	endopeptidase	No	−2.1	−1.9	−1.5	−1.3
CG7470	Glutamate 5-kinase	No	−2.1	−1.6	−1.1	−1.4
CG15773	No data	No	−2.1	−2.4	−1.1	−1.2
CG17119	Amino acid transporter	No	−2.0	−1.6	−1.1	−1.2
CG8080	NAD+ kinase	No	−2.0	−1.6	−1.0	−1.0
CG13654	No data	No	−2.0	−2.4	−1.1	−1.5
CG33056	Purine metabolism	No	−2.0	−1.3	−1.0	−1.7

The table lists 14 upregulated and 38 downregulated genes in the gut. The gene cohort was obtained by filtering for a >2-fold change at the 4-h time point, combined with a consistent expression trend at the remaining time points. Gene names in bold indicate genes with known or predicted roles in iron metabolism.

**Table 2:** Term enrichment statistics for all gene cohorts

Name of cohort (size of cohort out of 14,557 genes)	Metal/iron (839)			P450 (89)			ABC (57)			TM transport (638)			Chitin (346)			Heat-shock (32)		
	O	E	P	O	E	P	O	E	P	O	E	P	O	E	P	O	E	P
Gut Rapid UP (14)	**8**	**0.8**	**1.5E−16**	1	0.1	1.7E−03	**2**	**0.1**	**8.2E−17**	**6**	**0.6**	**2.0E−12**	0	0.3	ns	0	0.0	ns
Gut Rapid DOWN (38)	7	2.2	8.0E−04	1	0.2	ns	1	0.1	2.7E−02	**8**	**1.7**	**5.0E−07**	0	0.9	ns	0	0.1	ns
Gut UP (77)	12	4.4	2.1E-04	1	0.5	ns	2	0.3	1.9E−03	10	3.4	2.2E−04	1	1.8	ns	1	0.2	4.3E-02
Gut DOWN (322)	31	18.6	2.6E−03	5	2	2.8E−02	2	1.3	ns	23	14.1	1.4E−02	3	7.7	ns	0	0.7	ns
WB Rapid UP (14)	**9**	**0.8**	**5.4E−21**	0	0.1	ns	1	0.1	5.2E−05	4	0.6	9.7E−06	1	0.3	ns	0	0.0	ns
WB Rapid DOWN (10)	4	0.6	3.4E−06	0	0.1	ns	0	0.0	ns	1	0.4	ns	0	0.2	ns	0	0.0	ns
WB UP (53)	**12**	**3.1**	**1.3E−07**	0	0.3	ns	1	0.2	ns	7	2.3	1.7E−03	1	1.3	ns	0	0.1	ns
WB DOWN (41)	7	2.4	1.9E−03	2	0.3	4.5E−04	0	0.2	ns	3	1.8	ns	0	1.0	ns	0	0.1	ns
RG Rapid UP (0)	0	0	n/a	0	0	n/a	0	0.0	n/a	0	0.0	n/a	0	0.0	n/a	0	0.0	n/a
RG Rapid DOWN (4)	0	0.2	ns	0	0	ns	0	0.0	ns	0	0.2	ns	1	0.1	3.0E−03	0	0.0	ns
RG UP (16)	1	0.9	ns	0	0.1	ns	0	0.1	ns	1	0.7	ns	1	0.4	ns	**7**	**0.0**	**7.2E−303**
RG DOWN (40)	2	2.3	ns	0	0.2	ns	0	0.2	ns	0	1.8	ns	**14**	**1.0**	**6.6E−42**	0	0.1	ns

Number in parentheses indicate cohort sizes. “Rapid up” and “Rapid down” cohorts were obtained by filtering for a > 2-fold change at the 4-hour time point, combined with a consistent expression trend at the remaining time points. “Up” and “Down” cohorts defined by requiring at least 3 out of 4 time points to be either up- or downregulated. Bold: *p*-value < 10^−6^.

*O* observed overlap, *E* expected overlap, *P* two-sided *p*-value (Chi square test), *TM* transmembrane, *WB* whole body, *ns* not significant, *n/a* not applicable.

From a homeostatic point of view, one would expect to find genes involved iron/metal detoxification to be upregulated when an iron-rich diet is used. By contrast, genes acting in iron uptake and trafficking should be downregulated based on the rationale that such genes must be more highly expressed in an iron-poor environment to compensate for iron scarcity by increasing the capacity for iron capture. Consequently, a switch to a high-iron diet should decrease their expression. Overall, these anticipated trends held true for the gut data. For instance, among the upregulated transcripts were two genes that encode the main ferritin subunits (Fig. 1D, Table 1), ferritin heavy (Fer1HCH) and light (Fer2LCH) chain homologs. Ferritins are molecular nanocages that store excess cytosolic iron, with a capacity of storing hundreds to thousands of oxidized iron atoms per cage^44,49^. Insect ferritins are generally composed of 12 ferritin heavy and 12 light chain proteins^50^, consistent with our finding that the genes for both subunits are upregulated on high iron diets.

Another transcript we expected to be rapidly upregulated was Zip99C (also known as Zip13), a member of the ZIP family of transporter proteins^51,52^. Zip13 is tightly linked to ferritin function and is the only identified iron transporter/exporter acting in the ER/Golgi axis. Zip13 is thought to mainly act in the midgut and operates by transferring cytosolic ferrous iron into the ER/Golgi, where iron can be incorporated into ferritin^53^. Consistent with our results, Zip13 showed moderate upregulation in an earlier study (1.5-fold) when animals were fed an iron-rich diet^54^. Due to our prolonged iron deprivation conditions, however, Zip13 displayed a considerably stronger transcriptional response, ranging from 1.9- to 4.4-fold induction (Table 1). Interestingly, we identified a second ZIP family member (Zip89B) among the 14 rapidly upregulated genes. Both Zip89B and Zip99C/Zip13 belong to the SLC39 subfamily, which comprises 14 members in *Drosophila*. Zip89B has been proposed to act as a low-affinity zinc transporter^55^, raising the idea that this transporter may have additional substrates, including iron, and may act in a similar fashion to Zip99C/Zip13, albeit in a different subcellular compartment^56^, to facilitate iron detoxification.

Two other upregulated genes, *CG10505* and *MtnB*, which encode an ABC transporter and Metallothionein (Fig. 1D), respectively, are also likely acting in iron detoxification. CG10505 is homologous to several yeast ABC transporters acting in detoxification, including VMR1 (vacuolar multidrug resistance 1) and YCF1 (yeast cadmium factor 1)^57^, and has been identified in fly cell culture systems to respond to metals^35^. *Metallothionein B* (*MtnB*), a metal-binding protein previously shown to act in iron detoxification^58^. Additional rapidly upregulated gut transcripts included *galla-1*, which, like its human homolog CIA2A acts in iron-sulfur cluster biosynthesis^59,60^, and *CG31288*, which is thought to encode an ecdysteroid kinase. Earlier research indicated that manganese deficiency induces this gene^61^.

As explained earlier, we expected that the set of 38 downregulated genes should be enriched for transcripts encoding proteins involved in iron uptake. Consistent with this, we found two well-characterized genes in this cohort, *Malvolio* (aka *Mvl*, the fly ortholog of DMT1) and *mitoferrin* (*mfrn*), which functions in mitochondrial iron uptake^62^. Remarkably, the three most strongly downregulated genes have not been characterized in *Drosophila* and encode two proteins of the cytochrome b561 family (CG13078 and CG13077), as well as a cytochrome b5 protein (CG5157). This was intriguing, since the insect gut ferrireductase(s) responsible for reducing Fe^3+^ to Fe^2+^ were unidentified, but presumed to be either CG1275 or Nemy^8,20^. However, the latter showed no significant response to dietary iron changes, suggesting that CG13078 (hereafter referred to as “Fire“) and CG13077 (hereafter referred to as “Fire-like”) represent the missing ferrireductases. Given the similarly strong response of CG5157 (hereafter referred to as “Firewood”), we hypothesized that this Cytochrome b5 protein provides the electron required for the reduction of Fe^3+^. We will present functional data on these three genes after we have addressed the genomic response in whole body and BRGC samples.

**Fig. 2:**
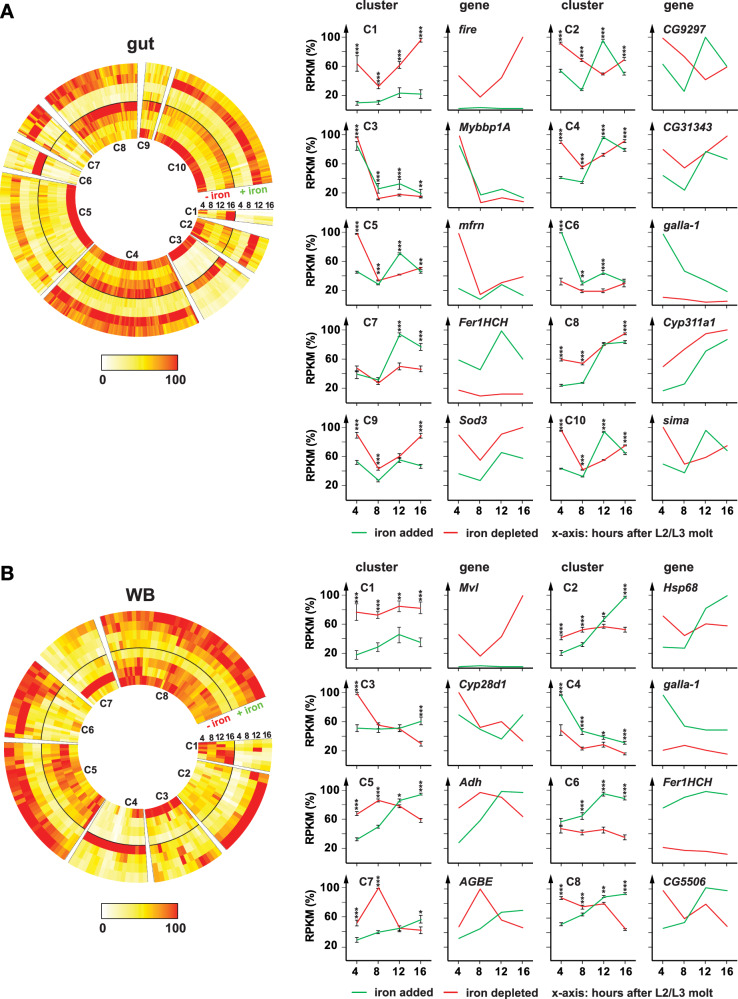
Differentially expressed genes in response to dietary iron supplementation in the gut and whole-body samples. **A**, **B** Cluster analysis of 486 and 176 differentially expressed genes in the gut and whole body (WB), respectively. On the left, 10 clusters (C1–10) and 8 clusters (C1–8) are shown as circular heat maps. For each gene, the highest RPKM value was set to 100 (red), with the remaining seven time points normalized to this maximum. Numbers indicate hours after the dietary switch. The inner four circles depict iron-depleted conditions (“−iron”), and the outer four circles show iron-supplemented conditions (“+iron”). The graphs on the right show cluster profiles (C1–10 for gut and C1–8 for WB), each paired with a representative gene. All graphs show normalized RNA-Seq expression data. Time points (x-axis) indicate hours after the dietary switch. For each gene, the highest RPKM value was set to 100%, and the remaining time points were scaled proportionally. Each cluster profile represents the average relative expression (% of maximum) across all genes in that cluster at each time point. Error bars indicate the standard error at each time point. Asterisks denote significance based on a two-sided Student’s *t* test (*p < 0.05 and ***p < 0.001). Source data are provided in the accompanying source data file.

Our multi-conditional RNA-Seq data permitted us to perform cluster analysis, in order to identify genes that display comparable responses and are thus candidates for being co-regulated. Using this strategy for the 486 genes in the gut cohort, we detected ten clusters (Fig. 2A and Supplementary Data 4). We highlight three clusters that were enriched for previously known iron genes and appeared to be co-regulated in response to our experimental treatments. Cluster #1 comprised eight genes presumably involved in iron uptake, as it included *Malvolio* (the DMT1 ortholog) and *fire*, *fire-like*, *firewood* (Supplementary Data 4). This result provides further support to the idea that the function of the *fire* gene complex is linked to dietary iron absorption via Malvolio/DMT1. Cluster #6 (18 genes) represented the immediate response genes to iron treatment, which included genes encoding transporters *Zip48C*, *Zip89B*; *Zip89C*, *CG10505*, the cytosolic iron-sulfur cluster protein *galla-1*, *MtnB* as well as other highly induced genes such as *Drip*, *GstD2* and *rdog*. *Fer1HCH* and *Fer2LCH* were both part of cluster #7 (22 genes), which showed peak expression at the 12-h time point. This cluster also included Copper transporter 1B (*Ctr1B*).

### Analysis of whole-body samples

In total, we identified 176 genes in WB samples that responded significantly to changes in dietary iron. Of these, 55 genes overlapped with the 486 genes from the gut cohort, including *fire*, *fire-like* and *firewood* (Fig. 1D). The 176 WB cohort was also enriched for iron/metal genes (Table 2). Cluster analysis grouped the *fire* gene complex and *Malvolio* again into a single group (cluster #1, 8 genes), just like we saw in the gut cluster #1. Overall, a total of six genes were common to both clusters, since two additional genes, *CG5892*, which encodes a putative acyl transferase and *CG18179*, encoding a predicted serine protease, were found in either cluster, despite being the result of completely independent experiments (Fig. 2B, Supplementary Data 4).

A set of three metallothionin genes, *MtnE*, *MtnD* and *MtnA* belong to genes uniquely affected in the WB cohort that showed upregulation on high iron diets. *MtnE* and *MtnD* were found in WB cluster #4, which was otherwise very similar to the early-responding gut cluster #6, since it also harbored *Zip99C*, *Zip89B*, *galla-1*, *Drip*, *GstD2*. By contrast, *MtnA* clustered with *Fer1HCH*, *Fer2LCH* and *Ctr1B* (cluster #6, 19 genes), which was overall similar to the late responders in gut cluster #7, whereas *MtnB* appeared to be gut-specific. These findings suggest tissue compartmentalization among metallothioneins, combined with coordinated temporal regulation. Also specific to the non-gut WB cohort was *AGBE*, a key regulator of Iron regulatory protein 1A, critical for maintaining cellular iron homeostasis^41^.

### Analysis of BRGC samples

We identified a total of 88 DEGs in the BRGC samples, which showed little overlap with WB and gut samples (Fig. 1D) and could be divided into three clusters (Supplementary Fig. S2). Cluster #1 genes showed strong upregulation in response to iron and this cluster was significantly enriched for seven heat shock protein genes (Supplementary Fig. S2A, B, Table 2). PG-specific RNAi targeting these heat shock genes revealed moderate developmental delays that could be rescued by iron supplementation, and in the case of Hsp70Bbb, high lethality (Supplementary Fig. S3A). A plausible link between iron and heat-shock proteins is provided by Hsp70, a known mediator of Fe-S cluster transfer to client proteins^63–65^, which physically interacts with Hsp22 as shown in this study (Supplementary Fig. S3B). Co-immunoprecipitation followed by MS also revealed that Hsp22 interacted physically with RSeFP, a mitochondrial Rieske iron-sulfur protein, mitochondrial Aconitase, and Fer1HCH, strongly suggesting that Hsp22 plays a role in iron metabolism (Supplementary Fig. S3B, C, Supplementary Data 5). Five of the heat shock genes showed strong transcript enrichment (Supplementary Fig. S3D) in the ring gland, consistent with the notion that the PG is iron-rich^25,41^.

Cluster #2 represented genes with moderate downregulation in response to iron. Cluster #3 was clearly distinct, and represented the biggest cohort. Remarkably, this cluster of 58 genes was characterized by a surge in expression at the 16-h time point under iron-deprived conditions. This surge was absent under iron-rich conditions, raising the possibility that this gene cohort is linked to a checkpoint that assesses iron stores. Intriguingly, the critical weight checkpoint^66^, by which larvae assess whether they have sufficient nutrients to survive metamorphosis, is normally just prior to this surge (0–12 h after the L2/L3 molt), suggesting that the induction of cluster #3 genes is caused by a failure to fulfill the critical weight checkpoint. Cluster #3 was enriched for 23 genes encoding proteins with a chitin-binding domain, but only Mco4 had an obvious link to iron, as it is orthologous to yeast Fet3p, a multi-copper oxidase involved in high-affinity iron import^67^. Like all cluster #3 genes, *Mco4* showed significant upregulation upon iron depletion, and a detailed functional analysis of Mco4 will be presented below.

### RNAi against candidate genes revealed iron storage defects

**Fig. 3:**
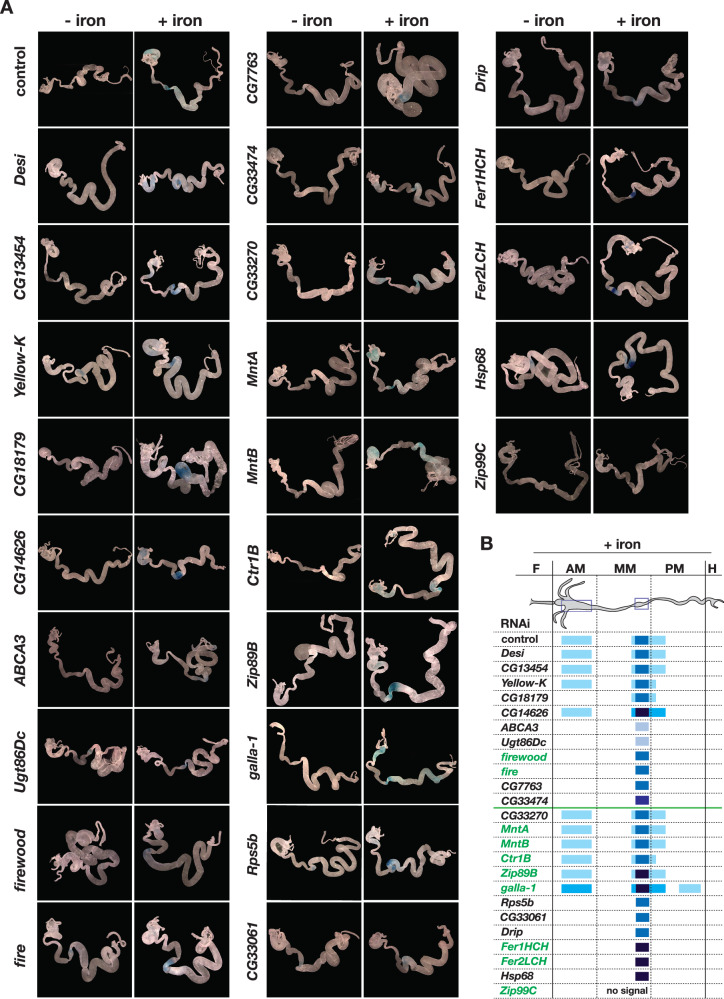
Prussian blue staining of larval guts from selected RNAi lines. **A** For each condition, 5–10 larval guts were stained with Prussian blue (ferrocyanide) and representative images are shown. Larvae were reared on iron-depleted (“− iron”, BPS-supplemented) or iron-supplemented (“+ iron”) diets. RNAi was driven by *NP3084-Gal4*, and *NP3084* > *w*^*1118*^ animals served as controls. Blue staining indicates both the spatial distribution and relative levels of ferric iron accumulation. **B** Schematic summarizing the Prussian blue staining results shown in panel (**A**). Genes above the green line show higher expression under iron-depleted conditions, while those below the line are more highly expressed under iron-supplemented conditions. Gene names in green indicate known links to iron biology. AM anterior midgut, MM middle midgut, PM posterior midgut. Source data are provided in the accompanying source data file.

We carried out two strategies to examine the validity of the RNA-Seq results. First, we selected candidate genes for all three sample types, and carried out qPCR. The results closely matched the RNA-Seq data (Supplementary Fig. S4). Next, we tested whether any of the genes identified in the gut cohorts was associated with phenotypes linked to iron metabolism. To this end, we analyzed 24 RNAi lines by staining dissected guts from *NP3084-GAL4* > DEG-RNAi with Prussian Blue to track the abundance and spatial distribution of gut-stored ferritin (Fig. 3A, B). The genes were selected from Table 1 and our DeSeq2 analysis (Supplementary Data 2) to include lowly expressed genes that appeared significant in the latter analysis, but were not found by the Arraystar software.

Larvae were reared on 120 μM BPS (iron depletion, no Prussian Blue staining) or 1 mM FAC (iron repletion). In controls, iron feeding resulted in a spatial expansion of the Prussian Blue stain compared to standard fly food, where the stain is limited to the iron region^68^. Excess iron absorption is normally met with the spatial expansion of ferritin gene expression in the gut, followed by iron deposition into ferritin. The spatial expansion of iron-loaded ferritin can be affected in multiple ways (e.g., faulty iron transport, disruption of ferritin gene expression, failure to deposit iron into ferritin, blocked iron export from the gut), resulting in both stronger and weaker stains. We observed different types of responses in the 24 RNAi lines tested: Importantly, half of the RNAi lines, including *firewood*- and *fire*-RNAi, showed a lack of spatial expansion of iron in the anterior midgut, suggesting their function was physiologically relevant in the iron homeostatic response. Five of the RNAi lines showed Prussian Blue patterns and intensities that were indistinguishable from controls (*Desi*, *CG13454*, *CG33270*, *MtnA*, *MtnB* in Fig. 3A, B). RNAi targeting *Zip99C* (aka Zip13), abolished Prussian Blue staining altogether, consistent with its role in loading iron into ferritin^52^. A strong reduction in the Prussian Blue staining in the iron region was also observed when *ABCA* or *Ugt86Dc* or *Drip* were targeted. By contrast, six lines displayed a stronger stain in and around the iron region, of which *Zip89B*- and *CG14626*-RNAi showed the additional expansion in the anterior midgut, whereas *Fer1HCH-, Fer2LCH-, Hsp68- and CG18179* were defective in the latter response (Fig. 3A, B). Finally, RNAi targeting *galla-1* showed further expansion of the Prussian Blue staining into the posterior midgut, albeit at reduced strength, reminiscent of observations of iron accumulation in mutants of iron-sulfur cluster assembly factors^69^. Interestingly, six of the genes tested here were part of gut cluster #6 (*CG7763*, *Drip*, *galla-1*, *Zip99C*, *Zip89B*, *MtnB*), however, their corresponding Prussian Blue patterns fell into five classes. As such, coordinated regulation does not imply similar function, but rather participation is the same molecular response.

In summary, Prussian Blue staining is a sensitive tool to assess whether a given RNAi line interferes with storing excess iron in the gut, and allowed us to quickly corroborate that candidate genes from the RNA-Seq data are involved in the regulation of iron physiology.

### The *fire* gene complex and *firewood*: Two ferric reductases and a cytochrome b5 electron donor function in *Drosophila* iron absorption

**Fig. 4:**
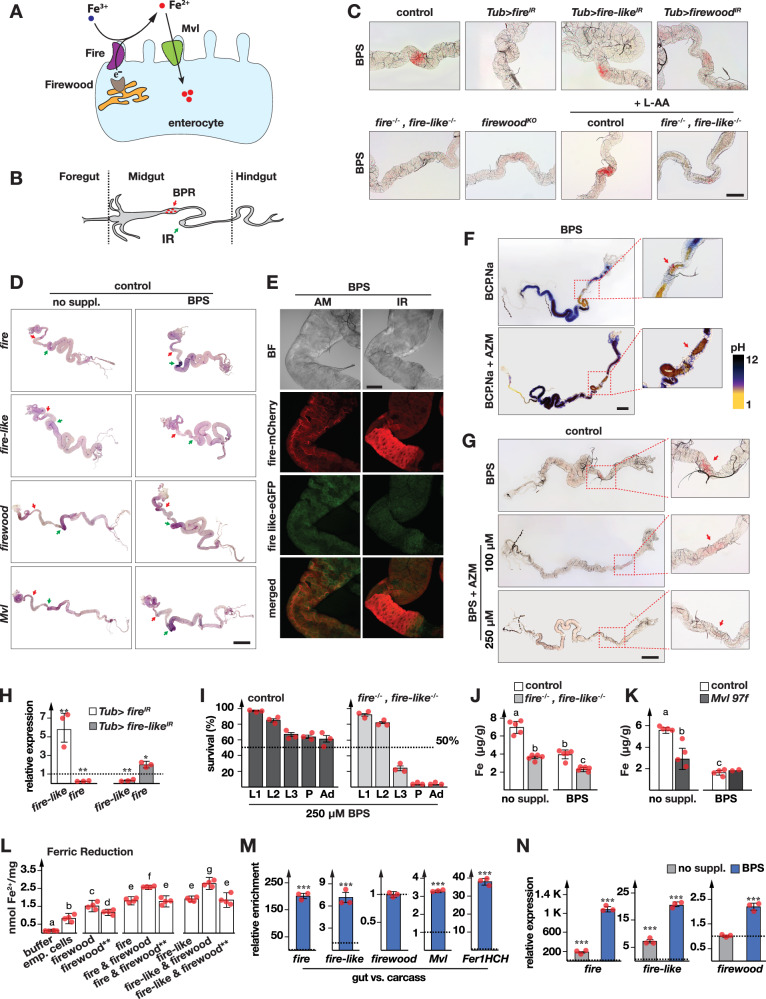
Ferric iron reduction mediated by Fire, Fire-like and Firewood. **A** Schematic of iron reduction and uptake mediated by Fire, Fire-like, Firewood and Malvolio (Mvl). **B** Diagram of the larval midgut showing the iron region (IR, green arrow) and BPS precipitation region (BPR, red arrow) in third instar larvae (L3). **C** Brightfield images of the BPR in midgut sections of control (*Tub* > *w*^*1118*^), *Tub>fire*^*IR*^, *Tub>fire-like*^*IR*^, *Tub>firewood*^*IR*^, *fire*^*-/-*^*, fire-like*^*-/-*^ (*fire* ^*2xKO*^) and *firewood* ^*KO*^ animals reared on BPS-supplemented media. Also shown: control (*w*^*1118*^) and *fire* ^*2xKO*^ larvae reared on BPS- and L-ascorbic acid (L-AA)-supplemented media. Scale bars: 80 µm. **D** In situ hybridization brightfield images of *fire*, *fire-like*, *firewood* and *Mvl* in control guts (*Tub* > *w*^*1118*^). Larvae were reared on normal (no suppl.) or BPS-containing diets, and dissected at 44 h after the L2/L3 molt. Red arrows: BPR. Green arrows: IR. Scale bars: 150 µm. **E** Fluorescent images of the anterior midgut (AM) and IR region in larvae expressing *fire-mCherry, fire-like-eGFP*, reared on a BPS-supplemented diet. Scale bar: 80 µm. BF Brightfield, Green: Fire-like-eGFP, Red: Fire-mCherry. **F** BPR and gut pH. Top: controls (*w*^*1118*^) fed with BPS and bromocresol purple sodium (BCP.Na). Bottom: as above, with acetazolamide (AZM) added. Scale bars: 150 µm. **G** Brightfield images of the BPR in control larvae (*w*^*1118*^) fed i) BPS, ii) BPS + 100 µM AZM or iii) BPS + 250 µM AZM. Scale bars: 150 µm. **H** qPCR analysis of *fire* and *fire-like* in L3 guts from *Tub>fire*^*IR*^ and *Tub>fire-like*^*IR*^ larvae. **I** Survival of control and *fire* ^*2xKO*^ on 250 µM BPS media. Dotted line: 50% pupariation. L1/L2/L3: 1^st^, 2^nd^ and 3^rd^ instar larvae. P: Pupae. Ad: Adults. Error bars: standard error (*n* = 3). **J**, **K** Total iron levels in L2 larvae (20 h after the L1/L2 molt) of control (*w*^*1118*^), *fire* ^*2xKO*^ and *Mvl* ^*97f*^ larvae reared on normal or 250 μM BPS-supplemented diets. Iron was quantified by ICP-OES and normalized to body weight. Error bars: standard deviation (*n* = 5 for **J**; *n* = 4 for K, except *Mvl* ^*97f*^ on BPS: *n* = 2). **L** Ferric reductase activity of lysis buffer (“buffer”) and cell lysates expressing empty vector (“emp. cells”), wild-type *firewood* (“*firewood”*), double-mutant *firewood* (“firewood^**^”), *fire* and *fire-like*, or combinations thereof. Fe^2+^ levels were measured by ferrozine assay. Error bars: Standard error (*n* = 4). Statistics in (**J**–**L**): ANOVA; different letters indicate significant differences (two-sided *p* < 0.05). **M** qPCR analysis of *fire*, *fire-like*, *firewood*, *Mvl* and *Fer1HCH* in gut vs. carcass (larval body without gut). **N** Expression of *fire*, *fire-like* and *firewood* on normal (no suppl.) or BPS-supplemented diets. **H**, **M**, **N** Data represent three biological replicates, each tested in triplicate. Error bars: 95% confidence intervals, center lines indicate means. Asterisks: significance by two-sided Student’s *t* test (***p < 0.01). Source data are provided in the accompanying source data file.

The RNA-Seq data showed that the most strongly affected genes in the gut samples were *fire*, *fire-like* and *firewood* (Table 1), which, together with *Malvolio*, riboflavin transporter *Rift*, acyl transferase *CG5892*, and peptidases *CG18179* and *CG9416* formed a small cluster of genes that showed a rapid (after 4 h) downregulation in response to iron starvation/re-feeding (Fig. 2A, Supplementary Data 4). Malvolio/DMT1 transports Fe^2+^, which is produced from dietary Fe^3+^ via a reduction reaction. Since Fire and Fire-like represent CYB561 family members, these enzymes are likely reducing Fe^3+^ to Fe^2+^ in the *Drosophila* gut, while their co-regulation with Firewood suggested the latter acts as an electron donor (Fig. 4A). To test this directly, we monitored the formation of BPS-Fe^2+^ precipitates in the gut. Since the chelation of BPS with Fe^2+^ results in a red precipitate, it can be used to detect high ferric reductase activity in the gut (Fig. 4B)^14^. Interestingly, BPS addition resulted in a narrow red stain anterior to the iron region (named “BPR” for “BPS precipitation region”, see Fig. 4B). This stain was abolished in *fire*-RNAi, and strongly reduced in *firewood*-RNAi guts, but remained unaffected in *fire-like*-RNAi larvae (Fig. 4C and Supplementary Fig. S6A), indicating that both Fire and Firewood contribute to dietary iron reduction. Consistent with this, the BPR was absent in both the *fire* ^*2xKO*^ double null mutant (*fire*^*−/−*^*, fire-like*^*−/−*^, Fig. 4C and Supplementary Fig. S7A) and the *firewood* ^*KO*^ single mutant, which we generated by CRISPR/Cas9-mediated deletion of the *fire*/*fire-like* and *firewood* loci (Supplementary Figs. S5A, B, and S6A, B). In *fire* ^*2xKO*^ heterozygotes, however, the BPR was present, suggesting that the loss of BPS staining in homozygotes was indeed due to the absence of functional Fire and Fire-like proteins (Supplementary Fig. S7A).

We next asked whether the BPR aligned with the spatial expression of *fire* and *fire-like*. To our surprise, RNA in situ hybridization revealed that both transcripts were expressed anterior to the BPR, with a noticeable drop in expression in the BPR itself (Fig. 4D). *Fire* also showed significant expression in the iron region (“IR”, see Fig. 4B), which increased substantially in BPS-supplemented food, consistent with our finding that Prussian Blue expansion fails to occur in *fire*-RNAi animals (Figs. 3 and 4D). We further assessed the expression of the *firewood* and *Mvl* transcripts to examine whether they displayed similar spatial distribution in the gut (Fig. 4D). Both *firewood* and *Mvl* transcripts were detected in the anterior midgut and the IR, comparable to *fire* and *fire-like*. The results were highly consistent with the cluster analysis that placed these four genes in a single cluster (Supplementary Data 4).

To assess subcellular localization, we generated a double knock-in CRISPR line in which the *fire* and *fire-like* loci were replaced with N-terminally tagged *fire-mCherry* and *fire-like-eGFP* alleles (Supplementary Fig. S8A). In agreement with the RNA in situ data, both proteins were detected in the anterior midgut (upstream of the BPR), the iron region, and the posterior midgut (Fig. 4E, Supplementary Fig. S8B, D).

Under iron-depleted conditions, Fire localized predominantly to the apical membrane of anterior and posterior midgut cells (Fig. 4E and Supplementary Fig. S8D), facing the gut lumen. In the iron region, however, the high expression levels made *fire* appear ubiquitously distributed throughout the cells (Fig. 4E and Supplementary Fig. S8E). Fire-like exhibited a similar but weaker expression pattern, with strongest localization in the anterior midgut, reduced presence in the posterior midgut, and no detectable signal in the iron region (Fig. 4E, Supplementary Fig. S8B, D), consistent with the in situ hybridization results. We reasoned that the discrepancy between *fire/fire-like* expression and the BPR was rooted in pH differences in the gut, and that the BPS-Fe^2+^ complex precipitates only in low pH^70,71^. To test this, we monitored the pH in the gut, for which we fed animals with bromocresol purple sodium dye designed to visualize regions of different acidities, while maintaining the BPS stain in the red BPR region. This approach revealed that the BPR is located in a small acidic region just downstream of the end of the neutral anterior midgut segment (Fig. 4F), raising the idea that the stain detected by BPS had precipitated locally after transitioning from a neutral to an acidic section of the gut. If true, the reduction of ferric to ferrous iron likely occurs in the anterior midgut where we detected *fire* and *fire-like* expression, but does not become visible until precipitation in acidic conditions.

To test whether the BPS stain was indeed dependent on acidic environments, we fed flies acetazolamide, a known inhibitor of carbonic anhydrase, an enzyme that drives luminal acidification^72^. As expected, inhibiting acidic conditions in the larval gut also abolished the BPS stain (Fig. 4F, G), consistent with the notion that the BPS stains in the BPR are due to soluble BPS-Fe^2+^ complexes entering an acidic environment, upon which they precipitate and become visible. Finally, we asked whether we could re-establish the BPS stain in the BPR region in *fire* ^*2xKO*^ animals by adding ascorbate to the medium. The idea was that excess ascorbate would reduce dietary Fe^3+^ to Fe^2+^, thereby increasing the concentration of BPS-Fe^2+^ complexes entering the gut, which would then precipitate in the BPR. Indeed, adding ascorbate resulted in a detectable BPS stain even in the absence of both Fire and Fire-like (Fig. 4C and Supplementary Fig. S7A), confirming that the BPR does not overlap with the expression pattern of these genes, but instead marks a downstream region where a change in acidity triggers local precipitation of BPS-Fe^2+^ complexes.

Ubiquitous RNAi targeting *fire-* or *fire-like* had no effect on viability and morphology on regular fly food. Both genes are adjacent to each other, suggesting they are the result of a gene duplication, and may overlap in their functions. Indeed, when we knocked down *fire*, the *fire-like* gene was induced 5.5-fold, and RNAi against *fire-like* caused a 2-fold upregulation of *fire*, indicating compensatory regulation (Fig. 4H). The double-mutant *fire* ^*2xKO*^ displayed no apparent phenotypes on regular fly food and food supplemented with moderate BPS concentrations (120 μM), suggesting that the presence of reducing agents in the fly media (e.g., propionic acid, which is necessary to prevent bacterial growth) provide sufficient ferrous iron for survival. When *fire* ^*2xKO*^ animals were reared on a diet containing 250 μM BPS to chelate ferrous iron before gut entry, less than 5% reached the pupal or adult stage, compared to ~60% survival in controls (Fig. 4I). This severe reduction in survival correlated with a marked decrease in larval iron levels, measured by Inductively Coupled Plasma Optical Emission Spectrometry (ICP-OES). *fire* ^*2xKO*^ mutants contained ~55% less iron than controls, both on regular food and food supplemented with 250 μM BPS (Fig. 4J). This degree of iron depletion was comparable to that observed in *Mvl* ^*97f*^ mutants^73^ on regular food (~50% reduction) in iron, but unlike *fire* ^*2xKO*^, *Mvl* ^*97f*^ mutants reared on BPS media retained iron levels similar to *w*^*1118*^ controls (Fig. 4K).

To directly confirm the ferric reductase activity of Fire and Fire-like, we conducted an ex vivo colorimetric assay in S2 cells using ferrozine as a Fe^2+^-specific chelator^74,75^ (Fig. 4L). Expression of *fire* or *fire-like* in S2 cells resulted in ~2.5-fold increase in ferric reduction compared to cells transfected with the empty vector (Fig. 4L). To assess whether Firewood contributes to ferric reduction via electron transfer, we co-transfected S2 cells with either wild-type *firewood* (*firewood*^*WT*^) or a mutant version (firewood^**^), in which the conserved heme-binding histidine residues (H39 and H63) were mutated to alanine to disrupt electron transfer (Supplementary Fig. S6C, D), together with *fire* and *fire-like*. Ferric reductase activity of Fire and Fire-like increased by ~1.5-fold in the presence of Firewood^WT^, but this enhancement was absent when mutant Firewood^**^ was used (Fig. 4L).

A final aspect we examined was whether *fire* gene expression was gut-specific. To this end, we compared gut samples with all remaining tissues (“carcass”). Overall, *fire* transcripts showed 200-fold enrichment (Fig. 4M), whereas *fire-like* transcripts were 7-fold enriched in the gut. By contrast, *firewood* expression was comparable to carcass. For reference, *Malvolio* and *Fer1HCH* showed ~3-fold and 37-fold higher expression levels in the gut (Fig. 4M). On BPS-containing media, *fire* was further upregulated to ~1100-fold, *fire-like* to ~21-fold, whereas *firewood* roughly doubled its expression (Fig. 4N). We conclude that *fire* expression is gut-specific, and encodes the main gut ferric reductase in the fly. Two additional CYB561 genes are located near the *fire/fire-like* (FFL) locus, which raised the possibility that they work in a redundant fashion with *fire* and *fire-like*. One, *CG10337*, lies 18.5 kb upstream of the FFL locus (separated by two other genes), whereas *CG10165* lies 37.0 kb upstream of the FFL locus (separated by another five genes from *CG10337*). When we examined the transcript levels of *CG10337* and *CG10165* and the other four CYB561 family members, we observed ~5-fold, ~11-fold and 3.5-fold transcript enrichment in the gut for *CG10337, CG10165* and *nemy*, respectively (Supplementary Fig. S5C). The *CG8399* and *CG3592* CYB561 transcripts did not exhibit enrichment in the gut. While the close chromosomal vicinity of the four CYB561 genes could suggest overlapping functions in vivo, we did not observe a corresponding response to changes in dietary iron in the RNA-Seq data, with *CG10165* showing around 1.1-fold differences throughout the four time points, whereas *CG10377* registered 1.25- to 2.1-fold changes. Similarly, the remaining four CYB561 genes (*CG3592*, *nemy*, *CG1275* and *CG8399*) all showed changes lower than ~2-fold. This was validated via qPCR, which showed that iron starvation most strongly affected *fire* and *fire-like*, with little effect on the other members, apart from *CG10337*, which exhibited a 3.5-fold upregulation in the gut (Supplementary Fig. S5C). By contrast, *fire* and *fire-like* showed maximal fold changes of 78- and 52-fold (Table 1), indicating that the response to dietary changes in iron concentrations was selective for the *fire* and *fire-like* genes. This is supported by the *fire*-RNAi and *fire* ^*2xKO*^ deletion studies in conjunction with BPS feeding, which demonstrated that the formation of BPS/Fe^2+^ precipitates fail to occur when Fire function is impaired or absent. This strongly suggests that, apart from Fire and Fire-like, no other CYB561 proteins participate in reducing dietary ferric iron in the gut, despite the fact Nemy and CG1275 display higher sequence similarities to human DCYTB (Supplementary Fig. S5D, E).

### Mco4 functions in cellular iron import

**Fig. 5:**
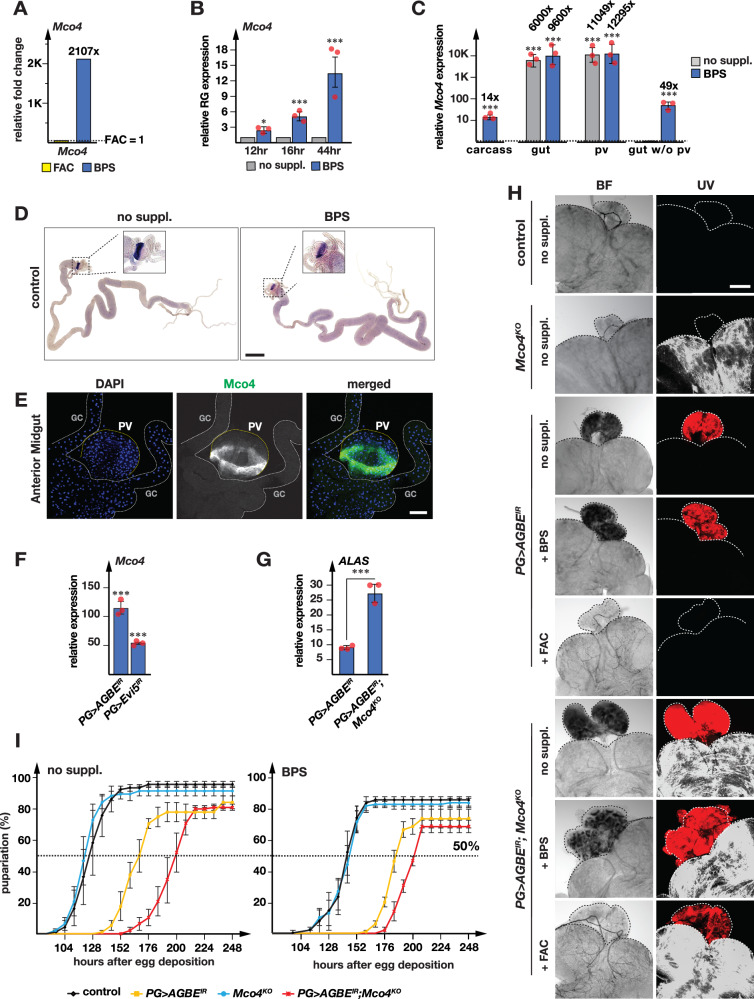
Mco4 is required for iron uptake into the prothoracic gland. **A** RNA-Seq analysis of *Mco4* expression in G6 BRGCs from larvae reared on BPS- or FAC-supplemented (iron-rich, normalized to 1) diets. **B** qPCR analysis of *Mco4* in ring glands (RG) of control (*w*^*1118*^) larvae at 12, 16 and 44 h after L2/L3 molt. Larvae were reared on normal (no suppl.) and on BPS- (iron-depleted) diets. **C** qPCR analysis of *Mco4* expression in the carcass, gut, proventriculus (PV), and gut without proventriculus (gut w/o PV) of control (*w*^*1118*^) larvae reared on normal (no suppl.) or BPS-supplemented diets. In (**B**, **C**), expression data are based on three biological replicates, each tested in triplicate. Error bars represent 95% confidence intervals, asterisks denote significance by two-sided Student’s *t* test (***p < 0.01). **D** Brightfield images of *Mco4* in situ hybridization in control (*w*^*1118*^) guts from larvae reared on normal or BPS-supplemented diets. Scale bars: 150 µm. **E** Immunodetection of Mco4 in the proventriculus (PV) of *Mco4*^*3xFLA*^ knock-in larvae. Blue: nuclei (DAPI), gray and green: Mco4 signal. Scale bars: 100 µm. **F** qPCR analysis of *Mco4* expression in ring glands (RG) from *PG* > *AGBE*^*IR*^ and *PG>Evi5*^*IR*^ larvae, normalized to *PG* > *w*^*1118*^ controls (set to 1). **G** qPCR analysis of *ALAS* expression in ring glands from *PG* > *AGBE*^*IR*^ and *PG* > *AGBE*^*IR*^*; Mco4*^*KO*^ larvae. **F**, **G** Data are from three biological replicates, each tested in triplicate. Error bars: 95% confidence intervals; asterisks: two-sided Student’s *t* test (***p < 0.01). **D**–**G** All larvae were dissected at 44 h after the L2/L3 molt. **H** Red autofluorescence in ring glands of i) control, ii) *Mco4*^*KO*^, iii) *PG* > *AGBE*^*IR*^ and iv) *PG* > *AGBE*^*IR*^*; Mco4*^*KO*^ larvae reared on normal (no suppl.), iron-rich (+FAC) and iron-depleted (+BPS) diets. UV ultraviolet, BF brightfield. Scale bars: 250 µm. **I** Developmental timing analysis of control, *Mco4*^*KO*^, *PG* > *AGBE*^*IR*^ and *PG* > *AGBE*^*IR*^*; Mco4*^*KO*^ larvae reared on normal (no suppl.) or BPS-supplemented diets. Y-axis: percentage pupariated. X-axis: hours after egg deposition. Dotted line marks 50% pupariation. Error bars: standard error (*n* = 3); center lines in (**B**, **C**, **F**, **G**, and **I**) represent means. Source data are provided in the accompanying source data file.

We initially identified *Mco4* due to its upregulation in response to BPS at the 16-h time point in the BRGC RNA-Seq data, which showed a ~2000-fold increase compared to the same time point under iron-replete conditions (Fig. 5A and Supplementary Fig. S2). In the 4-, 8- and 12-h time points, *Mco4* was nearly undetectable in BRGC samples (Supplementary Fig. S2), whereas absolute *Mco4* transcript at 16-h in the BRGC/BPS cohort were comparable to *Mco4* levels in the gut under all conditions. After one generation on 120 μM BPS media (representing moderate iron depletion), qPCR revealed 2-, 5- and 13-fold upregulation of *Mco4* in RG samples (Fig. 5B), confirming that *Mco4* is transcriptionally induced in response to iron deprivation. When we analyzed Mco4 transcript levels in the gut vs. carcass, we saw a 6000-fold enrichment in the gut, suggesting Mco4 exhibits gut-specific expression under iron-replete conditions (Fig. 5C). *Mco4* was not picked up in the gut RNA-Seq data, since *Mco4* expression levels were fairly robust at all time points, regardless of iron status, a finding we confirmed via qPCR, which showed a moderate ~1.6-fold upregulation of *Mco4* under BPS in whole gut samples (Fig. 5C). By contrast, we saw strong *Mco4* upregulation in the carcass in response to iron depletion, raising the possibility that the ring gland is not the only tissue that exhibits *Mco4* upregulation in the presence of BPS (Fig. 5C).

Next, we carried out in situ hybridization to analyze *Mco4* expression in the gut (Fig. 5D). Surprisingly, we found strong *Mco4* expression in the proventriculus (PV), where the spatial distribution formed a torus-like structure. Under iron depletion via BPS, *Mco4* expression increased moderately throughout the main sections of the midgut, but appeared stable in the PV (Fig. 5D). This was confirmed through qPCR analysis of *Mco4* expression in the PV and in guts where we had removed the PV. *Mco4* expression was virtually unchanged in the PV, but we observed a nearly 50-fold induction in the rest of the gut (Fig. 5C). We then generated a 3x-Flag-tagged *Mco4* knock-in via CRISPR/Cas9, which allowed us to confirm the torus-like expression pattern in the PV (Fig. 5E).

Given that *Mco4* is orthologous to yeast *Fet3*, we wondered whether Mco4 was part of a high-affinity iron import complex in *Drosophila*, which would represent the first report of such a system in an animal. Since we saw significant upregulation of *Mco4* in the ring gland in response to iron depletion, we worked first in this tissue and examined whether Mco4 would be also induced if we disrupted iron homeostasis by genetic means. To this end, we analyzed *Mco4* expression in genetic backgrounds where we disrupted either *AGBE* or *Evi5* in a PG-specific manner, which disrupted iron homeostasis and iron transport respectively^25,41^. Remarkably, we observed a ~100-fold induction of *Mco4* in *PG* > *AGBE-*RNAi animals, and a 50-fold induction in *PG>Evi5-*RNAi samples (Fig. 5F). This indicated that *Mco4* was not expressed under iron-replete conditions, but was strongly induced once iron became scarce, either by nutritional or genetic means.

Given the strong transcriptional induction of *Mco4* in both *AGBE-* and *Evi5*-RNAi lines, we wondered whether removing Mco4 in either of these backgrounds would exacerbate iron phenotypes in the PG. To this end, we generated an *Mco4* null allele by replacing the endogenous gene with a *3xP3-DsRed* (*Mco4*^*KO*^) reporter, which causes red fluorescence in the brain, but not the ring gland. *PG* > *AGBE-*RNAi animals have impaired cellular iron homeostasis, which causes a disruption of heme biosynthesis in the PG^41^. Disrupting heme production is characterized by the upregulation of *Alas*, which encodes the rate-limiting enzyme in heme biosynthesis, as well as heme precursor accumulation, which causes red autofluorescence in the affected tissue. Consistent with our earlier findings, *Alas* was ~8-fold upregulated in *PG* > *AGBE-*RNAi ring glands. Remarkably, *PG* > *AGBE-*RNAi; *Mco4*^*KO*^ animals displayed much stronger *ALAS* upregulation (>25-fold, Fig. 5G), demonstrating that removing *Mco4* in *PG* > *AGBE-*RNAi animals aggravated the impairment of heme biosynthesis due to lower iron uptake. To corroborate the *Alas* results, we next examined autofluorescence levels in these animals.

*PG* > *AGBE-*RNAi ring glands show strong red autofluorescence due to the disruption of iron homeostasis in this tissue. By contrast, ring glands isolated from *Mco4*^*KO*^ mutants display no autofluorescence (Fig. 5H). The red autofluorescence seen in *PG* > *AGBE-*RNAi ring glands can be completely rescued by supplementing fly media with iron (Fig. 5H). Importantly, this rescue requires Mco4, since *PG* > *AGBE-*RNAi; *Mco4* animals still exhibit strong red autofluorescence despite iron supplementation (Fig. 5H). These genetic data suggest that Mco4 contributes to maintaining cellular iron availability in the PG and are consistent with a role in facilitating iron import. The contribution of Mco4 to *AGBE*-RNAi phenotypes can be also observed on the population level. With standard food or mild BPS concentrations (120 μM), larvae where we removed *Mco4* from an otherwise wild-type background showed the same growth curves as controls and were equally viable (Fig. 5I). By contrast, *PG* > *AGBE-*RNAi; *Mco4*^*KO*^ animals were developmentally delayed by ~30 h compared to *PG* > *AGBE-*RNAi larvae, indicating that the transcriptional upregulation of *Mco4* in *AGBE*-RNAi animals is functionally important and likely a compensatory measure to counter low iron concentrations in the PG.

Taken together, these data indicate that *Mco4* is transcriptionally upregulated in response to a drop in cellular iron availability, and that Mco4 is involved in cellular mechanisms that promote iron import into the PG.

### Mco4 is the Fet3p ortholog essential for iron uptake under iron-starvation

**Fig. 6:**
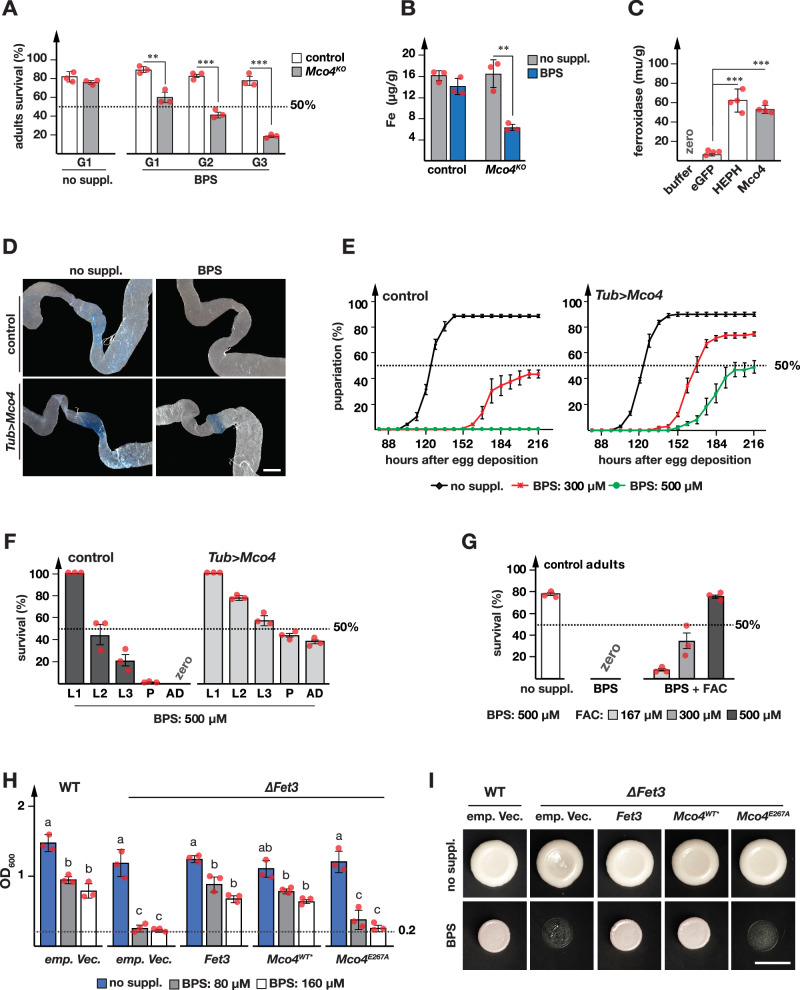
Mco4 functions as a ferroxidase in dietary iron absorption. **A** Adult survival of *Mco4*^*KO*^ and control (*w*^*1118*^) flies on normal (no suppl.) and iron-depleted diets (BPS). Flies were reared for three generations (G1-G3) on BPS-containing media. Dotted line: 50% pupariation. Error bars: standard error (*n* = 3). **B** Iron content of L3 control and *Mco4*^*KO*^ larvae measured by ICP-MS. Animals reared on normal (no suppl.) or BPS media, L3 larvae collected at 44 h after the L2/L3 molt. Y-axes: elemental iron (µg) per body weight (gram). Error bars: standard error (*n* = 3). **C** Ferroxidase assay of S2 cell extracts expressing empty vector (emp. vec.), human *hephaestin* (HEPH) or *Mco4*. Error bars: standard error (*n* = 4). Asterisks in (**A**–**C**) indicate significance (two-sided Student’s *t* test, ***p < 0.01). **D** Prussian blue staining of larval guts from *Tub* > *w*^*1118*^ and *Tub>Mco4-*cDNA larvae reared on normal (no suppl.) or BPS-supplemented diets. Blue color indicates ferric iron accumulation. Scale bars: 80 µm. **E** Developmental timing of control (*Tub* > *w*^*1118*^) and *Tub>Mco4-*cDNA larvae reared on normal diet (no suppl.) or BPS-supplemented food (300 µM, red; 500 µM, green). Y-axis: percentage pupariated. X-axis: hours after egg deposition. **F** Survival analysis of control and *Tub>Mco4-*cDNA larvae on 500 µM BPS-supplemented diet. Dotted line marks 50% pupariation. L1/L2/L3: 1^st^, 2^nd^, 3^rd^ instar larvae, P pupae, AD adults. **G** Survival of adult controls (*w*^*1118*^) on i) normal (no suppl.), ii) 500 µM BPS, iii-v) 500 µM BPS plus 160 µM, 300 µM and 500 µM FAC diets. Dotted line denotes 50% pupariation. **H** OD_600_ measurements of wild-type and ∆Fet3 yeast transformed with plasmids expressing *Mco4* cDNA, *Fet3* cDNA, or empty vector (emp. vector). Plasmids expressing *Fet3* or *Mco4* also co-expressed *Ftr1*. Cells were grown in synthetic drop-out medium containing zero (no suppl.), 80 and  160 µM BPS. Statistics: ANOVA; different letters denote significant differences (two-sided *p*-value < 0.05). **E**–**H** Error bars: standard error (*n* = 3); center lines represent means. **I** Growth of transformed yeast colonies on control (no suppl.) and BPS-supplemented (80 µM) media. Scale bars: 5 mm. Source data are provided in the accompanying source data file.

Under moderate iron depletion (160 μM BPS), we observed that *Mco4*^*KO*^ mutants displayed only 20% survival in the third generation compared to 80% in controls (Fig. 6A). The inability to survive on moderately iron-depleted media suggested that Mco4 played a role in the absorption of dietary iron in the gut. To test this idea, we determined iron concentrations in whole larvae using Inductively Coupled Plasma Mass Spectrometry (ICP-MS), which demonstrated that *Mco4*^*KO*^ mutants had comparable iron levels to controls when reared on regular fly food, but had ~60% less iron when reared on BPS-supplemented food (Fig. 6B). Thus, *Mco4* is required for iron absorption when bioavailability is low.

Multicopper oxidases can function as laccases, ascorbate oxidases and ferroxidases^76^. To test whether Mco4 had ferroxidase activity, we transfected Schneider 2 (S2) cells with plasmids encoding Hephaestin, a known human ferroxidase, or Mco4, and measured cell extracts in a ferroxidase enzyme assay. Both Hephaestin and Mco4 had comparable ferroxidase activity in this assay (Fig. 6C), consistent with the idea that Mco4 acts as a ferroxidase like its yeast counterpart Fet3p. Next, we asked whether overexpression of Mco4 was sufficient to increase iron concentrations in animals. While total iron was unaffected, we noticed that the iron region of *Mco4*-overexpressing animals showed a stronger Prussian Blue stain, indicative of higher levels of locally stored iron in the form of ferritin (Fig. 6D). Remarkably, in the presence of BPS, which normally results in the absence of a Prussian Blue stain in the iron region (IR), *Mco4* overexpression retained ferritin in the IR, indicating that *Mco4* overexpression increases stored iron in the IR, possibly due to higher absorption rates from the diet. Next, we examined whether this increase in stored iron would allow *Mco4*-overexpressing animals to survive otherwise lethal concentrations of BPS. This is indeed the case. When we reared control or *tub>Mco4*-cDNA animals on 500 μM BPS, controls were unable to complete larval development and never formed pupae. By contrast, *tub>Mco4-*cDNA animals not only formed pupae (Fig. 6E), but 40% of the population reached adulthood (Fig. 6F). To ensure that the lethality seen at these concentrations was solely due to the iron-chelating property of BPS and not due to non-specific effects of the compound, we repeated the experiment with control animals, but also tested a condition where we added simultaneously BPS and Ferric Ammonium Citrate (FAC), which showed that adding back iron completely rescued the lethality seen with BPS (Fig. 6G). Taken together, these data demonstrate that *Mco4* overexpression is sufficient to overcome iron starvation conditions that are lethal for wild-type, consistent with the idea that Mco4 is part of an iron import system that operates even in the presence of high iron chelator concentrations, suggesting a high-affinity mechanism.

Finally, we tested whether *Drosophila Mco4* could functionally replace yeast *Fet3*. For this experiment, we transfected *Fet3* mutants with empty vector, or plasmids harboring cDNAs for *Fet3* or *Mco4*, and measured growth under normal and iron-depleted conditions. Remarkably, transfection with either *Fet3* or *Mco4* resulted in a comparable rescue of the *Fet3* mutations under iron starvation (Fig. 6H, I and Supplementary Fig. S6B). To investigate whether the rescue of *Fet3* mutants by Mco4 was dependent on the enzyme’s ability to bind iron, we generated an Mco4 variant with impaired iron-binding capacity (*Mco4*^*E267A*^) by targeting Glu267 in Mco4 (Supplementary Fig. S7), which corresponds to Glu185 in Fet3p. Glu185 has been shown to play a crucial role in Fe^2+^ binding in Fet3p, electron transfer coupling, or Fe(III) trafficking^77,78^. Transfection of *Fet3* mutants Mco4^E267A^ failed to rescue (Fig. 6H, I and Supplementary Fig. S6B), reinforcing the conclusion that Mco4 has ferroxidase activity, and demonstrating that Mco4 can functionally replace Fet3p as part of the yeast high-affinity iron import system.

## Discussion

In this study, we used multi-generational iron starvation to gently sensitize animals towards iron re-feeding, in order to maximize the transcriptional response to a sudden increase in dietary iron levels. Overall, this iron starvation/re-feeding strategy successfully identified known iron metabolism genes, including iron transporters *Malvolio*, *mitoferrin*, *Zip99C* (aka Zip13), ferritin subunits *Fer1HCH*, *Fer2LCH* and a range of metallothionein genes. Importantly, we identified and functionally characterized four genes with no prior roles in insect iron biology: *fire*, *fire-like*, *firewood* and *Mco4*. We demonstrated that these genes represent players that ensure iron absorption under different conditions, identifying a hitherto unknown high-affinity uptake system in animals and a protein electron donor for iron reduction in the DMT1-dependent (low-affinity) iron absorption machinery.

Several findings suggest that Mco4 acts in the gut as a component of a high-affinity iron importer: First, *Mco4* mutants had significantly reduced iron stores and survival when grown on BPS-supplemented media. Second, overexpression of *Mco4* resulted in resistance to extreme levels of BPS, evidenced by the perdurance of iron-loaded ferritin in the gut and the ability to survive under these conditions, whereas controls had no survivors. Third, *Mco4* was upregulated in the PG in response to dietary iron restriction as well as genetic disruption of iron homeostasis and transport. Removing *Mco4* in a *PG* > *AGBE-*RNAi background abolished the rescue via iron supplementation, demonstrating that the upregulation of *Mco4* is a compensatory measure to import iron directly into the PG. Given that *Mco4* mutants have normal iron levels when reared on regular fly food, we conclude that Mco4 is required for iron import under iron starvation, at least in some peripheral tissues such as the PG.

Our findings in *Drosophila* explain the viability and mild phenotypes of *Malvolio* mutants, and we note that a similar conundrum exists for vertebrate iron homeostasis, where gut-specific loss of DMT1 function led to anemia but not lethality^18,19,79–84^. Similarly, only two of the three mammalian ferroxidases (ceruloplasmin and hephaestin) have been functionally analyzed to date^76^, raising the possibility that zyklopen may function in a similar manner to Fet3p and Mco4. Future identification of a permease analogous to yeast Ftr1 will be critical to fully establish whether Mco4 operates as part of a high-affinity iron import system in flies. Such an accessory protein may not be transcriptionally regulated and thus would have escaped detection by our experimental approach.

Under iron-replete conditions, *Mco4* is strongly expressed in the gut. Closer inspection revealed that *Mco4* expression was strongest in the proventriculus (PV), whereas the expression in the midgut epithelium was generally low but strongly upregulated under iron-depleted conditions. The torus-like expression pattern in the PV appeared constitutive and did not respond substantially to iron treatment. Interestingly, the Mco4 torus corresponds to the PV5.5 cell cluster that was recently described in a high-resolution transcriptomics study of the *Drosophila* foregut^85^. In that study, *Mco4*, alongside *CG7567* and *CG5162*, were identified as the major and specific marker genes of this foregut cell type. Neither *CG7567* nor *CG5162* showed iron-dependent regulation in our RNA-Seq data, consistent with the finding that *Mco4* in the PV is not regulated by dietary iron. The role of the PV5.5 cell cluster is currently unclear and requires further investigation. Notably, the PV and the ring gland are connected by neurons, raising the possibility that the PV communicates nutritional status (e.g., iron levels in the diet) to the gland, which in turn may result in systemic hormone signals.

Three of the most strongly downregulated genes in response to shifting animals to a high-iron diet were *fire*, *fire-like* and *firewood*. These three genes are closely linked, as indicated by their predicted functions, significant fold changes, temporal profiles, and their clustering with *Malvolio*. Malvolio/DMT1 transports ferrous iron and other divalent metals into the enterocyte, necessitating the conversion of dietary ferric iron to ferrous iron. Fire and Fire-like represent two of the eight CYB561 proteins encoded by the *Drosophila* genome, however, our data suggest that Fire is the principal gut ferric reductase. CYB561 proteins comprise a family of integral membrane oxidoreductases characterized by two non-covalently bound heme moieties and six transmembrane domains^86,87^, and they transfer electrons across the membrane to extracellular substrates such as ferric iron or monodehydroascorbate (MDHA). In *Candida albicans*, two proteins, Frp1 and Frp2, function in cellular heme uptake and are related to ferric reductases^88^. Unlike the Fire and Fire-like proteins, however, Frp1/2 do not belong to the CYB561 family. We thus favor the notion that Fire and Fire-like proteins act as canonical ferric reductases. This hypothesis is supported by multiple lines of evidence. First, both Fire and Fire-like exhibited ferric reductase activity in an ex vivo assay. Second, in a wild-type background, the addition of BPS to the diet led to the formation of a distinct precipitate—indicative of BPS-Fe^2+^ complex formation—whereas this response was abolished when *fire* was disrupted. These findings, along with apical membrane localization, suggest that Fire, and to a lesser extent Fire-like, are responsible for generating ferrous iron in the gut, enabling its chelation by BPS and subsequent precipitation.

A fascinating question concerns the source of electrons used by Fire and Fire-like. A commonly accepted idea is that cytoplasmic ascorbate serves as the main electron source for CYB561 enzymes, although redox potential studies suggest that ascorbate cannot account fully for the reduction of CYB561 proteins^86^. In our study, Firewood emerged as a strong candidate for a protein-based electron donor. Firewood contains a cytochrome b5-like heme/steroid-binding domain (Supplementary Fig. S6E, Interpro #IPR001199), a feature shared with 15 other *Drosophila* genes, none of which showed significant differential expression in our RNA-Seq data. Firewood also shows homology to human CYB5b, a known mitochondrial electron carrier. Firewood was co-regulated with *fire* and *fire-like* in response to iron availability, and promoted their ferric reductase activity in ex vivo assays. Importantly, a mutant version of Firewood, in which heme binding—and thus electron donation—was abolished, failed to enhance the ferric reductase activity of Fire and Fire-like. Furthermore, both *firewood*^*KO*^ mutants and *firewood*-RNAi animals exhibited a marked reduction in the formation of BPS precipitates, indicating impaired ferric reductase function.

In all likelihood, there are additional genes with specific roles in metal and/or iron biology in our RNA-Seq data. For example, the gut cohort was enriched for genes encoding transmembrane transporters (Table 2), some of which have been implicated to directly act in metal detoxification. One such example is the ABC transporter CG10505, first identified in a study where *Drosophila* larvae were fed for 6 h on diets containing either cadmium, zinc or copper^35,89^. Another upregulated ABC transporter in our dataset, encoded by *rdog*, is consistent with an earlier report linking this gene to zinc detoxification^90^. In addition to the iron transporter Zip99C (Zip13), we also identified Zip89B and Zip48C, predicted to act in zinc transport, and the copper transporter Ctr1B, suggesting that there is crosstalk between responses to different metals. We also note the response of *galla-1*, predicted to encode a protein acting in cytosolic iron cluster assembly required for proper IRP-1A and IRP-1B function^41^, and also found to be induced by cadmium, zinc or copper in the Schaffner study. Finally, our Prussian Blue analysis revealed abnormal iron ferritin storage in 18 RNAi lines, which suggested reduced iron uptake/transport/storage in these animals.

To conclude, our study has functionally characterized four genes involved in *Drosophila* iron absorption, and has introduced two major concepts: First, that an enzyme-based electron donor (Firewood) delivers electrons for extracellular ferric iron reduction, challenging the notion that cytoplasmic ascorbate is the sole electron source for this process. Second, that Mco4, fly ortholog of yeast Fet3p, is a ferroxidase that acts in high-affinity iron uptake, providing the first example of such a system in animals. Given the evolutionary conservation of many iron metabolism components from flies to humans, these findings pave the way for future studies to explore these novel concepts in the context of human iron metabolism.

## Methods

### Fly stocks

All fly strains used in this study are listed in Supplementary Table S1. The *w*^*1118*^ (BL3605) stock was obtained from the Bloomington *Drosophila* stock center and was used for the RNA-sequencing studies in this paper. RNAi lines were obtained from the Vienna stock center and the Bloomington *Drosophila* stock center. We used the following Gal4 drivers: *phm22-Gal4* (a kind gift from Michael O’Connor’s lab) and *NP3084-Gal4* (Kyoto *Drosophila* stock center: #113094) to drive GAL4 in the PG or gut cells, respectively. Flies were reared on standard fly food (Nutri-fly, #66-113) at 25 °C and 70% humidity.

### Media preparation and supplementation

Nutri-fly food was prepared according to the guidelines provided by the Bloomington *Drosophila* stock center (Flystuff, #66-113). To alter iron levels we supplemented Nutri-fly food with 100–500 µM Bathophenanthrolinedisulfonic acid disodium salt hydrate (BPS, Sigma-Aldrich, #146617-1G) for iron depletion (BPS-food) or 1 mM ferric ammonium citrate (FAC, Sigma-Aldrich, #F5879-100G) for iron enrichment (FAC-food). FAC-supplemented food was stored in the dark, whereas BPS media were stored under ambient light.

### Tissue collection, RNA isolation and library generation for RNA sequencing

To prepare the RNA-Seq samples, *Drosophila w*^*1118*^ flies were reared for six generations on 120 μM BPS-supplemented (iron-depleted) media. Larvae of the 6^th^ generation were carefully staged at the L2/L3 molt and transferred to either 1 mM FAC (iron-rich) or iron-depleted media (120 μM BPS). Brain-ring gland complexes (BRGC), whole gut and whole larvae (WB) were isolated from larvae reared on either media and were then collected at 4, 8, 12, and 16 h after the L2/L3 molt. Whole larval samples were washed in 1X PBS and gut samples were dissected in 1X PBS and transferred to TRIzol^TM^ reagent (Invitrogen, #15596026). 20–25 gut and whole-body samples were used to extract RNA following the manufacturer’s directions^91^. For BRGC, we dissected 30 specimens in 1X PBS and isolated RNA with the RNeasy Mini kit (Qiagen, #74104). RNA concentrations were measured with the Qubit 2.0 (Thermo Fisher Scientific) and the integrity of all samples was verified by the Agilent 2100 Bioanalyzer. We prepared 48 individual libraries (two replicates per each sample) with the Ovation RNA-Seq Systems 1–16 for Model Organisms kit (NuGEN, #0350). The RNA input for cDNA synthesis of each sample was 40 ng, and all cDNAs were fragmented prior to library generation. Fragmentation was carried out with the Covaris S220-Series System sonicator using a duty cycle of 10%, intensity at 5, 200 cycles, 180 s, 6 °C and a sample volume of 120 μl. The cDNA fragments were then ligated with provided barcode adapters for Illumina sequencing. Libraries were amplified by PCR, and their quality was assessed via the Agilent 2100 Bioanalyzer using Agilent High Sensitivity DNA 1000 Kit (Agilent, #5067-4626).

### Quantitative real-time PCR (qPCR)

All samples were collected in triplicate, and RNA was extracted as described above. All primer sequences are shown in Supplementary Data 1. RNA samples (0.25−1 µg/reaction) were reverse transcribed using the “High-Capacity cDNA Reverse Transcription” kit (Applied Biosystems™ #4374967). Synthesized cDNAs were diluted 1:20 for 1 µg total RNA per each reaction, and SYBR green Luna® Universal qPCR master mix (NEB #M3003L) was used for qPCR analysis in a QuantStudio 6 Flex instrument (Applied Biosystems). The ∆∆CT method was used to normalize samples based on *rp49* expression. Statistical differences in expression were tested by the Student’s *t* test from three biological replicates, and error bars represent 95% confidence intervals.

### Prussian blue iron staining

To stain for ferric iron, whole gut samples from 40 to 44-h 3rd instar larvae of *NP3084-Gal4* > RNAi animals were dissected in 1X PBS and immersed in fixation buffer (1X PBST with 4% Formaldehyde) for 25 min. Following three washes with 1X PBS for 10 min each, tissues were then incubated in 1% PBST for 20 min. Samples were then treated with Prussian blue stain for 45 min in 1% K_4_Fe(CN)_6_, 1% HCl in the dark at room temperature. Samples washed three times with PBS and were mounted in anti-fading mounting medium (Abcam, #AB188804) and photographed with a LEICA DFC500 camera.

### In situ hybridization

In situ hybridization probes were based on cDNAs obtained from the DGRC, and included *Mco4* (DGRC, #RE57944), *fire* (DGRC, #IP07844), *fire-like* (DGRC, #RH01238), *firewood* (DGRC, #IP06242), and *Mvl* (DGRC, #LD24465). The T7 promoter (5’- TAATACGACTCACTATAGGG −3’) was added to suitable regions of the ORF using Q5 DNA polymerase PCR amplification. Amplified fragments were purified via HighPrep PCR beads (MAGBIO, #AC-60005) and used as templates for probe synthesis by the SP6/T7 DIG RNA Labeling Kit (Sigma, #11175025910) following the manufacturer’s instructions. Probes were purified via lithium chloride precipitation. Probe concentrations were determined with the QubitTM RNA assay (Invitrogen, #Q32852), and probe quality was assessed via agarose gel electrophoresis. Gut samples from 40-h L3 were dissected in 1X PBS and fixed in 4% paraformaldehyde. The in situ hybridization procedure was carried out as described previously^92^. All solutions and buffer were created by RNase free water (Sigma, #4502-1L) and experiments were carried out in a RNase free environment. In situ hybridization primers are presented in Supplementary Data 1.

### Larval gut pH assay

Acetazolamide (Sigma, #A6011-10g, final concentrations of 100 μM and 250 μM) and bromocresol purple sodium salt (Sigma, # 860891-5 g, final concentration 0.1% w/v) were added to Nutrifly medium prior to solidification^72^. 36-h L3 were fed for 4 h on media containing both the pH indicator and Acetazolamide, after which guts were dissected in 1X PBS buffer. Intestines were transferred to anti-fade mounting medium (Abcam, #AB188804), and all images were taken immediately with a Zeiss A1 brightfield microscope.

### X-ray fluorescence microscopy (XRF) synchrotron iron analysis

BRGCs were dissected from 40-h *w*^*1118*^ L3 larvae in 0.25 M sucrose. Dissected BRGCs were transferred onto Thermanox cover slips and air-dried (Thermo Fisher Scientific # 50949476). X-ray fluorescence microscopy analysis (XRF) was performed at the Stanford Synchrotron Radiation light source (SSRL) at the SLAC National Accelerator Laboratory (https://www6.slac.stanford.edu), to generate elemental iron maps. All images were analyzed by the SMAK Microprobe Analysis Toolkit^93^.

### Construction of CRISPR/Cas9 and transgenic lines

The *Mco4* knock-in (*3x-FLAG-Mco4*) and *Mco4* knockout (*Mco4*^*KO*^) lines were generated by CRISPR/Cas9 homology-directed repair to produce FLAG-tagged Mco4 under endogenous control and a null deletion of the gene, respectively. Suitable genomic regions were obtained from FlyBase to design gRNAs via the CRISPR Optimal Target finder (http://targetfinder.flycrispr.neuro.brown.edu). All gRNA target sites were sequenced-verified by Sanger sequencing for the corresponding genomic regions in the Vas.Cas9 injection line (Bloomington #51323). Fragments corresponding to gRNAs were cloned into the pCFD5 plasmid (Addgene, #73914). All donor template fragments were amplified from genomic DNA of the Vas.Cas9 line via PCR and cloned into the backbone of the pDsRed-attP (Addgene, #51019) vector. For the knock-in line, the endogenous *Mco4* allele was replaced with a version carrying a C-terminal 3x-FLAG tag inserted immediately upstream of the stop codon in exon 4. For the knockout line, the Mco4 coding region was replaced with a 3xP3-DsRed marker cassette.

To generate the *UAS-Mco4-3Myc* transgenic line, a full-length *Mco4* cDNA (DGRC, #RE57944) was obtained from the *Drosophila* Genomic Resource Center. The *Mco4* wild-type cDNA was cloned into the PhiC31 pBID-UASC-GRM plasmid (Addgene, #35203). For the *fire*, *fire-like* double knockout, a transgenic gRNA line (*fire, fire-like*-gRNA) was generated based on four gRNAs (two gRNAs per gene) via the CRISPR Optimal Target finder (http://targetfinder.flycrispr.neuro.brown.edu) and cloning of the gRNAs into the pCFD5 plasmid (Addgene, #73914). pCFD5 is a PhiC31 vector with *vermilion* as a selectable marker, which expresses gRNAs under control of the U6-3 RNA Pol III snRNA promoter. To simplify screening for insertions, we replaced *vermilion* with *mini-white*. The double-mutant *fire* ^*2xKO*^ line was generated by crossing *UAS-fire, fire-like*-gRNA with *Act-Cas9* (Bloomington #54590). Mutant alleles were sequence-verified. Final plasmids and lines were confirmed by sequencing.

The *fire-mCherry*, *fire-like-eGFP* double knock-in line was generated using CRISPR/Cas9-meditated homology-directed repair. Two gRNAs – one upstream of *fire* and one downstream of *fire-like* – were designed based on results obtained via the CRISPR Optimal Target Finder. Target sites were sequence-verified via Sanger sequencing of the corresponding genomic loci in the *Nos.Cas9* line (Bloomington #54591) and cloned into the pCFD5 plasmid (Addgene, #73914). Donor template fragments were PCR-amplified from the genomic DNA of the *Nos.Cas9* line and cloned into the pDsRed-attP vector (Addgene, #51019), aimed at replacing the endogenous alleles of *fire* and *fire-like* genes with N-terminally tagged mCherry and eGFP, respectively.

A similar CRISPR/Cas9 strategy was used to generate the *firewood*^*KO*^ line. Here, the endogenous *firewood* locus was replaced with a DsRed marker flanked by two loxP sites. Two gRNAs targeting the upstream and downstream regions of *firewood* were designed with the CRISPR Optimal Target Finder tool and sequence-verified in the *Nos.Cas9* line (Bloomington #54591). These were cloned into the pCFD5 plasmid (Addgene, #73914). Donor template fragments were PCR-amplified from *Nos.Cas9* genomic DNA and inserted into the pDsRed-attP vector (Addgene, #51019). All plasmid fragments were amplified using Q5 High-Fidelity DNA Polymerase (NEB, #M0491S) and joined with the Gibson Assembly Master Mix (NEB, #E2611). All cloning and sequencing primers are provided in Supplementary Data 1.

### Yeast complementation assay

We used yeast strains BY4741 (wild-type) and Y16192 (∆Fet3). Cells were cultured in YPD broth (Sigma, #Y1375), YPDA plates (Sigma, #Y1500), and Synthetic Drop Out medium without uracil (SD-URA, Sigma, #Y1501-20g). Wild-type *Fet3* and *FTR1* genes were amplified from genomic BY4741 DNA and a full-length *Mco4* cDNA was obtained from the DGRC (#RE57944). Signal peptide sequences were identified with SignalP 5.091. We replaced the Mco4 secretion peptide with the first 40 amino acids of the Fet3p protein (Mco4^WT*^). The Mco4 Glu267 to Ala (*Mco4*^*E267A*^) mutant cDNA was derived from the wild-type Mco4^WT*^ cDNA via PCR mutagenesis. The pESC-URA plasmid was used to construct pESC-URA-Mco4^WT*^-FTR1, pESC-URA-*Mco4*^*E267A*^-*FTR1*, and pESC-URA-*Fet3*^*WT*^*-FTR1*. We then introduced these constructs into the ∆Fet3 strain (Y16192) along with pESC-URA (negative control, empty vector) and the wild-type strain with pESC-URA (positive control, empty vector) using lithium acetate-mediated transformation^94^, selecting for transformants on the appropriate synthetic drop-out medium. The transformed strains were verified via PCR amplification screening.

The complementation assay was carried out in SD-URA (no supplement) and in SD-URA with 80 μM or 160 μM BPS (iron-depleted medium). Overnight cultures from SD-URA (no supplement) were diluted to *OD*_*600*_ = 0.2 with sterile distilled water and incubated at 30 °C for ~14 h in appropriate media. ODs were measured in a Genesys^TM^ 150 Spectrometer (Thermo Scientific). For the plate assay, 10 μL of overnight culture grown in SD-URA (no supplement, *OD*_*600*_ = 0.2) were inoculated on SD-URA and SD-URA/80 μM BPS plates. Growth phenotypes were evaluated after three days at 30 °C. All media contained 2% galactose. All yeast strains and plasmids were generously provided by Dr. David Stuart’s lab at the University of Alberta, Edmonton, Canada. Fragments used for cloning were amplified using Q5 High-Fidelity DNA Polymerase (NEB, #M0491S), and plasmids were constructed using the Gibson Assembly Master Mix (NEB, #E2611). Yeast cell PCR screening was performed using Taq-DNA polymerase (NEB, #B9004S). All cloning and sequencing primers are listed in Supplementary Data 1.

### Inductively coupled plasma mass spectrometry (ICP-MS) analysis

Control (*w*^*1118*^) and *Mco4* knockout mutant (*Mco4*^*KO*^) animals were raised on a normal (non-supplemented) diet as well as a diet supplemented with 120 μM BPS (iron-depleted). Next, twenty 40-h L3 larvae were rinsed with MilliQ water. To purge ingested food from the gut, animals were kept in 1X PBS buffer for 30 min on a shaker with gentle agitation. Larvae were washed 3 × 15 min in MilliQ water to purge PBS buffer from the gut. Next, samples were digested in 1 mL of metal-free Nitric Acid (Thermo Fisher Scientific, #A509P212) at 200 °C using the CEM MARS6 microwave digestion system. ICP-MS analysis was performed using an Agilent 7900 ICP-MS instrument. Sample digestion and ICP-MS analysis were conducted at The University of Alberta Biogeochemical Analytical Service Laboratory (BASL).

### Inductively coupled plasma optical emission spectrometry (ICP-OES)

Control (*w*^*1118*^) and *fire* ^*2xKO*^ mutant flies were reared on two diets: standard Nutri-Fly food (non-supplemented) and Nutri-Fly food supplemented with 250 μM BPS (causes depletion of ferrous iron). For *Mvl* ^*97f*^ animals, both control (*w*^*1118*^) and mutant flies were reared on a standard diet (12.5% w/v molasses, 10% w/v brewer’s yeast, 1.6% w/v agar, 0.3% w/v gelatin, 1% w/v propionic acid, and 74.6% w/v water), with or without 250 μM BPS. Approximately 100 larvae (20-h L2 stage) were rinsed with MilliQ water and incubated in 1X PBS for 30 min with gentle agitation to purge ingested food. This was followed by three additional wash steps in MilliQ water to remove residual PBS. Samples were then digested in 1 mL of metal-free nitric acid (Thermo Fisher Scientific, #A509P212) at 200 °C using the CEM MARS6 microwave digestion system (CEM Corporation). Elemental analysis was performed using a PerkinElmer Optima 8300 ICP-OES instrument. Sample digestion and analysis were conducted at the Cinvestav in Mexico City, Mexico.

### Protein ferroxidase activity assay

The ferroxidase assay was based on the ceruloplasmin activity kit (Abcam, #273296) to assess the ferroxidase activity of Mco4 and HEPH proteins in *Drosophila* S2 cells. Full-length cDNA of *Drosophila Mco4* (DGRC, #RE57944) and human *HEPH* (GenScript, #OHu12228) were cloned into the pAFW plasmid with a C-terminal 3x Flag tag sequence. S2 cells were transfected using the Calcium-Phosphate transfection kit (Thermo Fisher Scientific, #K278001), and protein concentrations were assessed on Western blots. Transfected cells were harvested and washed 5x with 1X PBS buffer. Total proteins were then extracted using non-denaturing NP40 lysis buffer (Thermo Fisher Scientific, #J60766.AK), and protein concentrations were measured using the QubitTM Protein assay (Invitrogen, #Q33212).

We used 10 μL of the protein samples as input for the ceruloplasmin activity kit and followed the manufacturer’s instructions. Samples were added to a 96-well flat-bottom UV-star plate (Sigma-Aldrich, # M3812-40EA), and the absorbance at 560 nm was measured via the VICTOR Nivo Multimode Microplate Reader. Parameters for standard and sample curves were determined using the formula:

Sample Activity = S_k_/Ss/V × 2 (mU/mL), where S_k_ is the absorbance at the end and beginning of the linear range (∆OD_560_) divided by the time ∆T representing end and beginning of the linear range in minutes). Ss denotes the slope of the standard curve (OD/nmol). V is the volume of the sample in ml, and 2 accounts for the dilution factor of the ammonium sulfate-precipitated samples.

### Immunostaining of gut samples

For Mco4 immunodetection we dissected gut samples in 1X PBS buffer from 40 to 44 h L3 *Mco4-3xFLAG* knock-in animals. Guts were fixed for 30 min in fixation buffer (1X PBST with 4% Formaldehyde) and washed 3 × 15 min with PBST (1X PBS with 0.1% Triton, Sigma, #T9284). Tissues were then blocked for 1 h in blocking solution (1X PBST with 5% Goat serum) and washed 3 × 15 min in PBST. Samples were incubated with the primary antibody solution (Rabbit anti-Flag from Cell signaling #14793S with 1:800 dilution in 1X PBST with 1% BSA) overnight at 4 °C on a rocking shaker. Samples were then rinsed 3× in 1X PBST followed by incubation with the secondary antibody for 1 h (Alexa Fluor 488, Abcam #ab150077 with 1:2000 dilution). Samples were rinsed 3× in 1X PBST and mounted in VECTASHIELD solution (Cell signaling #4083). All images were taken with a Nikon C2si Confocal Microscope.

### RNA-Seq data analysis

Sequencing services and raw FastQ data were provided by the Genome Quebec Innovation Center (http://www.genomequebec.com/en/innovation-center/). The quality of the FastQ files was evaluated with FastQC (version: v0.11.8)^95^. We then used HISAT2 to map the reads to the *Drosophila* reference genome (BDGP5 version)^96^. Next, SAMtool and HTSeq analyses were performed to create SAM files and quantifying read counts per gene, respectively^97,98^. The raw reads were then used for statistical analysis and to identify DEGs. We used Arraystar (version 7) and two well-known parametric R packages, Deseq2 and edgeR, to identify DEGs based on a rate-adjusted *p*-value < 0.05 for gene selection. We used MS Access to filter for significance, fold changes and cohort overlaps. Finally, we used R version 3.4.4 to run the Deseq2 and edgeR. All DEGs are presented in Supplementary Data 2. For term enrichment analysis, we used the Chi Square test to compare number of observed vs. expected genes. To identify genes linked iron or metal biology we assembled a reference list comprising 839 iron/metal genes (Supplementary Data 3). This list was compiled by filtering the fly transcriptome for all relevant gene function descriptors that referred to “iron” and other metal-related terms (for exact parameters see Supplementary Data 3), but we excluded the very large group of zinc finger transcription factors. Heatmaps were generated via ComplexHeatmap^99^ and circlize^100^ R packages. For DEGs clustering, we employed the K-means clustering algorithm with 1000 iterations and 100 runs to identify distinct expression profiles (clusters) in DEGs of BRGC, gut, and whole larval body samples. All clusters are presented in Supplementary Data 4.

### Mass spectrometry of transfected S2 cells

Mass spectrometry assay was carried out in *Drosophila* S2 cells as previously described^41^. All primers are presented in Supplementary Data 1. We used Schneider insect medium (Sigma, #RNBH8523) supplemented with 10% heat-inactivated fetal bovine serum and 1% streptomycin-penicillin to grow the S2 cells. Cells were cultured in 1 mM FAC or 120 µM BPS media. Plasmids carrying Hsp22 (DGRC, #LD36162) and Hsp70 (DGRC, #LP05203) cDNAs were obtained from the DGRC. Hsp22, Hsp70 and GFP ORFs were cloned into pAFW plasmid with a C-terminus 3× Flag tag sequence. All fragments were amplified via the Q5 High-Fidelity DNA Polymerase (NEB, #M0491S). Plasmids were constructed using the Gibson Assembly Master Mix (NEB, #E2611) and verified by sequencing. S2 cells were transfected with pAFW-Hsp22-3xFLAG, pAFW-Hsp70-3xFLAG, and pAFW-GFP-3xFLAG plasmids via the Calcium-Phosphate method (Thermo Fisher Scientific, #K278001). Cells were harvested after 48 h and washed three times with 1X PBS and lysed with lysis buffer (50 mM Tris-HCL, pH 7.4, 150 mM NaCl, 1 mM EDTA, 0.1% NP-40 and 1× proteinase K inhibitor). Proteins concentrations were measured via the QubitTM Protein assay (Invitrogen, #Q33212). To pull down proteins, we used 40 µl of M2 Flag beads (Sigma, #A2220). Beads were added to Chromotek columns (sct-50 spin) and incubated with cell lysates for 4 h at 4 °C on a rocking shaker following instructions of the manufacturer. Beads were then washed 2X with wash buffer #1 (25 mM Na-HEPES pH 7.5, 75 mM NaCl, 0.5 mM EDTA, 10% glycerol, 0.1% Triton X-100) followed by three washes with wash buffer #2 (25 mM Na-HEPES, pH 7.5, 75 mM NaCl, 0.5 mM EDTA, 10% glycerol). Pulled-down proteins were eluted by adding elution buffer to the beads, and incubation of columns at 95 °C for 5 min. Samples were separated on a 12% SDS gel and stained with Coomassie Brilliant Blue using standard protocol. SDS gel slices (containing pulled-down proteins) were cut for MALDI-TOF mass spectrometry (Alberta Proteomics and MS facility at the University of Alberta. The MS results are presented in Supplementary Data 5.

### Cloning, co-immunoprecipitation assay and Western blotting

Plasmids carrying cDNAs of Hsp22 (DGRC, #LD36162), Hsp70 (DGRC, #LP05203), mAcon1 (DGRC, #LD24561), and RFeSP (DGRC, #SD14047) were obtained from the *Drosophila* Genomic Resource Center (DGRC; https://dgrc.bio.indiana.edu/Home). The Hsp22 and Hsp70 cDNA were cloned into pAFW plasmid so that they were in frame with the C-terminal sequence encoding the 3xFLAG. The pAFW-Hsp22-3xFLAG and pAFW-Hsp70-3xFLAG were then used to transfect S2 cells followed by immunoprecipitation. For co-immunoprecipitation and protein-protein interaction assays, the C terminus of mAcon1, RFeSP, and Fer1HCH DNA fragments were each fused to 5× Myc-tags. The tagged cDNAs were then cloned upstream of Hsp22-3xFLAG into the pAc5-STABLE-neo vector, which allows for simultaneously expressing two cDNAs: Both tagged cDNAs in a single plasmid are separated with T2A viral peptide, and upon cell transfection, the T2A is cleaved and allows equal production of either protein^101^.

All cDNA fragments and plasmid backbones were PCR-amplified with Q5 High-Fidelity DNA Polymerase (New England Biolabs Inc. #M0491S). Amplified fragments were then fused to each other via the Gibson assembly master mix (New England Biolabs Inc. #E2611) according to the manufacturer’s instructions. For co-immunoprecipitation and protein-protein interaction studies, the T2A sequence was added to the N-terminus of Hsp22-3FLAG and cloned into pAc5-STABLE-neo (the EGFP and NeoR/KanR fragments were removed). Plasmids were digested with KpnI (Thermo Fisher Scientific, #FD0524) and EcoRV (Thermo Fisher Scientific, # FD0303) in order to generate a backbone for the Gibson reaction with the mAcon1-5xMyc, RFeSP-5xMyc, and Fer1HCH-5xMyc fragments. Plasmids were cloned into competent DH5α *E. coli* cells. Prior to S2 cell transfection, all final plasmid constructs were validated via Sanger sequencing. All cloning and sequencing primers are showed in Supplementary Data 1.

*Drosophila* S2 cells were transfected with pAc5-STABLE-neo plasmids encoding Hsp22-3xFLAG (bait) and either Fer1HCH-5xMyc, mAcon1-5xMyc, or RFeSP-5xMyc (prey). After transfection, cells were washed and lysed, and lysates were incubated with M2 anti-FLAG agarose beads. Beads were washed twice with wash buffer #1 (25 mM Na-HEPES, pH 7.5; 75 mM NaCl; 0.5 mM EDTA; 10% glycerol; 0.1% Triton X-100), followed by three washes with wash buffer #2 (identical to buffer #1 but lacking Triton X-100). Bound protein complexes were then eluted for downstream analysis. The co-immunoprecipitated proteins were analyzed via Western blotting on a 12% SDS gel. To detect Flag-tagged and Myc-tagged proteins, we used monoclonal mouse anti-FLAG-tag (Cell Signaling, #8146S, 1:2000) and monoclonal rabbit anti-Myc-tag antibodies (Cell Signaling #2278S, 1:2000), respectively. Next, blots were incubated with goat anti-mouse IgG H&L HRP (Abcam #97023) and goat anti-rabbit IgG H&L HRP secondary antibodies (Abcam, #ab97051, 1:10,000. All blots were developed with the Amersham ECL Prime Western Blotting Detection reagent (Sigma, #GERPN2232) followed by scanning with the Bio-Rad ChemidocTM MP imaging system.

### Ex vivo ferric reductase assay in *Drosophila* S2 cells

*Drosophila* S2 cells were used to assess ferric reductase activity in an ex vivo assay. Cells were cultured in Schneider’s insect medium (Sigma, #RNBH8523) supplemented with 10% heat-inactivated fetal bovine serum and 1% Streptomycin-penicillin. Full-length *fire* (DGRC, # IP07844), *fire-like* (DGRC, # RH01238) and *firewood* (DGRC, #IP06242) cDNAs were cloned into the pAFW plasmid with a C-terminal 3xFLAG. A mutant *firewood* variant (designated firewood^**^), in which the conserved heme-binding residues His39 and His63 were mutated to alanine, was generated by site-directed mutagenesis of the wild-type *firewood* cDNA. All plasmid fragments were PCR-amplified with Q5 High-Fidelity DNA Polymerase (NEB, #M0491S) and assembled via the Gibson assembly master mix (NEB, #E2611). All primer sequences are listed in Supplementary Data 1.

For the ferrozine assay, 5 ml cultures of S2 cells were transfected with pAFW-*fire*-3xFLAG, pAFW-*fire-like*-3xFLAG, pAFW-*firewood*-3xFLAG or empty pAFW vector using Lipofectamine™ 3000 Reagent (Thermo Fisher Scientific, #L3000001), according to the manufacturer’s instructions. After 72 h, cells were harvested and washed three times in 1X PBS. For co-transfection experiments, plasmids were mixed in a 1:1 ratio prior to transfection.

Proteins were extracted using NP-40 lysis buffer (Thermo Fisher Scientific, #J60766.AP) supplemented with protease inhibitors (Sigma, # 11836170001). Cells were vortexed for 30 s, and briefly centrifuged (2350 × *g*), for a total of five times. Cell debris was not removed. Protein concentrations were determined using the QubitTM Protein assay kit (Invitrogen, #Q33212). For each reaction, 50 µL of cell lysate, 50 µL of freshly prepared 1 mM ferric ammonium citrate (Sigma #F5879), and 50 µL of 10 mM ferrozine solution were combined in a 96-well flat-bottom microplate (Sigma-Aldrich, # M3812-40EA). Samples were incubated for 1 h at room temperature on a shaker, and absorbance at 562 nm was measured using a VICTOR Nivo Multimode Microplate Reader.

To generate a standard curve, ferrous standards (ammonium iron (II) sulfate, Sigma, #09719-50 G) were prepared at final concentrations of 0, 10, 20, 30, 40, and 50 µM. Each standard (50 µL) was mixed with 50 µL of 10 mM ferrozine solution, and absorbance was measured at 562 nm. Sample absorbance values were converted to Fe^2+^ concentrations using the linear equation from the standard curve (y = mx + c).

### Reporting summary

Further information on research design is available in the Nature Portfolio Reporting Summary linked to this article.

## Supplementary information

Supplementary Information

Peer Review file

Description of Additional Supplementary Files

Supplementary Data 1

Supplementary Data 2

Supplementary Data 3

Supplementary Data 4

Supplementary Data 5

Reporting Summary

## Source data

Source Data

## Data Availability

The source data underlying all figures are provided. RNA sequencing raw data related to Fig. 1D, Fig. 2, Supplementary Fig. S2, Table 1, Table 2, Supplementary Data 2, and Supplementary Data 4 have been deposited in the NCBI Sequence Read Archive (SRA) under accession number PRJNA1149758. Mass spectrometry proteomics raw data related to Supplementary Fig. S3, Supplementary Data 5, and Supplementary Tables 3 and 4 have been deposited with the ProteomeXchange Consortium via the PRIDE partner repository (under accession #PXD055043). All other data supporting the findings of this study are available from the corresponding author upon request. Source data are provided with this paper.

## References

[1] Sheftel, A. D., Mason, A. B. & Ponka, P. The long history of iron in the Universe and in health and disease. *Biochim. Biophys. Acta* **1820**, 161–187 (2012). 21856378 10.1016/j.bbagen.2011.08.002 PMC3258305

[2] Muckenthaler, M. U., Rivella, S., Hentze, M. W. & Galy, B. A red carpet for iron metabolism. *Cell* **168**, 344–361 (2017). 28129536 10.1016/j.cell.2016.12.034 PMC5706455

[3] Lill, R. Function and biogenesis of iron-sulphur proteins. *Nature* **460**, 831–838 (2009). 19675643 10.1038/nature08301

[4] Barupala, D. P., Dzul, S. P., Riggs-Gelasco, P. J. & Stemmler, T. L. Synthesis, delivery and regulation of eukaryotic heme and Fe-S cluster cofactors. *Arch. Biochem. Biophys.* **592**, 60–75 (2016). 26785297 10.1016/j.abb.2016.01.010 PMC4784227

[5] Caldas Nogueira, M. L., Pastore, A. J. & Davidson, V. L. Diversity of structures and functions of oxo-bridged non-heme diiron proteins. *Arch. Biochem. Biophys.* **705**, 108917 (2021). 33991497 10.1016/j.abb.2021.108917 PMC8165033

[6] Nichol, H., Law, J. H. & Winzerling, J. J. Iron metabolism in insects. *Annu. Rev. Entomol.* **47**, 535–559 (2002). 11729084 10.1146/annurev.ento.47.091201.145237

[7] Dunkov, B. & Georgieva, T. Insect iron binding proteins: insights from the genomes. *Insect Biochem. Mol. Biol.* **36**, 300–309 (2006). 16551544 10.1016/j.ibmb.2006.01.007

[8] Mandilaras, K., Pathmanathan, T. & Missirlis, F. Iron absorption in Drosophila melanogaster. *Nutrients* **5**, 1622–1647 (2013). 23686013 10.3390/nu5051622 PMC3708341

[9] Mackenzie, B. & Garrick, M. D. Iron Imports. II. Iron uptake at the apical membrane in the intestine. *Am. J. Physiol. Gastrointest. Liver Physiol.* **289**, G981–6 (2005). 16286504 10.1152/ajpgi.00363.2005

[10] Holst, J. D., Murphy, L. G., Gorman, M. J. & Ragan, E. J. Comparison of insect and human cytochrome b561 proteins: insights into candidate ferric reductases in insects. *PLoS One* **18**, e0291564 (2023). 38039324 10.1371/journal.pone.0291564 PMC10691727

[11] Gunshin, H. et al. Cybrd1 (duodenal cytochrome b) is not necessary for dietary iron absorption in mice. *Blood* **106**, 2879–2883 (2005). 15961514 10.1182/blood-2005-02-0716 PMC1895297

[12] Asard, H., Barbaro, R., Trost, P. & Bérczi, A. Cytochromes b561: ascorbate-mediated trans-membrane electron transport. *Antioxid. Redox Signal* **19**, 1026–1035 (2013). 23249217 10.1089/ars.2012.5065 PMC3763232

[13] Iliadi, K. G. et al. nemy encodes a cytochrome b561 that is required for Drosophila learning and memory. *Proc. Natl. Acad. Sci. USA* **105**, 19986–19991 (2008). 19064935 10.1073/pnas.0810698105 PMC2604986

[14] Hernández-Gallardo, A. K. et al. In situ detection of ferric reductase activity in the intestinal lumen of an insect. *J. Biol. Inorg. Chem.* **29**, 773–784 (2024). 39617837 10.1007/s00775-024-02080-y PMC11638316

[15] Bettedi, L., Aslam, M. F., Szular, J., Mandilaras, K. & Missirlis, F. Iron depletion in the intestines of Malvolio mutant flies does not occur in the absence of a multicopper oxidase. *J. Exp. Biol.* **214**, 971–978 (2011). 21346125 10.1242/jeb.051664

[16] Shawki, A., Knight, P. B., Maliken, B. D., Niespodzany, E. J. & Mackenzie, B. H(+)-coupled divalent metal-ion transporter-1: functional properties, physiological roles and therapeutics. *Curr. Top. Membr.* **70**, 169–214 (2012). 23177986 10.1016/B978-0-12-394316-3.00005-3 PMC7027397

[17] Yambire, K. F. et al. Impaired lysosomal acidification triggers iron deficiency and inflammation in vivo. *Elife* **8**, e51031 (2019). 31793879 10.7554/eLife.51031 PMC6917501

[18] Gunshin, H. et al. Slc11a2 is required for intestinal iron absorption and erythropoiesis but dispensable in placenta and liver. *J. Clin. Invest* **115**, 1258–1266 (2005). 15849611 10.1172/JCI24356 PMC1077176

[19] Shawki, A. et al. Intestinal DMT1 is critical for iron absorption in the mouse but is not required for the absorption of copper or manganese. *Am. J. Physiol. Gastrointest. Liver Physiol.* **309**, G635–47 (2015). 26294671 10.1152/ajpgi.00160.2015 PMC4609933

[20] Gorman, M. J. Iron Homeostasis in Insects. *Annu. Rev. Entomol.* **68**, 51–67 (2023). 36170642 10.1146/annurev-ento-040622-092836 PMC10829936

[21] Gkouvatsos, K., Papanikolaou, G. & Pantopoulos, K. Regulation of iron transport and the role of transferrin. *Biochim. Biophys. Acta* **1820**, 188–202 (2012). 22085723 10.1016/j.bbagen.2011.10.013

[22] Kawabata, H. Transferrin and transferrin receptors update. *Free Radic. Biol. Med.* **133**, 46–54 (2019). 29969719 10.1016/j.freeradbiomed.2018.06.037

[23] Lambe, T. et al. Identification of a Steap3 endosomal targeting motif essential for normal iron metabolism. *Blood J. Am. Soc. Hematol.* **113**, 1805–1808 (2009). 10.1182/blood-2007-11-120402 PMC2947353 18955558

[24] Ohgami, R. S. et al. Identification of a ferrireductase required for efficient transferrin-dependent iron uptake in erythroid cells. *Nat. Genet.* **37**, 1264–1269 (2005). 16227996 10.1038/ng1658 PMC2156108

[25] Soltani, S., Webb, S. M., Kroll, T. & King-Jones, K. Drosophila Evi5 is a critical regulator of intracellular iron transport via transferrin and ferritin interactions. *Nat. Commun.* **15**, 4045 (2024). 38744835 10.1038/s41467-024-48165-9 PMC11094094

[26] Cornelis, P. Iron uptake and metabolism in pseudomonads. *Appl. Microbiol. Biotechnol.* **86**, 1637–1645 (2010). 20352420 10.1007/s00253-010-2550-2

[27] Nozoye, T. et al. Phytosiderophore efflux transporters are crucial for iron acquisition in graminaceous plants. *J. Biol. Chem.* **286**, 5446–5454 (2011). 21156806 10.1074/jbc.M110.180026 PMC3037657

[28] Kobayashi, T., Nozoye, T. & Nishizawa, N. K. Iron transport and its regulation in plants. *Free Radic. Biol. Med.* **133**, 11–20 (2019). 30385345 10.1016/j.freeradbiomed.2018.10.439

[29] Vert, G. et al. IRT1, an Arabidopsis transporter essential for iron uptake from the soil and for plant growth. *Plant Cell* **14**, 1223–1233 (2002). 12084823 10.1105/tpc.001388 PMC150776

[30] Kosman, D. J. Redox cycling in iron uptake, efflux, and trafficking. *J. Biol. Chem.* **285**, 26729–26735 (2010). 20522542 10.1074/jbc.R110.113217 PMC2930670

[31] Singh, A. K., McIntyre, L. M. & Sherman, L. A. Microarray analysis of the genome-wide response to iron deficiency and iron reconstitution in the cyanobacterium Synechocystis sp. PCC 6803. *Plant Physiol.* **132**, 1825–1839 (2003). 12913140 10.1104/pp.103.024018 PMC181269

[32] Liu, Y., Popovich, Z. & Templeton, D. M. Global genomic approaches to the iron-regulated proteome. *Ann. Clin. Lab. Sci.* **35**, 230–239 (2005). 16081578

[33] Ducey, T. F., Carson, M. B., Orvis, J., Stintzi, A. P. & Dyer, D. W. Identification of the iron-responsive genes of Neisseria gonorrhoeae by microarray analysis in defined medium. *J. Bacteriol.* **187**, 4865–4874 (2005). 15995201 10.1128/JB.187.14.4865-4874.2005 PMC1169496

[34] van de Mortel, J. E. et al. Large expression differences in genes for iron and zinc homeostasis, stress response, and lignin biosynthesis distinguish roots of Arabidopsis thaliana and the related metal hyperaccumulator Thlaspi caerulescens. *Plant Physiol.* **142**, 1127–1147 (2006). 16998091 10.1104/pp.106.082073 PMC1630723

[35] Yepiskoposyan, H. et al. Transcriptome response to heavy metal stress in Drosophila reveals a new zinc transporter that confers resistance to zinc. *Nucleic Acids Res.* **34**, 4866–4877 (2006). 16973896 10.1093/nar/gkl606 PMC1635269

[36] Kulshreshtha, R. et al. A microRNA signature of hypoxia. *Mol. Cell Biol.* **27**, 1859–1867 (2007). 17194750 10.1128/MCB.01395-06 PMC1820461

[37] Flanagan, J. M. et al. Microarray analysis of liver gene expression in iron overloaded patients with sickle cell anemia and beta-thalassemia. *Am. J. Hematol.* **84**, 328–334 (2009). 19384939 10.1002/ajh.21407

[38] Johnstone, D. & Milward, E. A. Genome-wide microarray analysis of brain gene expression in mice on a short-term high iron diet. *Neurochem. Int.* **56**, 856–863 (2010). 20350576 10.1016/j.neuint.2010.03.015

[39] Zamboni, A. et al. Genome-wide microarray analysis of tomato roots showed defined responses to iron deficiency. *BMC Genomics* **13**, 101 (2012). 22433273 10.1186/1471-2164-13-101 PMC3368770

[40] Zhou, Y. et al. Applying microarray-based technique to study and analyze silkworm (Bombyx mori) transcriptomic response to long-term high iron diet. *Genomics* **111**, 1504–1513 (2019). 30391296 10.1016/j.ygeno.2018.10.005

[41] Huynh, N., Ou, Q., Cox, P., Lill, R. & King-Jones, K. Glycogen branching enzyme controls cellular iron homeostasis via Iron Regulatory Protein 1 and mitoNEET. *Nat. Commun.* **10**, 5463 (2019). 31784520 10.1038/s41467-019-13237-8 PMC6884552

[42] Colombani, J. et al. Antagonistic actions of ecdysone and insulins determine final size in Drosophila. *Science* **310**, 667–670 (2005). 16179433 10.1126/science.1119432

[43] Mirth, C., Truman, J. W. & Riddiford, L. M. The role of the prothoracic gland in determining critical weight for metamorphosis in Drosophila melanogaster. *Curr. Biol.* **15**, 1796–1807 (2005). 16182527 10.1016/j.cub.2005.09.017

[44] Mehta, A., Deshpande, A., Bettedi, L. & Missirlis, F. Ferritin accumulation under iron scarcity in Drosophila iron cells. *Biochimie* **91**, 1331–1334 (2009). 19465081 10.1016/j.biochi.2009.05.003

[45] Cheng, L. Y. et al. Anaplastic lymphoma kinase spares organ growth during nutrient restriction in Drosophila. *Cell* **146**, 435–447 (2011). 21816278 10.1016/j.cell.2011.06.040

[46] Nookaew, I. et al. A comprehensive comparison of RNA-Seq-based transcriptome analysis from reads to differential gene expression and cross-comparison with microarrays: a case study in Saccharomyces cerevisiae. *Nucleic Acids Res.* **40**, 10084–10097 (2012). 22965124 10.1093/nar/gks804 PMC3488244

[47] Robinson, M. D., McCarthy, D. J. & Smyth, G. K. edgeR: a Bioconductor package for differential expression analysis of digital gene expression data. *Bioinformatics* **26**, 139–140 (2010). 19910308 10.1093/bioinformatics/btp616 PMC2796818

[48] Love, M. I., Huber, W. & Anders, S. Moderated estimation of fold change and dispersion for RNA-seq data with DESeq2. *Genome Biol.* **15**, 550 (2014). 25516281 10.1186/s13059-014-0550-8 PMC4302049

[49] Murray, M. T., White, K. & Munro, H. N. Conservation of ferritin heavy subunit gene structure: implications for the regulation of ferritin gene expression. *Proc. Natl. Acad. Sci. USA* **84**, 7438–7442 (1987). 3478702 10.1073/pnas.84.21.7438 PMC299311

[50] Hamburger, A. E., West, A. P., Hamburger, Z. A., Hamburger, P. & Bjorkman, P. J. Crystal structure of a secreted insect ferritin reveals a symmetrical arrangement of heavy and light chains. *J. Mol. Biol.* **349**, 558–569 (2005). 15896348 10.1016/j.jmb.2005.03.074

[51] Wu, S., Yin, S. & Zhou, B. Molecular physiology of iron trafficking in Drosophila melanogaster. *Curr. Opin. Insect Sci.* **50**, 100888 (2022). 35158107 10.1016/j.cois.2022.100888

[52] Xiao, G., Wan, Z., Fan, Q., Tang, X. & Zhou, B. The metal transporter ZIP13 supplies iron into the secretory pathway in Drosophila melanogaster. *Elife* **3**, e03191 (2014). 25006035 10.7554/eLife.03191 PMC4130162

[53] Xiao, G. & Zhou, B. ZIP13: a study of Drosophila offers an alternative explanation for the corresponding human disease. *Front. Genet.* **8**, 234 (2017). 29445391 10.3389/fgene.2017.00234 PMC5797780

[54] Xu, J., Wan, Z. & Zhou, B. Drosophila ZIP13 is posttranslationally regulated by iron-mediated stabilization. *Biochim. Biophys. Acta Mol. Cell Res.* **1866**, 1487–1497 (2019). 31229649 10.1016/j.bbamcr.2019.06.009

[55] Richards, C. D., Warr, C. G. & Burke, R. A role for dZIP89B in Drosophila dietary zinc uptake reveals additional complexity in the zinc absorption process. *Int. J. Biochem. Cell Biol.* **69**, 11–19 (2015). 26545796 10.1016/j.biocel.2015.10.004

[56] Dechen, K., Richards, C. D., Lye, J. C., Hwang, J. E. & Burke, R. Compartmentalized zinc deficiency and toxicities caused by ZnT and Zip gene over expression result in specific phenotypes in Drosophila. *Int. J. Biochem. Cell Biol.* **60**, 23–33 (2015). 25562517 10.1016/j.biocel.2014.12.017

[57] Sousa, C. A., Hanselaer, S. & Soares, E. V. ABCC subfamily vacuolar transporters are involved in Pb (lead) detoxification in Saccharomyces cerevisiae. *Appl. Biochem. Biotechnol.* **175**, 65–74 (2015). 25240850 10.1007/s12010-014-1252-0

[58] Qiang, W., Huang, Y., Wan, Z. & Zhou, B. Metal-metal interaction mediates the iron induction of Drosophila MtnB. *Biochem. Biophys. Res. Commun.* **487**, 646–652 (2017). 28435068 10.1016/j.bbrc.2017.04.109

[59] Stehling, O. et al. Human CIA2A-FAM96A and CIA2B-FAM96B integrate iron homeostasis and maturation of different subsets of cytosolic-nuclear iron-sulfur proteins. *Cell Metab.* **18**, 187–198 (2013). 23891004 10.1016/j.cmet.2013.06.015 PMC3784990

[60] Maione, V., Cantini, F., Severi, M. & Banci, L. Investigating the role of the human CIA2A-CIAO1 complex in the maturation of aconitase. *Biochim. Biophys. Acta Gen. Subj.* **1862**, 1980–1987 (2018). 29842905 10.1016/j.bbagen.2018.05.019

[61] Vásquez-Procopio, J. et al. Intestinal response to dietary manganese depletion in Drosophila. *Metallomics* **12**, 218–240 (2020). 31799578 10.1039/c9mt00218a

[62] Metzendorf, C. & Lind, M. I. Drosophila mitoferrin is essential for male fertility: evidence for a role of mitochondrial iron metabolism during spermatogenesis. *BMC Dev. Biol.* **10**, 68 (2010). 20565922 10.1186/1471-213X-10-68 PMC2905335

[63] Dutkiewicz, R., Nowak, M., Craig, E. A. & Marszalek, J. Fe-S cluster Hsp70 chaperones: the ATPase cycle and protein interactions. *Methods Enzymol.* **595**, 161–184 (2017). 28882200 10.1016/bs.mie.2017.07.004 PMC6287258

[64] Dutkiewicz, R. & Nowak, M. Molecular chaperones involved in mitochondrial iron-sulfur protein biogenesis. *J. Biol. Inorg. Chem.* **23**, 569–579 (2018). 29124426 10.1007/s00775-017-1504-x PMC6006194

[65] Puglisi, R. & Pastore, A. The role of chaperones in iron–sulfur cluster biogenesis. *FEBS Lett.* **592**, 4011–4019 (2018). 30194723 10.1002/1873-3468.13245 PMC6506825

[66] Zeng, J., Huynh, N., Phelps, B. & King-Jones, K. Snail synchronizes endocycling in a TOR-dependent manner to coordinate entry and escape from endoreplication pausing during the Drosophila critical weight checkpoint. *PLoS Biol.* **18**, e3000609 (2020). 32097403 10.1371/journal.pbio.3000609 PMC7041797

[67] Singh, A., Severance, S., Kaur, N., Wiltsie, W. & Kosman, D. J. Assembly, activation, and trafficking of the Fet3p.Ftr1p high affinity iron permease complex in Saccharomyces cerevisiae. *J. Biol. Chem.* **281**, 13355–13364 (2006). 16522632 10.1074/jbc.M512042200

[68] Missirlis, F. et al. Homeostatic mechanisms for iron storage revealed by genetic manipulations and live imaging of Drosophila ferritin. *Genetics* **177**, 89–100 (2007). 17603097 10.1534/genetics.107.075150 PMC2013694

[69] Uhrigshardt, H., Rouault, T. A. & Missirlis, F. Insertion mutants in Drosophila melanogaster Hsc20 halt larval growth and lead to reduced iron-sulfur cluster enzyme activities and impaired iron homeostasis. *J. Biol. Inorg. Chem.* **18**, 441–449 (2013). 23444034 10.1007/s00775-013-0988-2 PMC3612401

[70] Derman, D. P. et al. A systematic evaluation of bathophenanthroline, ferrozine and ferene in an ICSH-based method for the measurement of serum iron. *Ann. Clin. Biochem.* **26**, 144–147 (1989). 2729856 10.1177/000456328902600209

[71] Martell, A. E. & Smith, R. M. *Critical stability constants* (Springer, 1974).

[72] Overend, G. et al. Molecular mechanism and functional significance of acid generation in the Drosophila midgut. *Sci. Rep.* **6**, 27242 (2016). 27250760 10.1038/srep27242 PMC4890030

[73] Rodrigues, V., Cheah, P. Y., Ray, K. & Chia, W. malvolio, the Drosophila homologue of mouse NRAMP-1 (Bcg), is expressed in macrophages and in the nervous system and is required for normal taste behaviour. * EMBO J.* **14**, 3007–3020 (1995). 7621816 10.1002/j.1460-2075.1995.tb07303.x PMC394361

[74] Missirlis, F. et al. Characterization of mitochondrial ferritin in Drosophila. *Proc. Natl. Acad. Sci.* **103**, 5893–5898 (2006). 16571656 10.1073/pnas.0601471103 PMC1458669

[75] Stookey, L. L. Ferrozine-a new spectrophotometric reagent for iron. *Anal. Chem.* **42**, 779–781 (1970).

[76] Helman, S. L. et al. The biology of mammalian multi-copper ferroxidases. *Biometals* **36**, 263–281 (2023). 35167013 10.1007/s10534-022-00370-z PMC9376197

[77] Lang, M., Braun, C. L., Kanost, M. R. & Gorman, M. J. Multicopper oxidase-1 is a ferroxidase essential for iron homeostasis in Drosophila melanogaster. *Proc. Natl. Acad. Sci. USA* **109**, 13337–13342 (2012). 22847425 10.1073/pnas.1208703109 PMC3421175

[78] Taylor, A. B., Stoj, C. S., Ziegler, L., Kosman, D. J. & Hart, P. J. The copper-iron connection in biology: structure of the metallo-oxidase Fet3p. *Proc. Natl. Acad. Sci. USA* **102**, 15459–15464 (2005). 16230618 10.1073/pnas.0506227102 PMC1257390

[79] Beaumont, C., Delaunay, J., Hetet, G. & Grandchamp, B. Two new human DMT1 gene mutations in a patient with microcytic anemia, low ferritinemia, and liver iron overload. *Blood* **107**, 4168–4170 (2006). 10.1182/blood-2005-10-4269 16439678

[80] Donovan, A. et al. The zebrafish mutant gene chardonnay (cdy) encodes divalent metal transporter 1 (DMT1). *Blood* **100**, 4655–4659 (2002). 12393445 10.1182/blood-2002-04-1169

[81] Fleming, M. D. et al. Microcytic anaemia mice have a mutation in Nramp2, a candidate iron transporter gene. *Nat. Genet.* **16**, 383–386 (1997). 9241278 10.1038/ng0897-383

[82] Fleming, M. D. et al. Nramp 2 is mutated in the anemic Belgrade (b) rat: evidence of a role for Nramp2 in endosomal iron transport. *Proc. Natl. Acad. Sci.* **95**, 1148–1153 (1998). 9448300 10.1073/pnas.95.3.1148 PMC18702

[83] Iolascon, A. et al. Microcytic anemia and hepatic iron overload in a child with compound heterozygous mutations in DMT1 (SCL11A2). *Blood* **107**, 349–354 (2006). 16160008 10.1182/blood-2005-06-2477

[84] Mims, M. P. et al. Identification of a human mutation of DMT1 in a patient with microcytic anemia and iron overload. *Blood* **105**, 1337–1342 (2005). 15459009 10.1182/blood-2004-07-2966

[85] Zhu, H., Ludington, W. B. & Spradling, A. C. Cellular and molecular organization of the Drosophila foregut. *Proc. Natl. Acad. Sci. USA* **121**, e2318760121 (2024). 38442150 10.1073/pnas.2318760121 PMC10945768

[86] Bérczi, A. & Zimányi, L. The trans-membrane cytochrome b561 proteins: structural information and biological function. *Curr. Protein Pept. Sci.* **15**, 745–760 (2014). 25163754 10.2174/1389203715666140828100351

[87] Ganasen, M. et al. Structural basis for promotion of duodenal iron absorption by enteric ferric reductase with ascorbate. *Commun. Biol.* **1**, 120 (2018). 30272000 10.1038/s42003-018-0121-8 PMC6123691

[88] Roy, U. et al. Ferric reductase-related proteins mediate fungal heme acquisition. *Elife* **11**, e80604 (2022). 36200752 10.7554/eLife.80604 PMC9635878

[89] Günther, V., Lindert, U. & Schaffner, W. The taste of heavy metals: gene regulation by MTF-1. *Biochim. Biophys. Acta* **1823**, 1416–1425 (2012). 22289350 10.1016/j.bbamcr.2012.01.005

[90] Mohr, S. E. et al. Zinc Detoxification: A Functional Genomics and Transcriptomics Analysis in Drosophila melanogaster Cultured Cells. G3 (Bethesda) **8**, 631–641 (2018). 10.1534/g3.117.300447 PMC5919732 29223976

[91] Denton, D. & Kumar, S. Analyzing the response of RNAi-treated Drosophila cells to death stimuli by quantitative real-time polymerase chain reaction. *Cold Spring Harb. Protoc.* **2015**, 666–670 (2015). 26134907 10.1101/pdb.prot086223

[92] Chung, H. et al. Characterization of Drosophila melanogaster cytochrome P450 genes. *Proc. Natl. Acad. Sci.* **106**, 5731–5736 (2009). 19289821 10.1073/pnas.0812141106 PMC2667016

[93] Webb, S. M. The MicroAnalysis Toolkit: X-ray fluorescence image processing software. *AIP Conf. Proc.* **1365**, 196–199 (2011).

[94] Gietz, R. D. & Woods, R. A. Transformation of yeast by lithium acetate/single-stranded carrier DNA/polyethylene glycol method. *Methods Enzymol.* **350**, 87–96 (2002). 12073338 10.1016/s0076-6879(02)50957-5

[95] de Sena Brandine, G. & Smith, A. D. Falco: high-speed FastQC emulation for quality control of sequencing data. *F1000Res* **8**, 1874 (2019). 33552473 10.12688/f1000research.21142.1 PMC7845152

[96] Kim, D., Langmead, B. & Salzberg, S. L. HISAT: a fast spliced aligner with low memory requirements. *Nat. Methods* **12**, 357–360 (2015). 25751142 10.1038/nmeth.3317 PMC4655817

[97] Li, H. et al. The Sequence Alignment/Map format and SAMtools. *Bioinformatics* **25**, 2078–2079 (2009). 19505943 10.1093/bioinformatics/btp352 PMC2723002

[98] Anders, S., Pyl, P. T. & Huber, W. HTSeq-a Python framework to work with high-throughput sequencing data. *Bioinformatics* **31**, 166–169 (2015). 25260700 10.1093/bioinformatics/btu638 PMC4287950

[99] Gu, Z., Eils, R. & Schlesner, M. Complex heatmaps reveal patterns and correlations in multidimensional genomic data. *Bioinformatics* **32**, 2847–2849 (2016). 27207943 10.1093/bioinformatics/btw313

[100] Gu, Z., Gu, L., Eils, R., Schlesner, M. & Brors, B. circlize Implements and enhances circular visualization in. *R. Bioinforma.* **30**, 2811–2812 (2014). 10.1093/bioinformatics/btu393 24930139

[101] Donnelly, M. L. L., Hughes, L. E. & Luke G*.* The ‘cleavage’activities of foot-and-mouth disease virus 2A site-directed mutants and naturally occurring ‘2A-like’sequences. *J. General* **82**, 1027–1041 (2001). 10.1099/0022-1317-82-5-1027 11297677

