# Supplementary material for: New molecular components of high and low affinity iron import systems in Drosophila
Source: Nat Commun. 2025 Jul 1;16:5662. doi: 10.1038/s41467-025-60758-6 (PMC12218971; doi:10.1038/s41467-025-60758-6)
Supplement: Supplementary file 1 — Supplementary Information [file 41467_2025_60758_MOESM1_ESM.pdf]

Supplementary Information

"New molecular components of high and low affinity iron import systems in *Drosophila*"

Soltani et al.

Supplementary Figures

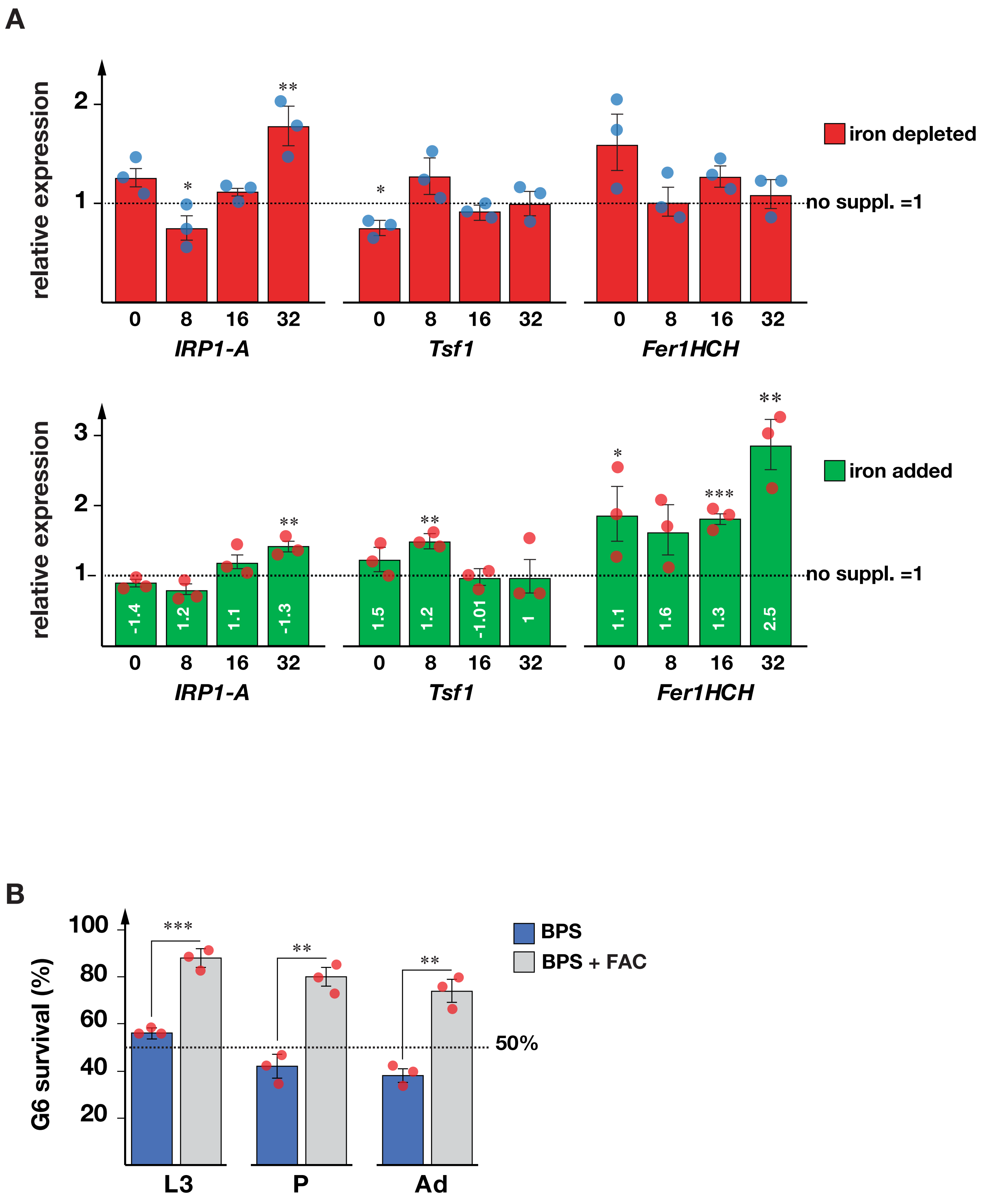

A
relative expression
2
1
**
*
*
iron depleted
no suppl. =1
0
8
16
32
IRP1-A
Tsf1
Fer1HCH
relative expression
3
2
1
**
**
**
*
***
iron added
no suppl. =1
-1.4
1.2
1.1
-1.3
1.5
1.2
-1.01
1
1.1
1.6
1.3
2.5
B
G6 survival (%)
100
80
60
40
20
***
**
**
BPS
BPS + FAC
50%
L3
P
Ad

Figure S1. Transcriptional responses due to short-term changes in iron concentrations

Time course analysis of transcript levels for *Iron-regulatory protein 1A* (*IRP1A*), *transferrin 1* (*Tsf1*) and *ferritin 1 heavy chain* (*Fer1HCH*) in whole larvae reared under iron-depleted (BPS-supplemented; red) or iron-supplemented (addition of FAC; green) conditions. Relative fold changes were normalized to 1 (gray box and dotted line), which represents the expression of a given gene at the same time point on standard medium. The X-axes indicate the time of sample collection (0, 8, 16, and 32 hours after L2/L3 molt). The numbers in the green boxes show the fold difference relative to the same time point under iron-depleted conditions. Expression data are based on three biological replicates, each tested in triplicate. Error bars represent 95% confidence intervals. **B)** Survival analysis of control (w^{1118}) flies reared for five generations (G5) on the iron-depleted medium (BPS-supplemented, blue bars) after being transferred to a diet supplemented with iron (white bars, 1 mM FAC). Dotted line: 50% pupariation. L3: 3rd instar larvae, P: Pupae, Ad: Adults. Error bars indicate standard deviation from three biological replicates; means are centered. Asterisks in (**A and B**) denote significance level based on a two-sided Student's *t*-test (*$p < 0.05$ and ***$p < 0.01$). Source data are available in the accompanying source data file.

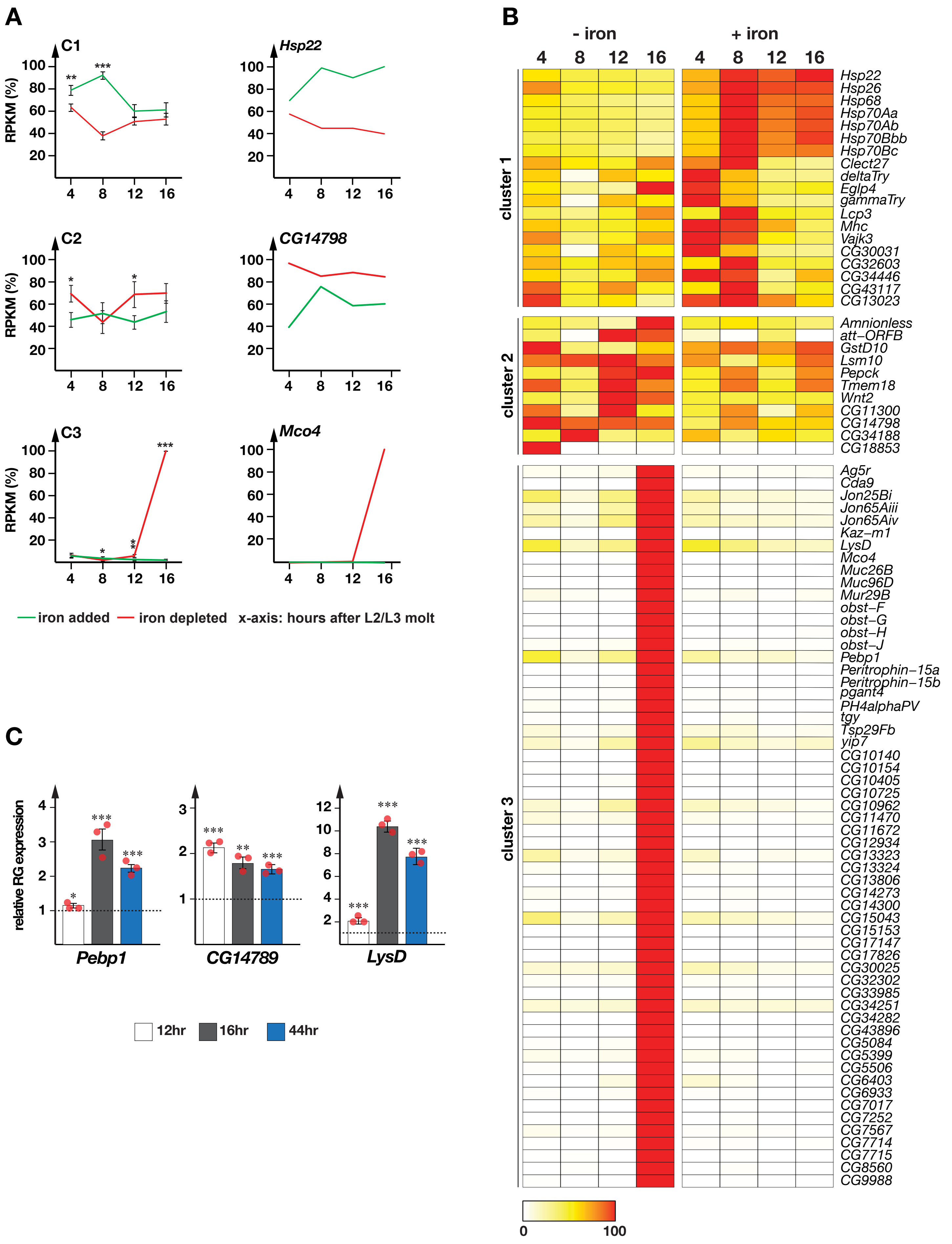

A
C1
Hsp22
C2
CG14798
C3
Mco4
RPKM (%)
iron added
iron depleted
x-axis: hours after L2/L3 molt
B
- iron
+ iron
cluster 1
cluster 2
cluster 3
Hsp22
Hsp26
Hsp68
Hsp70Aa
Hsp70Ab
Hsp70Bbb
Hsp70Bc
Clect27
deltaTry
Eglp4
gammaTry
Lcp3
Mhc
Vajk3
CG30031
CG32603
CG34446
CG43117
CG13023
Amnionless
att−ORFB
GstD10
Lsm10
Pepck
Tmem18
Wnt2
CG11300
CG14798
CG34188
CG18853
Ag5r
Cda9
Jon25Bi
Jon65Aiii
Jon65Aiv
Kaz−m1
LysD
Mco4
Muc26B
Muc96D
Mur29B
obst−F
obst−G
obst−H
obst−J
Pebp1
Peritrophin−15a
Peritrophin−15b
pgant4
PH4alphaPV
tgy
Tsp29Fb
yip7
CG10140
CG10154
CG10405
CG10725
CG10962
CG11470
CG11672
CG12934
CG13323
CG13324
CG13806
CG14273
CG14300
CG15043
CG15153
CG17147
CG17826
CG30025
CG32302
CG33985
CG34251
CG34282
CG43896
CG5084
CG5399
CG5506
CG6403
CG6933
CG7017
CG7252
CG7567
CG7714
CG7715
CG8560
CG9988
0
100
C
relative RG expression
Pebp1
CG14789
LysD
12hr
16hr
44hr

Figure S2. Differentially expressed genes in response to iron enrichment in brain-ring gland complexes and isolated ring glands.

A) Line graphs show a cluster profile (left) and an example gene (right) from the corresponding cluster. Y-axes represent precent RPKM based on the RNA-Seq data. The highest RPKM value of a given gene was set to 100%, and the remaining seven values were normalized accordingly. X-axes numbers represent hours after the switch in diets. In clusters graphs, values for each time point represent the average expression level (in percent) from all genes present in a specified cluster. Error bars represent standard error for each time point and the center represents the average. Asterisks show *P*-values (*$p < 0.05$ and ***$p < 0.001$). **B)** Heatmap of clustered 88 differentially expressed genes in BRGC samples, where the highest RPKM value of a given gene was set to 100 (red), and the remaining seven values were normalized accordingly. **C)** qPCR expression profiles of three random genes in ring glands (RG) dissected from third instar (L3) larvae at 12, 16 and 44 hours after the L2/L3 molt. Larvae were reared on iron-depleted (BPS) or normal diets (no suppl.). Expression was analyzed with three biological replicates, each tested in triplicate. Error bars represent 95% confidence intervals and asterisks indicate the *P*-values according to a two-sided Student's *t*-test (*$p < 0.05$ and ***$p < 0.01$). In A and C, the means are centered. Source data are available in the accompanying source data file.

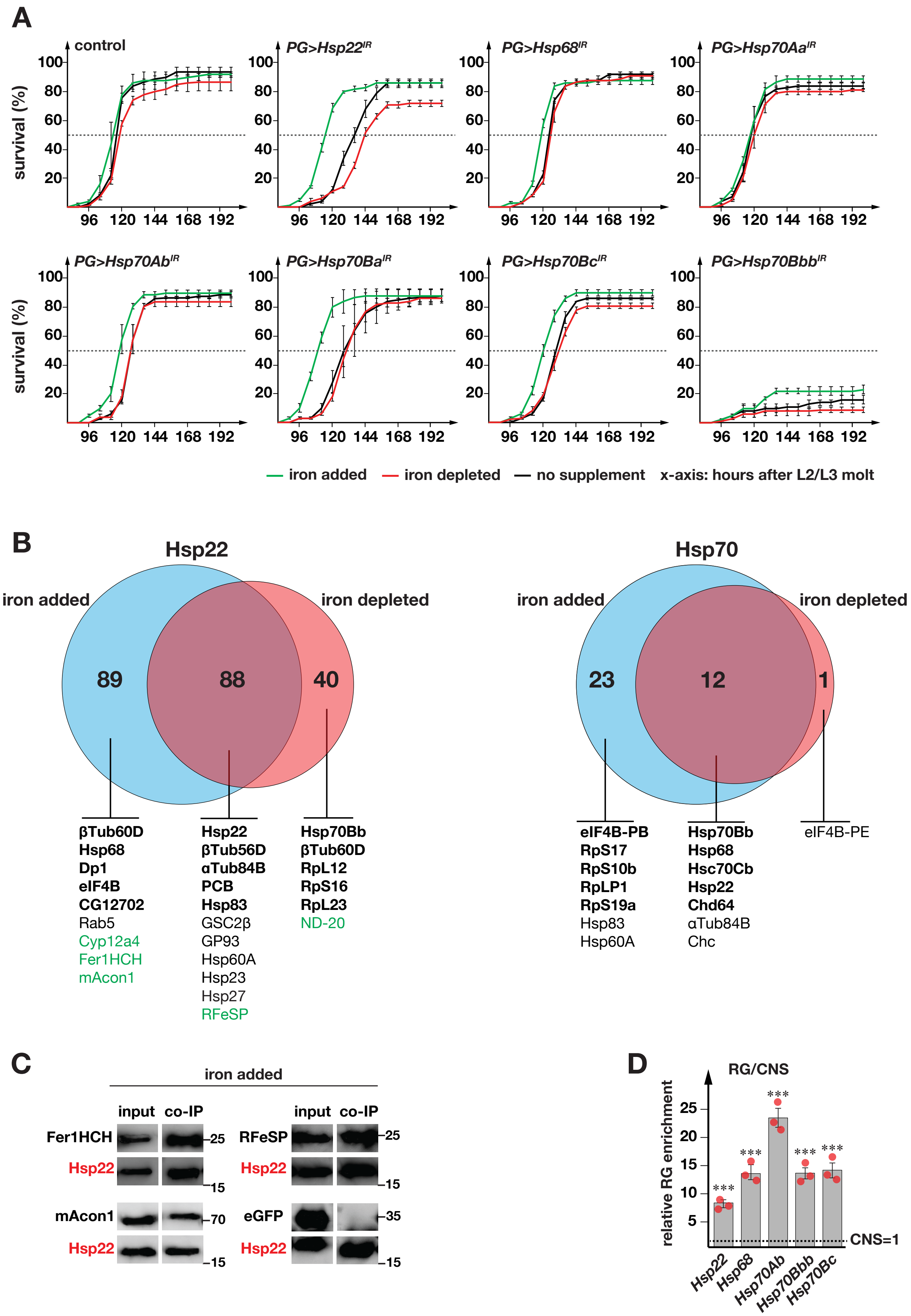

A
control
PG>Hsp22^IR
PG>Hsp68^IR
PG>Hsp70Aa^IR
PG>Hsp70Ab^IR
PG>Hsp70Ba^IR
PG>Hsp70Bc^IR
PG>Hsp70Bbb^IR
survival (%)
100
80
60
40
20
96 120 144 168 192
iron added
iron depleted
no supplement
x-axis: hours after L2/L3 molt
B
Hsp22
iron added
iron depleted
89
88
40
βTub60D
Hsp68
Dp1
eIF4B
CG12702
Rab5
Cyp12a4
Fer1HCH
mAcon1
Hsp22
βTub56D
αTub84B
PCB
Hsp83
GSC2β
GP93
Hsp60A
Hsp23
Hsp27
RFeSP
Hsp70Bb
βTub60D
RpL12
RpS16
RpL23
ND-20
Hsp70
iron added
iron depleted
23
12
1
eIF4B-PB
RpS17
RpS10b
RpLP1
RpS19a
Hsp83
Hsp60A
Hsp70Bb
Hsp68
Hsc70Cb
Hsp22
Chd64
αTub84B
Chc
eIF4B-PE
C
iron added
input co-IP
input co-IP
Fer1HCH
Hsp22
-25
-15
RFeSP
Hsp22
-25
-15
mAcon1
Hsp22
-70
-15
eGFP
Hsp22
-35
-15
D
RG/CNS
relative RG enrichment
25
20
15
10
5
***
CNS=1
Hsp22
Hsp68
Hsp70Ab
Hsp70Bbb
Hsp70Bc

Figure S3. Examining links between heat-shock proteins and iron metabolism.

A) Graphs showing survival and developmental timing of populations where we triggered RNAi in the PG (*PG>UAS*-RNAi) against seven heat-shock genes. *PG>*w^{1118} flies served as controls. Experiments were carried out on standard media (black), iron-depleted diets (supplemented with BPS, red), and iron-enriched food (supplemented with FAC, green). Y-axes denote the percentage of pupariated animals, and X-axes show hours after egg deposition. Error bars represent standard errors of three replicates. **B)** Venn diagrams showing proteins identified via MALDI-TOF mass spectrometry that were co-immunoprecipitated with Hsp22 and Hsp70 from S2 cells grown in iron-supplemented and iron-depleted media. The proteins shown in bold represent the five highest-scoring proteins in each category. Proteins with known links to iron biology are shown in green. **C)** Western Blot to detect proteins that co-immunoprecipitated with Hsp22 and Hsp70. S2 cells were transfected with plasmids expressing Hsp22-3xFLAG as bait and Myc-tagged Fer1HCH, RFeSP, mAcon1, eGFP as prey. **D)** qPCR analysis of Heat-shock protein genes (*Hsp*) in the RG and in the CNS to examine their relative transcript abundance in the RG. RGs were isolated from w^{1118} larvae. Error bars represent 95% confidence intervals and asterisks indicate the *p* values in a two-sided Student's *t*-test (*p $<$ 0.05 and ***p $<$ 0.01). In A and D, the means are centered. Source data are available in the accompanying source data file.

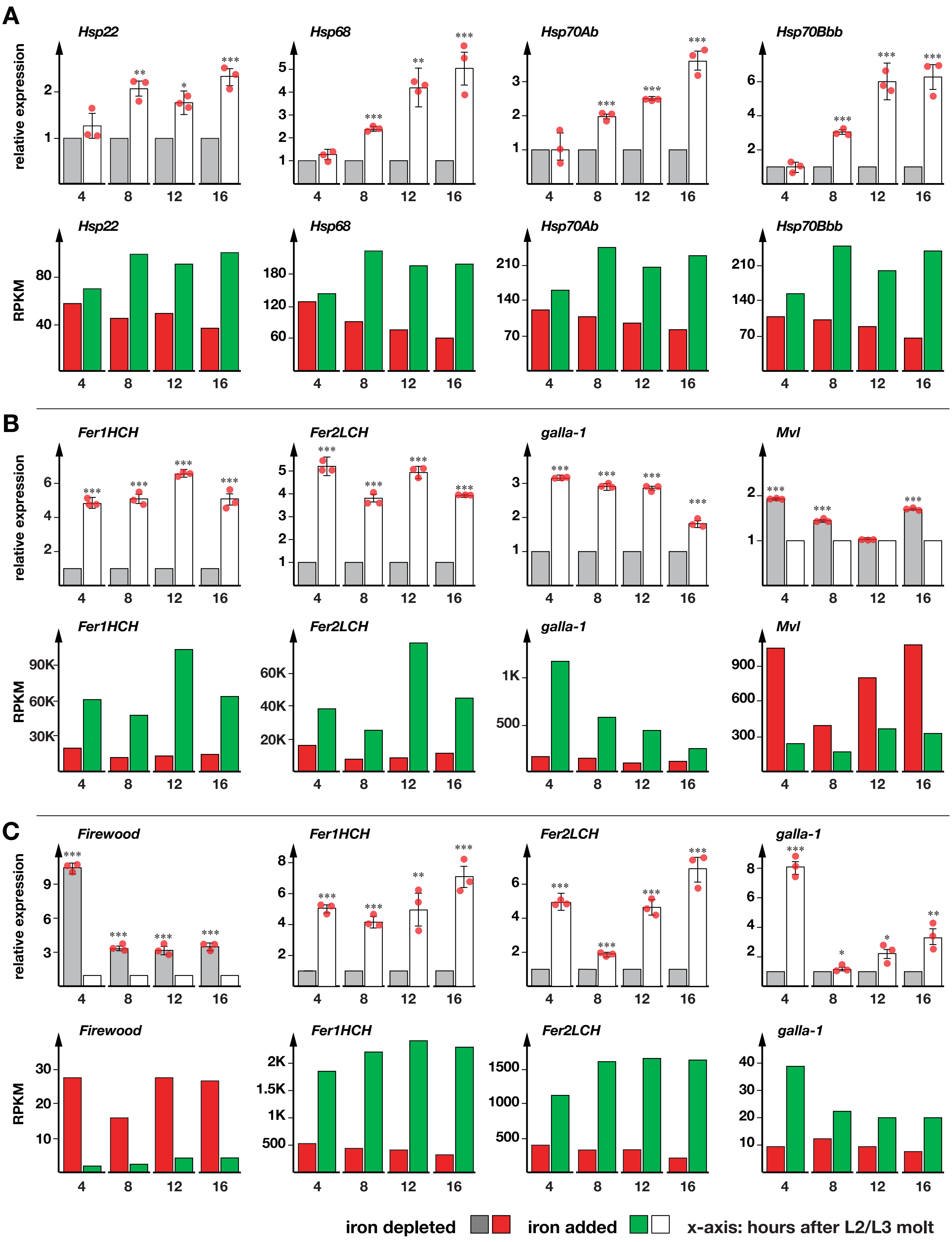
A
Hsp22
Hsp68
Hsp70Ab
Hsp70Bbb
relative expression
RPKM
B
Fer1HCH
Fer2LCH
galla-1
Mvl
relative expression
RPKM
C
Firewood
Fer1HCH
Fer2LCH
galla-1
relative expression
RPKM
iron depleted
iron added
x-axis: hours after L2/L3 molt

Figure S4. Validation of selected genes in BRGC, gut and WB samples.

A) Expression profiles of selected genes for BRGC (brain ring gland complex) samples. **B)** Expression profiles of selected genes in gut samples. **C)** Expression profiles of selected genes in whole-body samples. **A-C)** The lower rows (red/green) show RNA-seq results for selected genes at 4, 8, 12 and 16 hours after the L2/L3 molt (in RPKM), and the upper rows (grey/white) show the corresponding profiles based on qPCR. Flies were reared in iron-depleted (red) and iron-enriched diets (green). qPCR results are based on three biological replicates, each tested in triplicate. Error bars indicate the 95% confidence intervals. Asterisks are *p* values calculated via a two-sided Student's t-test ($*p < 0.05$ and $***p < 0.001$). In A-C, the means are centered. Source data are available in the accompanying source data file.

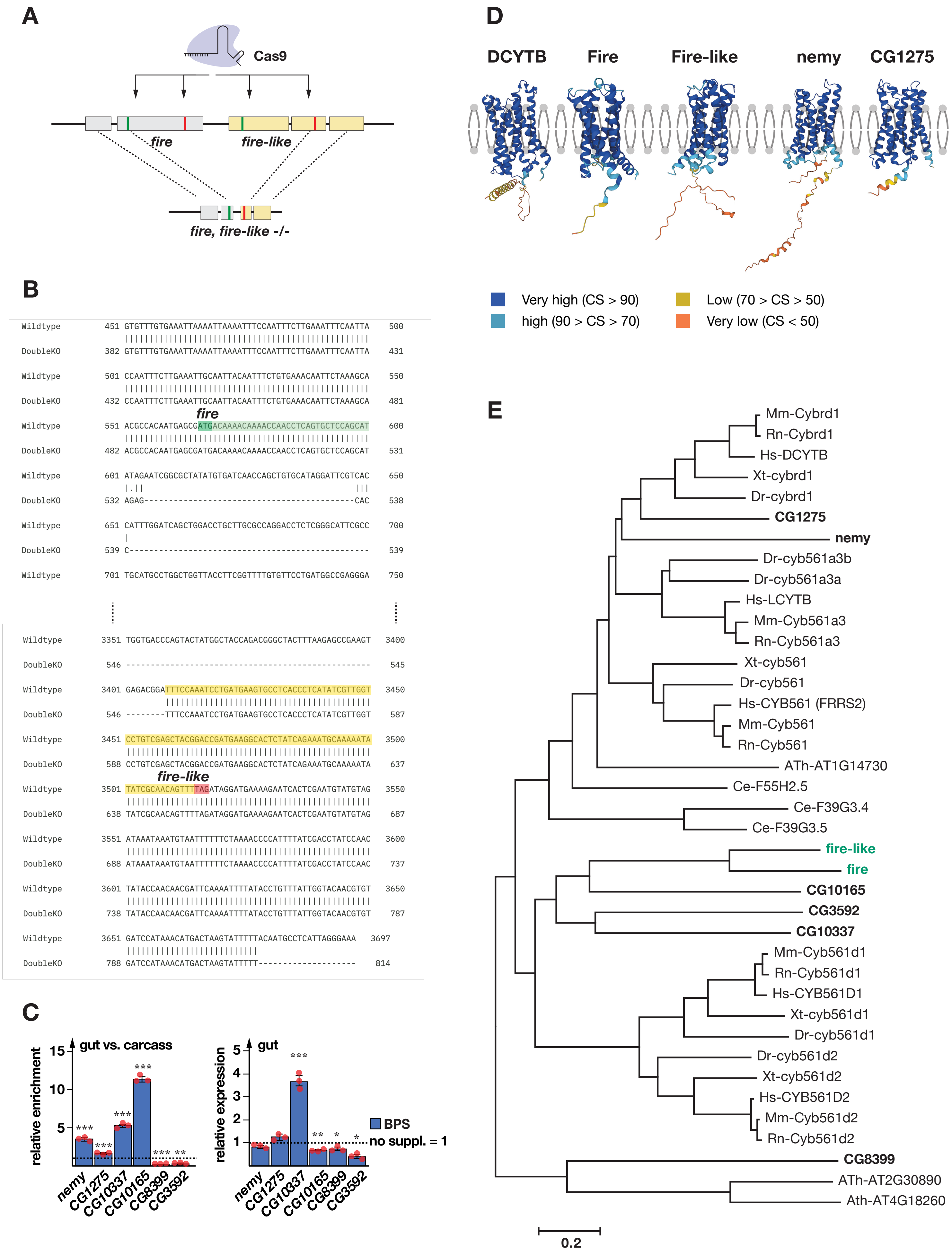

A
Cas9
fire
fire-like
fire, fire-like -/-
B
Wildtype 451 GTGTTTGTGAAATTAAAATTAAAATTTCCAATTTCTTGAAATTTCAATTA 500
DoubleKO 382 GTGTTTGTGAAATTAAAATTAAAATTTCCAATTTCTTGAAATTTCAATTA 431
Wildtype 501 CCAATTTCTTGAAATTGCAATTACAATTTCTGTGAAACAATTCTAAAGCA 550
DoubleKO 432 CCAATTTCTTGAAATTGCAATTACAATTTCTGTGAAACAATTCTAAAGCA 481
fire
Wildtype 551 ACGCCACAATGAGCGATGACAAAACAAAACCAACCTCAGTGCTCCAGCAT 600
DoubleKO 482 ACGCCACAATGAGCGATGACAAAACAAAACCAACCTCAGTGCTCCAGCAT 531
Wildtype 601 ATAGAATCGGCGCTATATGTGATCAACCAGCTGTGCATAGGATTCGTCAC 650
DoubleKO 532 AGAG--CAC 538
Wildtype 651 CATTTGGATCAGCTGGACCTGCTTGCGCCAGGACCTCTCGGGCATTCGCC 700
DoubleKO 539 C--- 539
Wildtype 701 TGCATGCCTGGCTGGTTACCTTCGGTTTTGTGTTCCTGATGGCCGAGGGA 750
Wildtype 3351 TGGTGACCCAGTACTATGGCTACCAGACGGGCTACTTTAAGAGCCGAAGT 3400
DoubleKO 546 -- 545
Wildtype 3401 GAGACGGATTTCCAAATCCTGATGAAGTGCCTCACCCTCATATCGTTGGT 3450
DoubleKO 546 --------TTTCCAAATCCTGATGAAGTGCCTCACCCTCATATCGTTGGT 587
Wildtype 3451 CCTGTCGAGCTACGGACCGATGAAGGCACTCTATCAGAAATGCAAAAATA 3500
DoubleKO 588 CCTGTCGAGCTACGGACCGATGAAGGCACTCTATCAGAAATGCAAAAATA 637
fire-like
Wildtype 3501 TATCGCAACAGTTTTAGATAGGATGAAAAGAATCACTCGAATGTATGTAG 3550
DoubleKO 638 TATCGCAACAGTTTTAGATAGGATGAAAAGAATCACTCGAATGTATGTAG 687
Wildtype 3551 ATAAATAAATGTAATTTTTTTCTAAAACCCCATTTTATCGACCTATCCAAC 3600
DoubleKO 688 ATAAATAAATGTAATTTTTTTCTAAAACCCCATTTTATCGACCTATCCAAC 737
Wildtype 3601 TATACCAACAACGATTCAAAATTTTATACCTGTTTATTGGTACAACGTGT 3650
DoubleKO 738 TATACCAACAACGATTCAAAATTTTATACCTGTTTATTGGTACAACGTGT 787
Wildtype 3651 GATCCATAAACATGACTAAGTATTTTTACAATGCCTCATTAGGGAAA 3697
DoubleKO 788 GATCCATAAACATGACTAAGTATTTTT-------------------- 814
C
gut vs. carcass
relative enrichment
15
10
5
***
***
***
***
***
**
nemy
CG1275
CG10337
CG10165
CG8399
CG3592
gut
relative expression
5
4
3
2
1
***
**
*
*
BPS
no suppl. = 1
D
DCYTB
Fire
Fire-like
nemy
CG1275
Very high (CS > 90)
high (90 > CS > 70)
Low (70 > CS > 50)
Very low (CS < 50)
E
Mm-Cybrd1
Rn-Cybrd1
Hs-DCYTB
Xt-cybrd1
Dr-cybrd1
CG1275
nemy
Dr-cyb561a3b
Dr-cyb561a3a
Hs-LCYTB
Mm-Cyb561a3
Rn-Cyb561a3
Xt-cyb561
Dr-cyb561
Hs-CYB561 (FRRS2)
Mm-Cyb561
Rn-Cyb561
ATh-AT1G14730
Ce-F55H2.5
Ce-F39G3.4
Ce-F39G3.5
fire-like
fire
CG10165
CG3592
CG10337
Mm-Cyb561d1
Rn-Cyb561d1
Hs-CYB561D1
Xt-cyb561d1
Dr-cyb561d1
Dr-cyb561d2
Xt-cyb561d2
Hs-CYB561D2
Mm-Cyb561d2
Rn-Cyb561d2
CG8399
ATh-AT2G30890
Ath-AT4G18260
0.2

Figure S5. Fire and fire-like sequence analysis.

A) Schematic representation illustrating the target sites of the gRNAs used for generating *fire*$^{-/-}$, *fire-like*$^{-/-}$ double mutant (aka *fire*^{2xKO}) animals. **B)** DNA sequence alignment of control (*w*^{1118}) and *fire*^{2xKO} mutant animals. The *fire* gene sequence is highlighted in green, and fire-like is highlighted in yellow. **C)** Left: is qPCR analysis of six remaining CYB561 genes *(nemy*, *CG5157*, *CG10337*, *CG10165*, *CG8399* and *CG3592).* Samples based on guts and carcass (whole larva minus gut) to assess transcript abundance in the gut relative to carcass. Right: qPCR analysis of *nemy*, *CG5157*, *CG10337*, *CG10165*, *CG8399* and *CG3592* genes in the gut samples of animals raised under normal conditions (no suppl.) and on medium supplemented with BPS. Expression was analyzed in 44-hour L3 larvae in three biological replicates, each tested in triplicate. Error bars represent 95% confidence intervals and asterisks indicate the *P*-values according to a two-sided Student's *t*-test (*$p < 0.05$ and ***$p < 0.01$). The centers of error bars represent the mean. **D)** AlphaFold-based protein structure predictions of human DCYTB and *Drosophila* Fire, Fire-like, Nemy and CG1275. Colour boxes represent the Confidence Score (CS) of the protein structures. **E)** Phylogenic tree of CYB561 proteins family based on sequences from *Homo sapiens* (Hs), *Mus musculus* (Mm), *Rattus norvegicus* (Rn), *Xenopus tropicalis* (Xt), *Danio rerio* (Dn), *Caenorhabditis elegans* (Ce), Arabidopsis thaliana (ATh) and *Drosophila melanogaster* (bold) DcytB proteins. Fire and Fire-like proteins are highlighte in green. The scale bar indicates 0.2 substitution per site. Source data are available in the accompanying source data file.

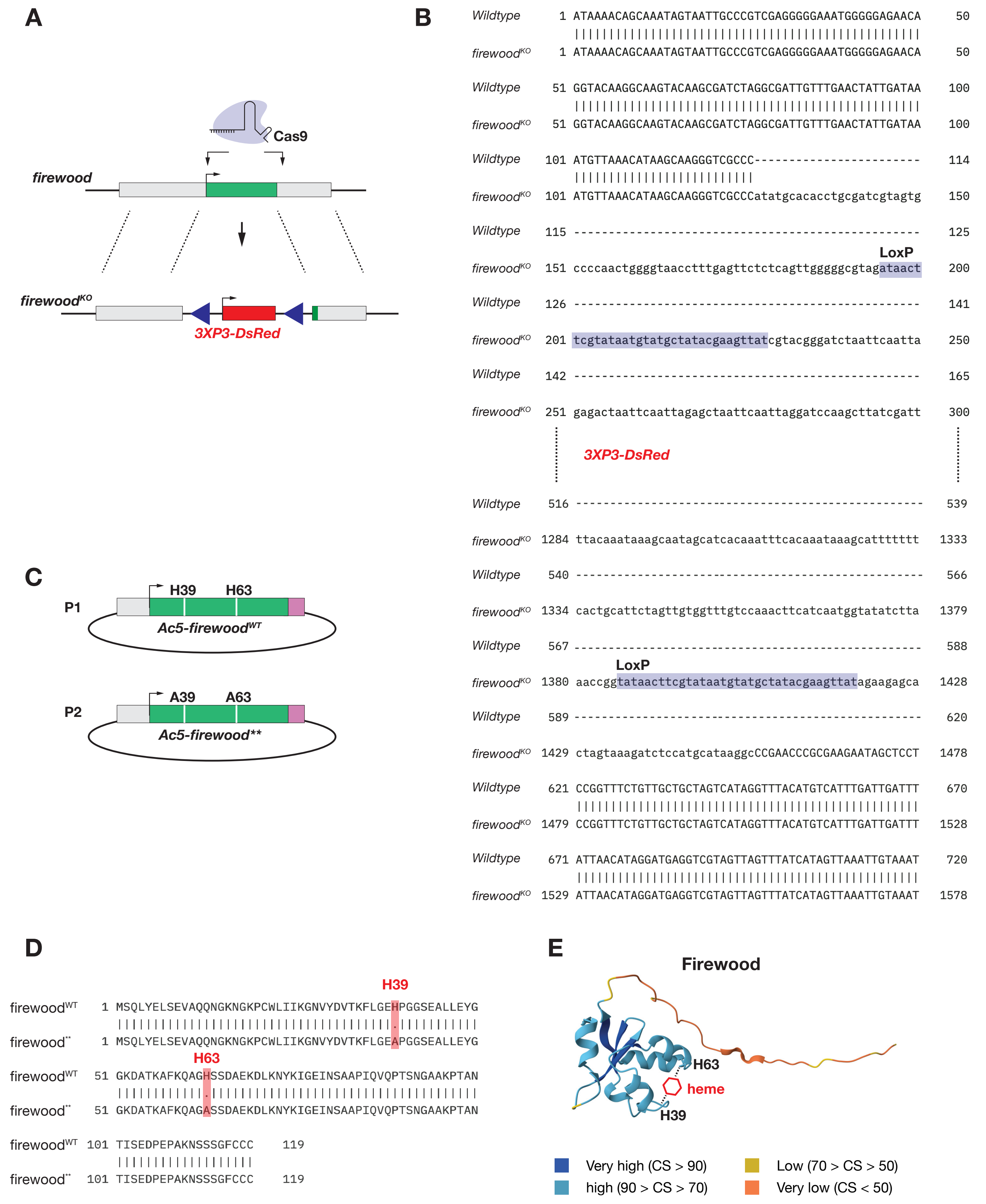
A
Cas9
firewood
firewoodKO
3XP3-DsRed
B
Wildtype 1 ATAAAACAGCAAATAGTAATTGCCCGTCGAGGGGGAAATGGGGGAGAACA 50
firewoodKO 1 ATAAAACAGCAAATAGTAATTGCCCGTCGAGGGGGAAATGGGGGAGAACA 50
Wildtype 51 GGTACAAGGCAAGTACAAGCGATCTAGGCGATTGTTTGAACTATTGATAA 100
firewoodKO 51 GGTACAAGGCAAGTACAAGCGATCTAGGCGATTGTTTGAACTATTGATAA 100
Wildtype 101 ATGTTAAACATAAGCAAGGGTCGCCC------------------------ 114
firewoodKO 101 ATGTTAAACATAAGCAAGGGTCGCCCatatgcacacctgcgatcgtagtg 150
Wildtype 115 -- 125
LoxP
firewoodKO 151 ccccaactggggtaacctttgagttctctcagttgggggcgtagataact 200
Wildtype 126 -- 141
firewoodKO 201 tcgtataatgtatgctatacgaagttatcgtacgggatctaattcaatta 250
Wildtype 142 -- 165
firewoodKO 251 gagactaattcaattagagctaattcaattaggatccaagcttatcgatt 300
3XP3-DsRed
Wildtype 516 -- 539
firewoodKO 1284 ttacaaataaagcaatagcatcacaaatttcacaaataaagcatttttt 1333
Wildtype 540 -- 566
firewoodKO 1334 cactgcattctagttgtggtttgtccaaacttcatcaatggtatatctta 1379
Wildtype 567 -- 588
LoxP
firewoodKO 1380 aaccggtataacttcgtataatgtatgctatacgaagttatagaagagca 1428
Wildtype 589 -- 620
firewoodKO 1429 ctagtaaagatctccatgcataaggcCCGAACCCGCGAAGAATAGCTCCT 1478
Wildtype 621 CCGGTTTCTGTTGCTGCTAGTCATAGGTTTACATGTCATTTGATTGATTT 670
firewoodKO 1479 CCGGTTTCTGTTGCTGCTAGTCATAGGTTTACATGTCATTTGATTGATTT 1528
Wildtype 671 ATTAACATAGGATGAGGTCGTAGTTAGTTTATCATAGTTAAATTGTAAAT 720
firewoodKO 1529 ATTAACATAGGATGAGGTCGTAGTTAGTTTATCATAGTTAAATTGTAAAT 1578
C
P1
H39
H63
Ac5-firewoodWT
P2
A39
A63
Ac5-firewood**
D
H39
firewoodWT 1 MSQLYELSEVAQQNGKNGKPCWLIIKGNVYDVTKFLGEHPGGSEALLEYG
firewood** 1 MSQLYELSEVAQQNGKNGKPCWLIIKGNVYDVTKFLGEAPGGSEALLEYG
H63
firewoodWT 51 GKDATKAFKQAGHSSDAEKDLKNYKIGEINSAAPIQVQPTSNGAAKPTAN
firewood** 51 GKDATKAFKQAGASSDAEKDLKNYKIGEINSAAPIQVQPTSNGAAKPTAN
firewoodWT 101 TISEDPEPAKNSSSGFCCC 119
firewood** 101 TISEDPEPAKNSSSGFCCC 119
E
Firewood
H63
heme
H39
Very high (CS > 90)
high (90 > CS > 70)
Low (70 > CS > 50)
Very low (CS < 50)

Figure S6. Generation of *firewood* knockout line and design of ex-vivo constructs.

A) Schematic representation of the CRISPR/Cas9 strategy used to generate the *firewood*^{KO} line. The wild-type locus (green box) was replaced with 3xP3-DsRed (red box) flanked by two LoxP sequences (blue triangles). **B)** DNA sequence alignment of control (*firewood*^{WT}) and *firewood*^{KO} mutant animals. *loxP* sites are highlighted in purple. **C)** Schematic of plasmids used for the ex vivo ferric reductase assay in S2 cells, carrying either wild-type (WT) or double mutant (*firewood***) cDNAs. Histidine 39 and Histidine 63 (red lines), predicted heme-binding residues, were replaced with alanine. All constructs include a C-terminal 3x-FLAG tag (red box). **D)** Amino acid sequence alignment of wild-type (Firewood^{WT}) and mutant (Firewood**) Firewood. The His-to-ALA substitutions are highlighted in red. **E)** Predicted structure of Firewood based on AlphaFold. The heme group is depicted as a red hexagon, black dotted lines indicate putative coordination with His39 and His63. Coloured squares denote AlphaFold confidence scores (CS) for predicted structures.

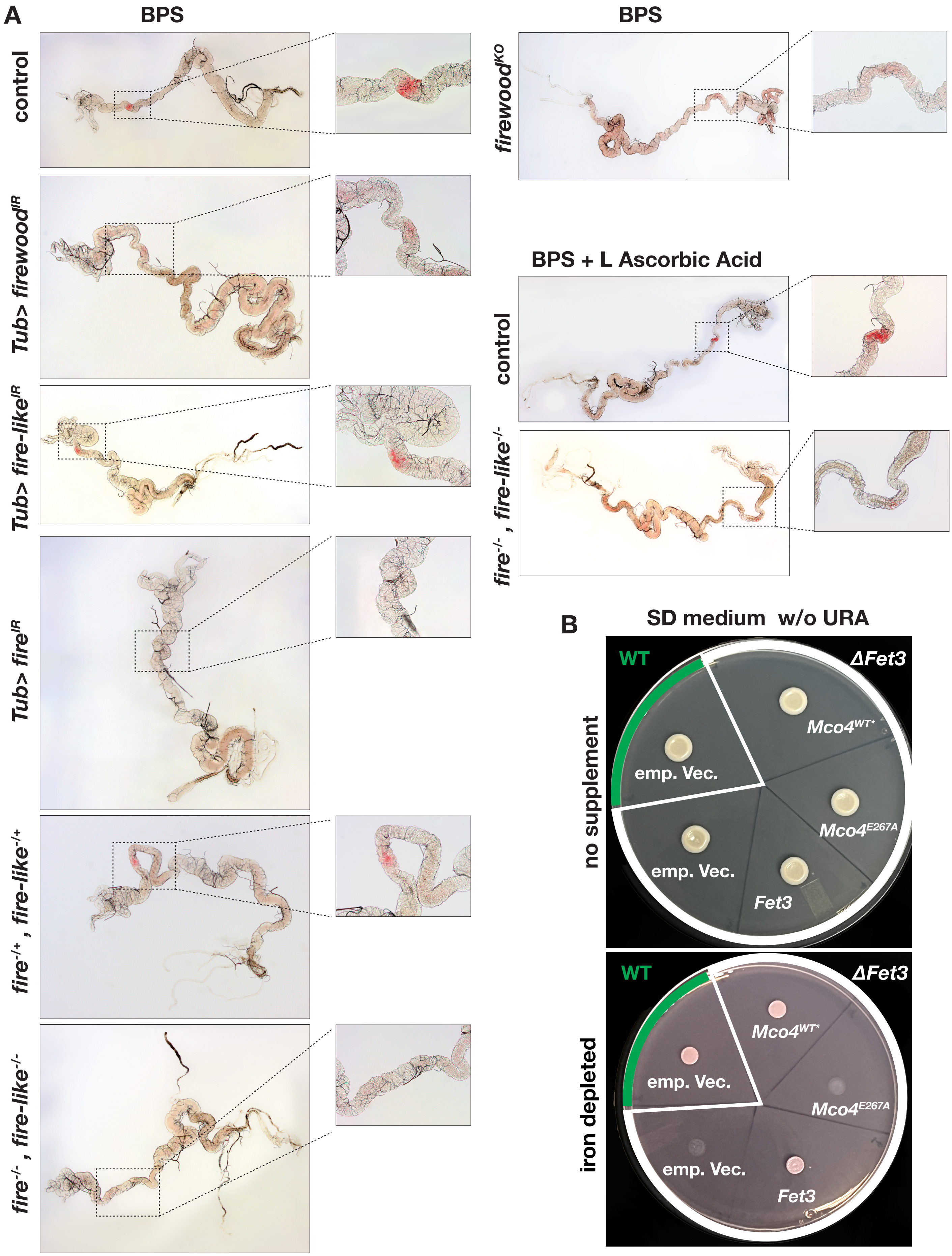

A
BPS
control
Tub> firewood^IR
Tub> fire-like^IR
Tub> fire^IR
fire-/+ , fire-like-/+
fire-/- , fire-like-/-
BPS
firewood^KO
BPS + L Ascorbic Acid
control
fire-/- , fire-like-/-
B
SD medium w/o URA
no supplement
WT
ΔFet3
Mco4^WT*
emp. Vec.
emp. Vec.
Mco4^E267A
Fet3
iron depleted
WT
ΔFet3
Mco4^WT*
emp. Vec.
Mco4^E267A
emp. Vec.
Fet3

Figure S7. Uncropped images corresponding to Figures 4C and 6I.

A) Uncropped brightfield images of larval midguts corresponding to Figure 4C, including control (w^{1118}), *Tub>fire*^{IR}, *Tub>fire-like*^{IR}, *Tub>firewood*^{IR}, *firewood*^{KO}, *fire*$^{-/-}$, *fire-like*$^{-/-}$ and *fire, fire-like*$^{+/-}$ genotypes. Red precipitates indicate the BPS precipitation region (BPR). **B)** Uncropped images of yeast colonies corresponding to Figure 6I. Yeast cells were transformed with the indicated plasmids and grown on synthetic dropout medium lacking uracil (SD –URA) and supplemented with 80 μM BPS.

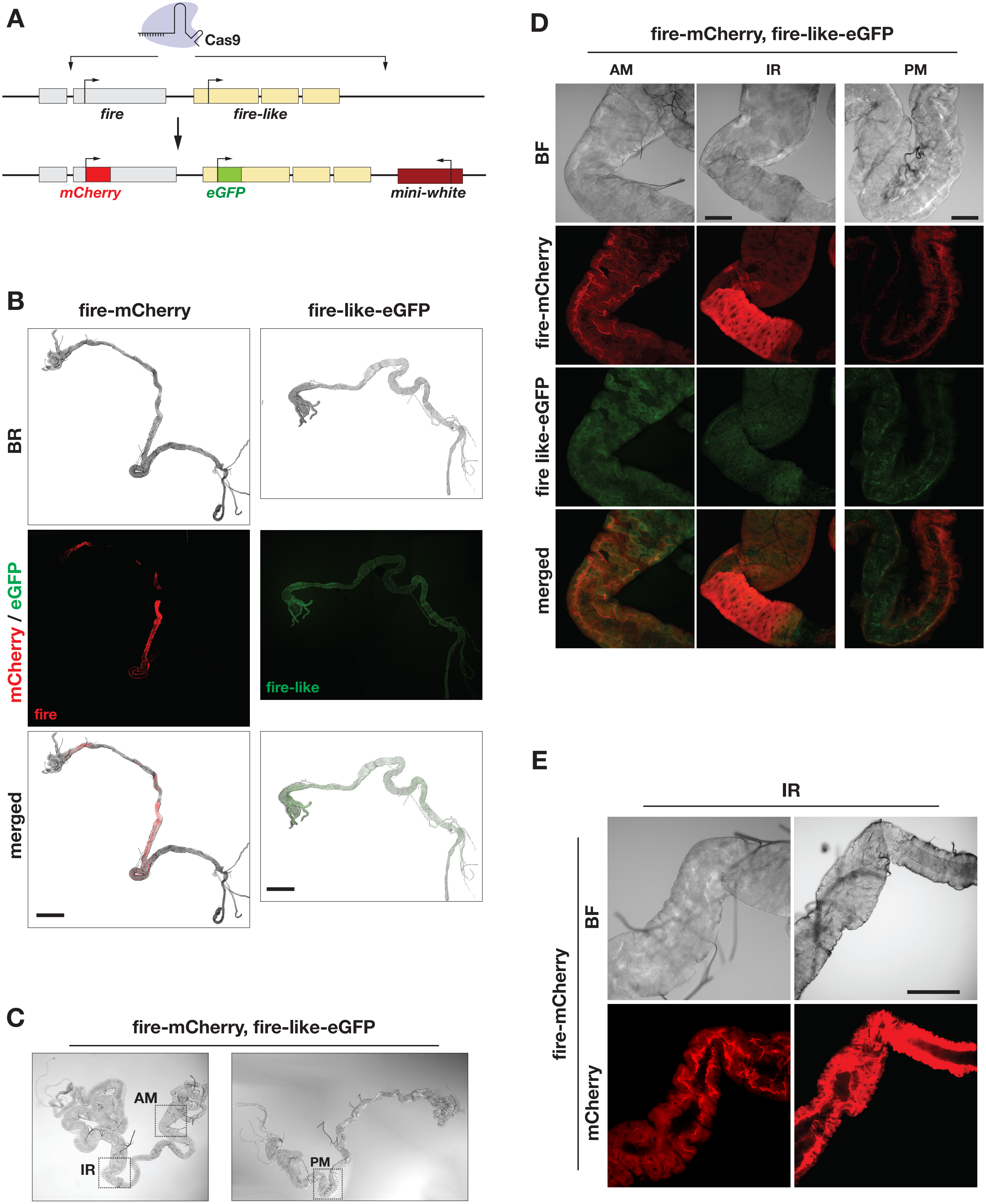

A
Cas9
fire
fire-like
mCherry
eGFP
mini-white
B
fire-mCherry
fire-like-eGFP
BR
mCherry / eGFP
fire
fire-like
merged
C
fire-mCherry, fire-like-eGFP
AM
IR
PM
D
fire-mCherry, fire-like-eGFP
AM
IR
PM
BF
fire-mCherry
fire like-eGFP
merged
E
IR
fire-mCherry
BF
mCherry

Figure S8. Fire and fire-like double knock-in line.

A) Schematic of the *fire-mCherry, fire-like-eGFP* knock-in locus. The endogenous *fire* and *fire-like* transcription units were replaced with N-terminally tagged version carrying mCherry (red box) or eGFP (green box), respectively. A *mini-white* marker gene (dark red box) was inserted downstream of the *fire-like* locus to facilitate screening after embryo injection. **B)** Fluorescent images of dissected guts from *fire-mCherry, fire-like-eGFP* larvae reared on iron-depleted (BPS-supplemented) diets. Scale bars: 100 µm. **C)** Corresponding brightfield images of the same whole gut samples shown in (B), with dotted boxes indicating the anterior midgut (AM), iron region (IR), and posterior midgut (PM). **D)** Fluorescent images of the AM, IR and PM regions from *fire-mCherry, fire-like-eGFP* larvae reared on iron-depleted diets (BPS). **E)** Single plane fluorescent images of the iron region (IR) of *fire-mCherry* animals reared on iron-depleted diets (BPS). In panels B, D, and E, the gray channel shows brightfield (BF), while red and green channels show Fire-mCherry and Fire-like-eGFP, respectively. Scale bars in D and E: 80 µm.

```
ScFET3 --------------------------------------------------  0
DmMco4 MKFNLVQTLVFTLCLISVQIYGIQDASGKRIVSKYERIMQMYPQLSSGAG  50

ScFET3 --------------------------------------------------  0
DmMco4 ESSQWRAAEKDNQRHPCRRDCADKQPMTCYYYMVVHYDDTMAETCKRYLQ  100

ScFET3 ------MTNALLSIAVLLFSMLSLAQAETHTFNWTTGWDYRNVDGLKSRP  44
DmMco4 SKFRFKLSGKEYIDGIALATQLAAND------------DCKYADGLESE-  137

ScFET3 VITCNGQFPWPDITVNKGDRVQIYLTNGMNNTNTSMHFHGLFQNGTASMD  94
DmMco4 VMVVNGQLPGMNIEVCYGDTVVADVINSMHET-TTIHWHGMHQRLTPFMD  186

ScFET3 GVPFLTQCPIAPGSTMLYNFTVDYNVGTYWYHSHTDGQYEDGMKGLFIIK  144
DmMco4 GVPHVTQYPIEAGQAFRYRFEVDHG-GTNWWHSHTEHQRAFGLAGPLVVR  235

                                                    E185
ScFET3 ------DDSFPYDYD-EELSLSLSEWYHDLVTDLTKSFMSVYNPTGAEPI  187
DmMco4 MPPKLNPHAHLYDFDMSEHVIMIQDWVHNFV----------------ESV  269

ScFET3 PQNLIVNN--------------TMNLTWEVQPDTTYLLRIVNVGGFVSQ-  222
DmMco4 AENILINGRGRNLKKGVKAAKPTLYAHFPVVRGGRYRFRVIFNG--VSNC  317

ScFET3 --YFWIEDHEMTVVEIDGITTEKNVTDMLYITVAQRYTVLVHTKNDTD--  268
DmMco4 PISFSIDKHDLVVIASDGNDIEPVEVQRIMFHGAERFDFVLHANQEVSNY  367

                        D283
ScFET3 ----KNFAIMQK---FDDTMLDVIPSDLQLNATSYMVYNKTAALPTQNYV  311
DmMco4 WIRVKGYSFCAKNQLHQEAVLHYRDADTRALDTHTLSYAYDAPGKTLNEL  417

ScFET3 ----------DSIDNFLDDFYLQPYEKEAIYGEPDHVITVDVVMDNLKNG  351
DmMco4 GDDASGARAGNSIS--LANLNAQRPEPEV---APSVTFYTSMNAFEVRQG  462

ScFET3 VNYAF-FNNITYTAPKVPTLMT--------VLSSGDQA----NNSEIYGS  388
DmMco4 EGFRFQMDDISFSMPKMSLLQTRNLGVGQFFCNRSQQADLGFNCRQRHCQ  512

                     D409
ScFET3 NTHTFILEKDEIVEIVLNNQDTGTHPFHLHGHAFQTIQRDRTYDDALGEV  438
DmMco4 CSNVIQVPANQQVEFVISSLSQTPHPIHLHGYTFRVVGMGVLGEQKIGQI  562

ScFET3 PHSFDPDNHPAFPE----YPMRRDTLYVRPQSNFVIRFKADNPGVWFFHC  484
DmMco4 EQI---DKKTPLPRRAKGAPL-KDSVQVPAFGYTILRFYSNSPGYWMFHC  608

ScFET3 HIEWHLLQGLGLVLVEDPFGIQDAHSQQLSENHLEVCQSCSVATEGNAAA  534
DmMco4 HISPHSENGMAAVVRVG----EDVEMKMCPVSNCGLCSSVA---------  645

ScFET3 NTLDLTDLTGENVQHAFIPTGFTKKGIIAMTFSCFAGILGIITIAIYGMM  584
DmMco4 --------------------------------------------------  645

ScFET3 DMEDATEKVIRDLHVDPEVLLNEVDENEERQVNEDRHSTEKHQFLTKAKR  634
DmMco4 --------------------------------------------------  645

ScFET3 FF 636
DmMco4 -- 645
```

iron-binding   copper-binding

Figure S9. Amino acid sequence alignment of Fet3p and Mco4.

Protein sequence alignment of Mco4 reveals putative iron- and copper-binding sites in *Drosophila* Mco4 (DmMco4) compared to amino acids with identified functionality in *Saccharomyces cerevisiae* Fet3p (ScFET3). Iron-binding amino acids are highlighted as blue boxes, while copper-binding sites are marked by green boxes. The residues E185, D283, and D409 correspond to glutamic acid 185, aspartic acid 283, and aspartic acid 409, which are iron-binding residues in yeast Fet3p[89].

Supplementary Tables and Supplementary Data Files

Table S1. List of fly stocks.

**Name**[a]	**Stock ID**[b]	**Name**[a]	**Stock ID**[b]
w^{1118}	3605	*UAS-CG7763*^{IR}	105878
phm22-GAL4	-	*UAS-CG33474*^{IR}	102875
aTub84B-GAL4	5138	*UAS-CG33270*^{IR}	109109
NP3084-GAL4	113094	*UAS-MtnA*^{IR}	105011
Vas.Cas9	51323	*UAS-MtnB*^{IR}	106118
Act-Cas9	54590	*UAS-Ctr1B*^{IR}	57710
Mco4^{KO}	-	*UAS-Zip89B*^{IR}	32954
Mco4^{3xFLAG}	-	*UAS-galla-1*^{IR}	105959
UAS-Mco4-3xMyc	-	*UAS-RpS5b*^{IR}	57147
fire^{2xKo}	-	*UAS-CG33061*^{IR}	101976
firewood^{Ko}	-	*UAS-Drip*^{IR}	106911
Mvl[97f]	5151	*UAS-Fer2LCH*^{IR}	106960
Fire-mCherry,fire-like-eGFP	-	*UAS-Fer1HCH*^{IR}	102406
UAS-fire^{IR}	100234	*UAS-Hsp68*^{IR}	50637
UAS-Fire-like^{IR}	105526	*UAS-Zip99C*^{IR}	50635
UAS-Firewwod^{IR}	101878	*UAS-AGBE*^{IR}	108087
UAS-Desi^{IR}	105692	*UAS-Hsp22*^{IR}	107356
UAS-CG13454^{IR}	102723	*UAS-Hsp70Aa*^{IR}	35671
UAS-yellow-k^{IR}	109040	*UAS-Hs970Ab*^{IR}	35663
UAS-CG18179^{IR}	51451	*UAS-Hsp70Ba*^{IR}	35672
UAS-CG14626^{IR}	102275	*UAS-Hsp70Bc*^{IR}	35697
UAS-ABCA3^{IR}	105608	*UAS-Hsp70Bbb*^{IR}	33916
UAS-Ugt86Dc^{IR}	34095		

[a] **IR:** "Inverted Repeat", denotes RNAi line.
[b] **Stock IDs** are from KYOTO Stock Center, Bloomington Drosophila Stock Center and Vienna Drosophila Resource Center.

Supplementary data 1–5 Legends

Supplementary Data 1. List of primers used in this study.

Primers are listed as forward (Fw) and reverse (Re) pairs. Lowercase sequences in primers indicate overlap sequences for the Gibson assembly reaction. Sequences in red indicate gRNA sequences. Sequences in green indicate the *E. coli* T7 RNA polymerase promoter.

Supplementary Data 2. List of all differentially expressed genes (DEGs) identified via ArrayStar, DESeq2, and edgeR analysis.

In ArrayStar analysis, Geometric Means (GEOMean) of two replicates and Linear Fold Changes (Linear FC) are presented. In ArrayStar analysis, genes of each tissue are sorted based on the yellow-highlighted column. In DESeq2 and edgeR sheets, three analyses for each tissue are presented and statistically significant DEGs are found based on P value < 0.05. In DESeq2 and edgeR, the sustained analysis compares all time points of BPS supplementation versus all time points of FAC supplementation for each tissue. In DESeq2 and edgeR 4-hour (4-hr) and 16-hour (16-hr) analyses, DEGs are identified based on two replicates of 4-hr and 16-hr BPS supplementation versus two replicates of 4-hour and 16-hour FAC supplementation. Mco4, Fire, Fire-like and Firewood genes are shown in green. In DESeq2 and edgeR, P-values are two-sided and calculated using the Benjamini-Hochberg method.

Supplementary Data 3. List of 839 reference genes linked to iron/metals in the Drosophila melanogaster genome.

Genes were identified by screening the fly transcriptome for gene function annotations associated with 'iron' and other metal-related terms.

Supplementary Data 4. K-means cluster analysis of 750 differentially expressed genes (DEGs) in BRGC, gut, and WB samples.

For each cluster (C), the geometric mean of gene expression between two replicates is presented. The percentage of gene expression is calculated relative to the maximum geometric mean (yellow highlighted column) of each gene within the dataset. Genes linked to iron metabolism are shown in green.

Supplementary Data 5. List of proteins identified by MALDI-TOF following Co-immunoprecipitation of Hsp22 and Hsp70 in iron-depleted (BPS added) and iron-repleted (FAC added) media.

MS Score: The sum of the ion scores of all peptides that were identified. Coverage: The percentage of the protein sequence covered by identified peptides. # Proteins: The number of identified proteins in a protein group, that is, the number of proteins displayed in the Protein Group Members view. # Unique Peptides: The number of peptide sequences that are unique to a protein group. # Peptides: The total number of distinct peptide sequences identified in the protein group. # AAs: The number of Amino Acids. MW [kDa]: Molecular Weight [Kilodalton].
