# Supplementary material for: New molecular components of high and low affinity iron import systems in Drosophila
Source: Nat Commun. 2025 Jul 1;16:5662. doi: 10.1038/s41467-025-60758-6 (PMC12218971; doi:10.1038/s41467-025-60758-6)
Supplement: Supplementary file 3 — Description of Additional Supplementary Files [file 41467_2025_60758_MOESM3_ESM.pdf]

Description of Additional Supplementary Files

File Name: Supplementary Data 1
Description: List of all primers used in this study.

File Name: Supplementary Data 2
Description: List of all differentially expressed genes in this study.

File Name: Supplementary Data 3
Description: List of 839 reference genes linked to iron/metals in the *Drosophila*.

File Name: Supplementary Data 4
Description: K-means cluster analysis all differentially expressed genes in BRGC, gut, and WB samples.

File Name: Supplementary Data 5
Description: List of proteins identified by MALDI-TOF following Co-immunoprecipitation of Hsp22 and Hsp70.
