# Supplementary material for: New molecular components of high and low affinity iron import systems in Drosophila
Source: Nat Commun. 2025 Jul 1;16:5662. doi: 10.1038/s41467-025-60758-6 (PMC12218971; doi:10.1038/s41467-025-60758-6)
Supplement: Supplementary file 9 — Reporting Summary [file 41467_2025_60758_MOESM9_ESM.pdf]

nature portfolio

Corresponding author(s): Kirst King-Jones

Last updated by author(s): May 22, 2025

Reporting Summary

Nature Portfolio wishes to improve the reproducibility of the work that we publish. This form provides structure for consistency and transparency in reporting. For further information on Nature Portfolio policies, see our Editorial Policies and the Editorial Policy Checklist.

Statistics

For all statistical analyses, confirm that the following items are present in the figure legend, table legend, main text, or Methods section.

n/a	Confirmed	
☐	☒	The exact sample size (*n*) for each experimental group/condition, given as a discrete number and unit of measurement
☐	☒	A statement on whether measurements were taken from distinct samples or whether the same sample was measured repeatedly
☐	☒	The statistical test(s) used AND whether they are one- or two-sided<br>*Only common tests should be described solely by name; describe more complex techniques in the Methods section.*
☒	☐	A description of all covariates tested
☐	☒	A description of any assumptions or corrections, such as tests of normality and adjustment for multiple comparisons
☐	☒	A full description of the statistical parameters including central tendency (e.g. means) or other basic estimates (e.g. regression coefficient) AND variation (e.g. standard deviation) or associated estimates of uncertainty (e.g. confidence intervals)
☐	☒	For null hypothesis testing, the test statistic (e.g. *F*, *t*, *r*) with confidence intervals, effect sizes, degrees of freedom and *P* value noted<br>*Give P values as exact values whenever suitable.*
☒	☐	For Bayesian analysis, information on the choice of priors and Markov chain Monte Carlo settings
☒	☐	For hierarchical and complex designs, identification of the appropriate level for tests and full reporting of outcomes
☒	☐	Estimates of effect sizes (e.g. Cohen's *d*, Pearson's *r*), indicating how they were calculated

Our web collection on statistics for biologists contains articles on many of the points above.

Software and code

Policy information about availability of computer code

Data collection	All information regarding data collection is provided in the Materials and Methods section.
Data analysis	Data were analyzed using FastQC, Arraystar and R software, and all details related to data analysis are described in the Materials and Methods section.

For manuscripts utilizing custom algorithms or software that are central to the research but not yet described in published literature, software must be made available to editors and reviewers. We strongly encourage code deposition in a community repository (e.g. GitHub). See the Nature Portfolio guidelines for submitting code & software for further information.

Data

Policy information about availability of data

All manuscripts must include a data availability statement. This statement should provide the following information, where applicable:

- Accession codes, unique identifiers, or web links for publicly available datasets
- A description of any restrictions on data availability
- For clinical datasets or third party data, please ensure that the statement adheres to our policy

RNA-sequencing data are deposited in the NCBI database (PRJNA1149758), and protein mass spectrometry data are deposited in the PRIDE database (PXD055043).

Research involving human participants, their data, or biological material

Policy information about studies with human participants or human data. See also policy information about sex, gender (identity/presentation), and sexual orientation and race, ethnicity and racism.

Reporting on sex and gender	Not Applicable
Reporting on race, ethnicity, or other socially relevant groupings	Not Applicable
Population characteristics	Not Applicable
Recruitment	Not Applicable
Ethics oversight	Not Applicable

Note that full information on the approval of the study protocol must also be provided in the manuscript.

Field-specific reporting

Please select the one below that is the best fit for your research. If you are not sure, read the appropriate sections before making your selection.

[x] Life sciences   [ ] Behavioural & social sciences   [ ] Ecological, evolutionary & environmental sciences

For a reference copy of the document with all sections, see nature.com/documents/nr-reporting-summary-flat.pdf

Life sciences study design

All studies must disclose on these points even when the disclosure is negative.

Sample size	We used at least 50 animals per replicate for most experiments, with at least three replicates performed. For experiments with different sample sizes, the exact numbers are provided in the Materials and Methods section.
Data exclusions	Not Applicable
Replication	A minimum of three biological replicates was used for most experiments. For those with more or fewer replicates, the number is clearly specified in the Materials and Methods and figure legends sections.
Randomization	Samples in this study were selected randomly.
Blinding	Sample collection was performed blindly and without bias.

Reporting for specific materials, systems and methods

We require information from authors about some types of materials, experimental systems and methods used in many studies. Here, indicate whether each material, system or method listed is relevant to your study. If you are not sure if a list item applies to your research, read the appropriate section before selecting a response.

Materials & experimental systems

n/a	Involved in the study	
[ ]	[x]	Antibodies
[ ]	[x]	Eukaryotic cell lines
[x]	[ ]	Palaeontology and archaeology
[ ]	[x]	Animals and other organisms
[x]	[ ]	Clinical data
[x]	[ ]	Dual use research of concern
[x]	[ ]	Plants

Methods

n/a	Involved in the study	
[x]	[ ]	ChIP-seq
[x]	[ ]	Flow cytometry
[x]	[ ]	MRI-based neuroimaging

Antibodies

Antibodies used	All antibodies used in this study are commercially available and are described in detail in the Materials and Methods section. Specifically, we used: Rabbit anti-Flag (Cell Signaling, #14793S), Mouse anti-Flag (Cell Signaling, #8146S), Rabbit anti-Myc (Cell Signaling, #2278S), Alexa Fluor 488 (Abcam, #ab150077), and Rabbit IgG H&L HRP-conjugated secondary antibody (Abcam, #ab97051).

Validation	All antibodies are commercially available and validated by the manufacturers. Catalogue numbers are provided in the Materials and Methods section.

Eukaryotic cell lines

Policy information about cell lines and Sex and Gender in Research

Cell line source(s)	In this study, we exclusively used Drosophila Schneider 2 (S2) cells, which are derived from primary cultures of Drosophila embryos.
Authentication	Not Applicable
Mycoplasma contamination	Not Applicable
Commonly misidentified lines (See ICLAC register)	Not Applicable

Animals and other research organisms

Policy information about studies involving animals; ARRIVE guidelines recommended for reporting animal research, and Sex and Gender in Research

Laboratory animals	We only used Drosophila melanogaster and Saccharomyces cerevisiae in this study.
Wild animals	Not Applicable
Reporting on sex	Not Applicable
Field-collected samples	Not Applicable
Ethics oversight	Not Applicable

Note that full information on the approval of the study protocol must also be provided in the manuscript.

Plants

Seed stocks	Not Applicable
Novel plant genotypes	Not Applicable
Authentication	Not Applicable
